# MAPK phosphatase 1 inhibition of p38**α** within lung myofibroblasts is essential for spontaneous fibrosis resolution

**DOI:** 10.1172/JCI172826

**Published:** 2024-03-21

**Authors:** Sean M. Fortier, Natalie M. Walker, Loka R. Penke, Jared D. Baas, Qinxue Shen, Jennifer M. Speth, Steven K. Huang, Rachel L. Zemans, Anton M. Bennett, Marc Peters-Golden

**Affiliations:** 1 Division of Pulmonary and Critical Care Medicine, University of Michigan Medical School, Ann Arbor, Michigan, USA.; 2 Department of Pulmonary and Critical Care Medicine, The Second Xiangya Hospital, Central South University, Changsha, Hunan, China.; 3 Department of Pharmacology, Yale University School of Medicine, New Haven, Connecticut, USA.

**Keywords:** Cell biology, Pulmonology, Fibrosis, Molecular biology, Protein kinases

## Abstract

Fibrosis following tissue injury is distinguished from normal repair by the accumulation of pathogenic and apoptosis-resistant myofibroblasts (MFs), which arise primarily by differentiation from resident fibroblasts. Endogenous molecular brakes that promote MF dedifferentiation and clearance during spontaneous resolution of experimental lung fibrosis may provide insights that could inform and improve the treatment of progressive pulmonary fibrosis in patients. MAPK phosphatase 1 (MKP1) influences the cellular phenotype and fate through precise and timely regulation of MAPK activity within various cell types and tissues, yet its role in lung fibroblasts and pulmonary fibrosis has not been explored. Using gain- and loss-of-function studies, we found that MKP1 promoted lung MF dedifferentiation and restored the sensitivity of these cells to apoptosis — effects determined to be mainly dependent on MKP1’s dephosphorylation of p38α MAPK (p38α). Fibroblast-specific deletion of MKP1 following peak bleomycin-induced lung fibrosis largely abrogated its subsequent spontaneous resolution. Such resolution was restored by treating these transgenic mice with the p38α inhibitor VX-702. We conclude that MKP1 is a critical antifibrotic brake whose inhibition of pathogenic p38α in lung fibroblasts is necessary for fibrosis resolution following lung injury.

## Abstract 2

By inhibiting p38&alpha; MAPK, MKP1 serves as a previously unrecognized brake on myofibroblast function and is essential for spontaneous resolution of lung fibrosis.

## Graphical abstract

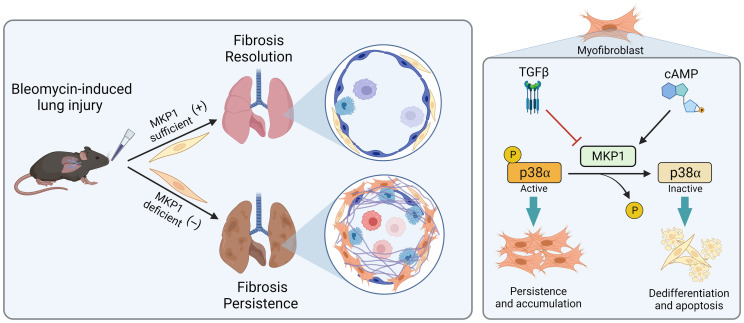

## Introduction

Fibrosis is a disordered response to tissue injury characterized by abnormal wound healing and organ scarring. In pulmonary fibrosis, collagen accumulation and contraction of the parenchyma distort lung architecture and interfere with gas exchange. The prototypic fibrotic lung disease — idiopathic pulmonary fibrosis (IPF) — is a common and severe disorder typically progressing to respiratory failure and death within 3–5 years (1, 2). The ultimate effector cell in IPF is the myofibroblast (MF) — a distinct mesenchymal cell derived from the differentiation of resident lung fibroblasts that contributes to lung scarring and stiffness through secretion of collagens and matrix proteins and expression of contractile stress fibers and focal adhesions, respectively (3, 4).

Because patients with IPF invariably manifest established scarring at the time of clinical presentation, a truly impactful treatment strategy must not only prevent progression but also reverse existing fibrosis. A key feature of MFs that enables fibrosis to endure and progress in IPF and other fibrotic disorders is their resistance to apoptosis and subsequent accumulation (5, 6) — in glaring contrast with their timely clearance during normal wound healing (7, 8). Indeed, it has been demonstrated that spontaneous fibrosis resolution — a characteristic and in some respects problematic feature of the bleomycin mouse model of pulmonary fibrosis — requires Fas-mediated lung MF apoptosis (9). Although once considered irreversible, it is now well recognized that in vitro dedifferentiation of the MF phenotype can proceed via a variety of molecular pathways (10–15). Importantly, some but not all of these pathways concomitantly restore MF sensitivity to apoptosis (16). However, the endogenous molecular mediators and signaling pathways that promote MF dedifferentiation and enable their apoptosis, thus recapitulating normal wound healing and potentially enabling fibrosis resolution following lung injury, remain unknown. Furthermore, the capacity of the 2 currently approved pharmacotherapies for IPF, pirfenidone and nintedanib, to dedifferentiate established MFs has not been explored.

The MAPKs comprise 3 families of constitutively expressed proteins — p38 MAPK (p38), JNK, and ERK — that are known to influence numerous biological processes including fibroblast differentiation, survival, and proliferation (17–19). Precise spatiotemporal regulation of MAPKs is crucial and requires their posttranslational phosphorylation and dephosphorylation (20, 21). Profibrotic stimuli, such as TGF-β1 (TGF-β), and fibrotic states are associated with increased levels of activated p38, JNK, and ERK within lung fibroblasts (22–25). However, the influence of these MAPKs on MF plasticity, and thus their potential role in maintaining persistent fibrosis, is not known (26).

The MAPK phosphatases (MKPs, also known by their gene category name dual-specificity phosphatases [DUSPs]) dephosphorylate the regulatory tyrosine and threonine residues of MAPKs and thus function as a key brake on their activity (27, 28). MKP1 (encoded by *DUSP1*) is one of 10 catalytically active MKPs expressed in mammalian cells and belongs to the “inducible nuclear” subclass (27). Following mitogen- or stress-induced stimulation, MKP1 is rapidly upregulated and localizes to the nucleus, where it is known to regulate cell phenotypes in several tissues, including skeletal/cardiac muscle, liver, bone, and brain (29–33). We have previously linked the antifibrotic actions of the drug bortezomib to its ability to induce the expression of MKP1 (but not other MKP proteins) in lung fibroblasts (34). However, the specific effect of MKP1 on MF phenotype, its downstream MAPK effector targets, and its role as an endogenous brake on pulmonary fibrosis remains unexplored.

In the present study, we utilized in vitro lentiviral transduction techniques to generate inducible MKP1-overexpressing and CRISPR/Cas9-mediated, MKP1-deficient human lung fibroblasts (HLFs) and demonstrate that MKP1 inhibition of p38α, but not JNK or ERK, led to MF dedifferentiation in vitro and restored sensitivity to apoptosis. Moreover, Cre recombinase–mediated deletion of *Dusp1* in mouse fibroblasts initiated following peak bleomycin-induced pulmonary fibrosis abrogated spontaneous resolution in vivo*,* indicating that MKP1 served as a critical antifibrotic brake promoting fibrosis resolution. Indeed, fibrosis resolution was restored in mouse lungs whose fibroblasts lacked MKP1 following administration of a p38α-specific inhibitor.

## Results

### MKP1 (DUSP1) is the chief DUSP isoform expressed in normal lung fibroblasts, is reduced in IPF fibroblasts, and is downregulated by TGF-β.

**Figure 1:**
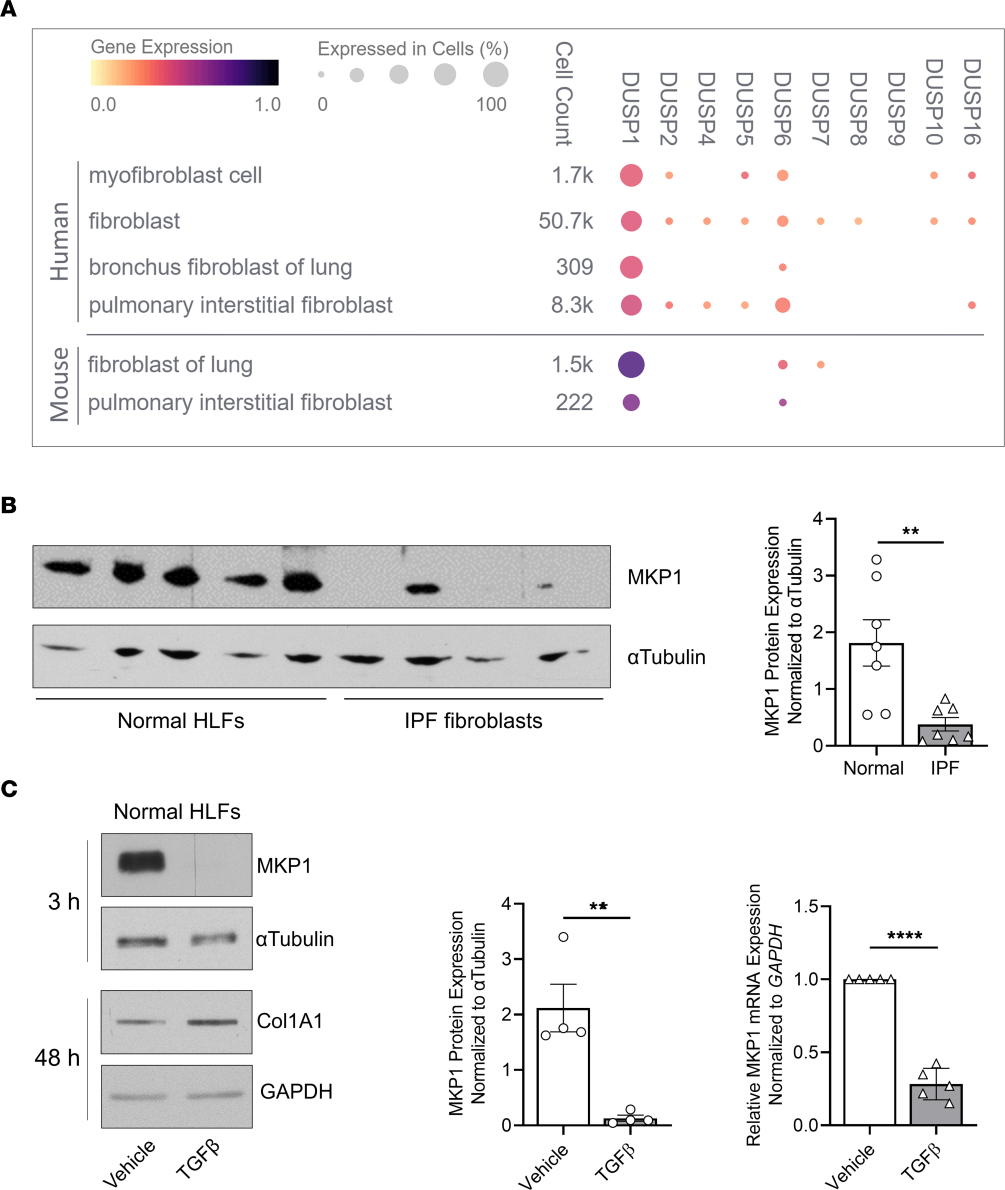
MKP1 (*DUSP1*) is the chief DUSP isoform expressed in normal lung fibroblasts, is reduced in IPF fibroblasts, and is downregulated by TGF-β. (**A**) Single-cell RNA-Seq of annotated human and mouse lung fibroblast populations (generated from the Chan-Zuckerberg CELL by GENE Discover online database) depicting the relative expression of each catalytically active DUSP/MKP gene. Circle color denotes the mean gene expression within each fibroblast subtype, and the circle size represents the proportion of each cell population expressing the indicated gene. (**B**) MKP1 protein expression measured by Western blotting in normal and IPF patient–derived human lung fibroblasts (left: representative blot of individual patient-derived cells; right: densitometric analysis of all such cells). (**C**) MKP1 and collagen I protein expression by Western blotting (left and middle) and MKP1 transcript by qPCR (right) in normal HLFs treated with TGF-β (2 ng/mL) for 3 hours or 48 hours, as indicated in **C**. Data points represent separate experiments. Significance for densitometric data in **B** (*n* = 7) and **C** (*n* = 4) and qPCR in **C** (*n* = 5) was determined by 2-tailed *t* test. **P < 0.01 and ****P < 0.0001.

Using the Chan Zuckerberg Initiative online database (CZ CELL x GENE, https://cellxgene.cziscience.com/), we determined the relative prevalence and levels of expression of transcripts encoding each of the catalytically active DUSP proteins among available human and mouse lung fibroblast subtypes (Figure 1A). *DUSP1* was the most commonly expressed and abundant isoform measured in fibroblasts of normal lungs from both humans and mice, and its expression was also noted in several other cell types (Supplemental Figure 1, A and B; supplemental material available online with this article; https://doi.org/10.1172/JCI172826DS1). Analysis of publicly available single-cell RNA-Seq data in the Idiopathic Pulmonary Fibrosis Cell Atlas (http://www.ipfcellatlas.com/) did not reveal substantial differences in MKP1 transcript levels in any fibroblast subtype between normal lungs and those with IPF or interstitial lung disease (Supplemental Figure 1, C and D). However, we found that MKP1 protein expression in IPF fibroblasts was reduced when compared with that in normal HLFs (Figure 1B), suggesting possible differences in posttranscriptional regulation of MKP1 in IPF versus normal HLFs. In keeping with this observation, treatment of normal HLFs with the pivotal profibrotic mediator TGF-β reduced MKP1 protein and mRNA concomitant with an increase in collagen 1A1 (Col1A1) protein (Figure 1C). However, this effect was transient, demonstrating a nadir 3 hours after TGF-β treatment with a return of transcript and protein expression levels to baseline at 24 hours (Supplemental Figure 1, E and F). Such rapid and labile modulation of MKP1 expression is consistent with the strict temporal regulation of MKPs in other cell types (35–37). These data thus demonstrate that lung fibroblast MKP1 protein expression was reduced in cells from patients with pulmonary fibrosis and in those exposed to a fibrotic stimulus well known to be abundant in fibrotic lungs (38).

### MKP1 deletion in lung fibroblasts potentiates collagen production, while its overexpression leads to MF dedifferentiation and restored sensitivity to apoptosis.

**Figure 2:**
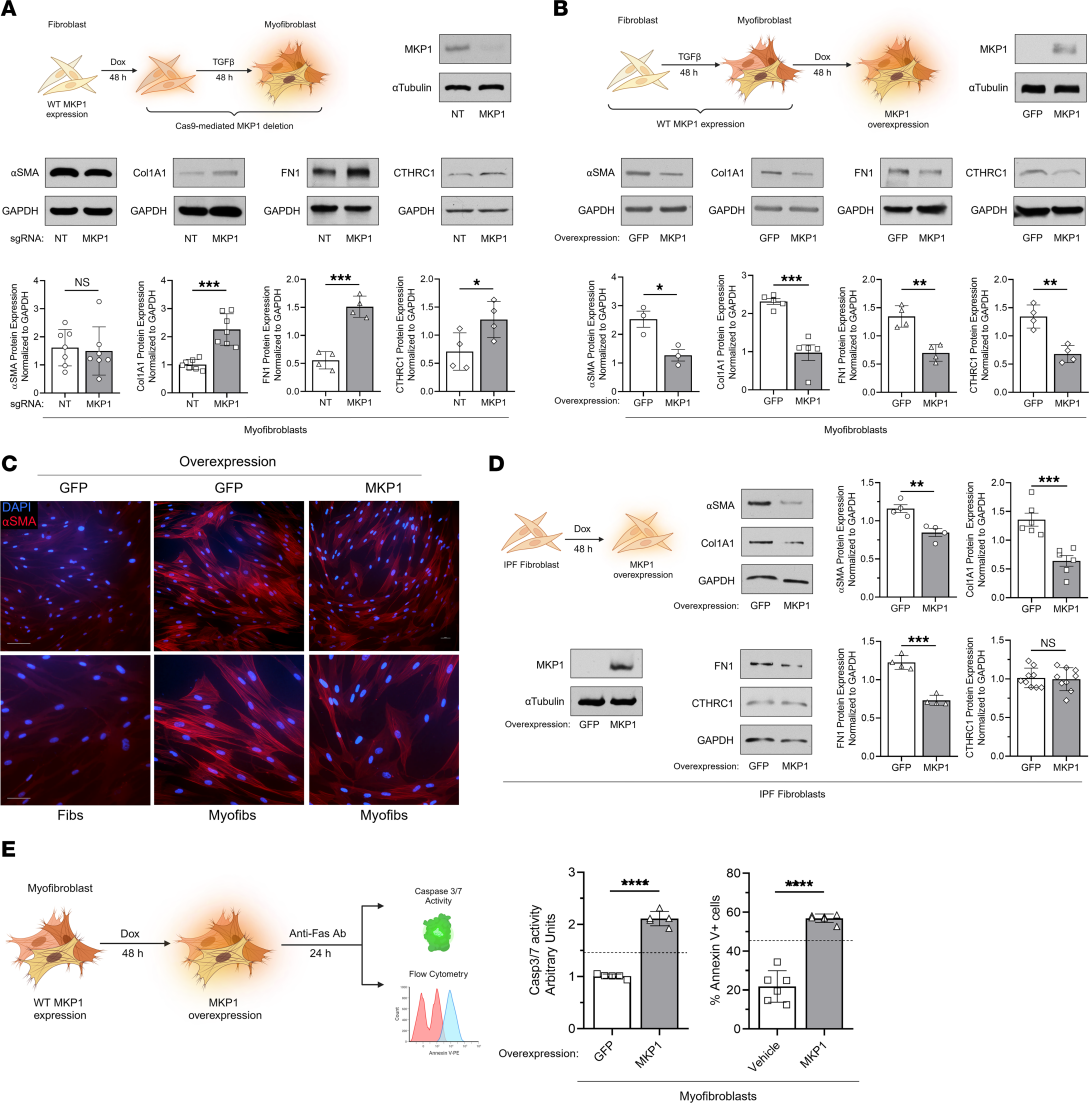
Effect of gain- and loss-of-function of MKP1 on lung fibroblast phenotypic features. (**A**) CRISPR/Cas9-mediated *DUSP1* deletion using MKP1 or NT sgRNAs in HLF-derived MFs. Protein quantification by Western blotting of MKP1 and the fibrosis-associated genes αSMA, Col1A1, FN1, and CTHRC1 (top: representative blot; bottom: densitometric analysis). (**B** and **C**) Inducible MKP1 overexpression in human lung MFs. (**B**) Protein quantification by Western blotting of MKP1 and the fibrosis-associated genes αSMA, Col1A1, FN1, and CTHRC1 (top: representative blot; bottom: densitometric analysis). (**C**) αSMA stress fibers identified by immunofluorescence microscopy using an anti–αSMA-Cy3–conjugated antibody in MFs (Myofibs) overexpressing MKP1 or GFP and fibroblast (Fibs) controls (using the same protocol as in **B**). Nuclei were stained with DAPI. Scale bars: 20 μm (top row) and 10 μm (bottom row). (**D**) IPF fibroblasts harboring the same MKP1 overexpression construct as normal HLFs in **B** were treated with doxycycline for 48 hours to induce MKP1 or GFP overexpression. Protein quantification by Western blotting of MKP1 and the fibrosis-associated genes αSMA, Col1A1, FN1, and CTHRC1 (left: representative blots, right: densitometric analysis). (**E**) Apoptosis sensitivity in MKP1-overexpressing (or vehicle-treated) MFs further treated with an anti-Fas–activating antibody (100 ng/mL) for 24 hours. Apoptosis was determined by caspase-3/-7 activity assay (left) or annexin V expression (right). Dashed lines represent caspase-3/-7 activity or annexin V expression in untreated, undifferentiated fibroblasts stimulated with anti-Fas antibody. The sample number for each experiment (*n*) varied between 3 and 9 and is indicated by the number of data points in each histogram. Each blot grouping containing a protein (or proteins) of interested and its corresponding loading control were run on separate gels. Significance for densitometric analysis and apoptosis activity assays was determined by 2-tailed *t* test. *P < 0.05, **P < 0.01, ***P < 0.001, and ****P < 0.0001. Dox, doxycycline.

To evaluate the role of baseline MKP1 expression in canonical fibrosis–associated gene expression in vitro, we infected normal HLFs with lentivirus containing a lentiCRISPR plasmid (TLCV2) with a constitutively expressed sgRNA targeting *DUSP1*/MKP1 (or a nontargeting [NT] sgRNA control) and doxycycline-inducible Cas9. After pretreatment for 48 hours with doxycycline, fibroblasts were treated with TGF-β to promote MF differentiation. Doxycycline treatment efficiently reduced MKP1 expression, leading to an increase in TGF-β–induced expression of Col1A1, fibronectin (FN1), and the fibroblast activation–associated gene collagen triple helix repeat–containing 1 (CTHRC1) without a significant change in α–smooth muscle actin (αSMA) protein (Figure 2A). MKP1-deficient HLFs also exhibited reduced caspase-3/-7 activity following exposure to an anti-Fas–activating antibody (Supplemental Figure 2B). Given that these data showed that MKP1 negatively affected the activation of fibroblasts and reduced their sensitivity to apoptosis, we reasoned that its overexpression might promote MF dedifferentiation and restore apoptosis sensitivity in these cells. To test this hypothesis, we transduced normal HLFs with lentivirus containing an inducible MKP1 (or GFP) overexpression construct, treated these cells with TGF-β to generate MFs, and subsequently induced MKP1 overexpression with doxycycline. As expected, we detected a substantial increase in MKP1 transcripts 24 hours after doxycycline treatment (Supplemental Figure 2A). MKP1 protein expression in these MFs was also markedly increased and associated with a reduction in αSMA, Col1A1, FN1, and CTHRC1 protein and RNA expression (Figure 2B and Supplemental Figure 2A). Organized αSMA stress fiber content, as indicated by immunofluorescence staining, was likewise reduced in MFs overexpressing MKP1 (Figure 2C). Similarly, MKP1 overexpression in otherwise untreated IPF fibroblasts led to a reduction in αSMA, Col1A1, and FN1 protein expression without a change in CTHRC1 expression (Figure 2D). Crucially, MKP1 overexpression increased Fas-induced caspase-3/-7 activity and annexin V membrane expression (Figure 2E), indicating that MKP1 upregulation restored apoptosis sensitivity in MFs in vitro. These results demonstrate that MKP1 can dedifferentiate MFs toward an apoptosis-sensitive phenotype.

### Lung fibroblast expression of MKP1 mitigates peak fibrosis and is essential for spontaneous fibrosis resolution following in vivo administration of bleomycin.

Given that MKP1 serves as an antifibrotic brake in vitro by promoting MF dedifferentiation and restoring Fas-mediated apoptosis, we hypothesized that its constitutive expression in lung fibroblasts mitigates the severity of peak fibrosis and is necessary for spontaneous fibrosis resolution following lung injury in vivo. To address these hypotheses, we utilized a previously reported C57BL/6 transgenic mouse strain with *loxP* regions spanning exons 2 and 3 of *Dusp1* (39) and crossed it with C57BL/6 mice containing an inducible fibroblast-specific Cre (40). The resulting *Col1a2^CreERT2^* *Dusp1^fl/fl^* mice (or their Cre^–^ littermate controls) were treated with tamoxifen at the indicated time points following bleomycin-induced lung injury to specifically delete MKP1 in fibroblasts.

**Figure 3:**
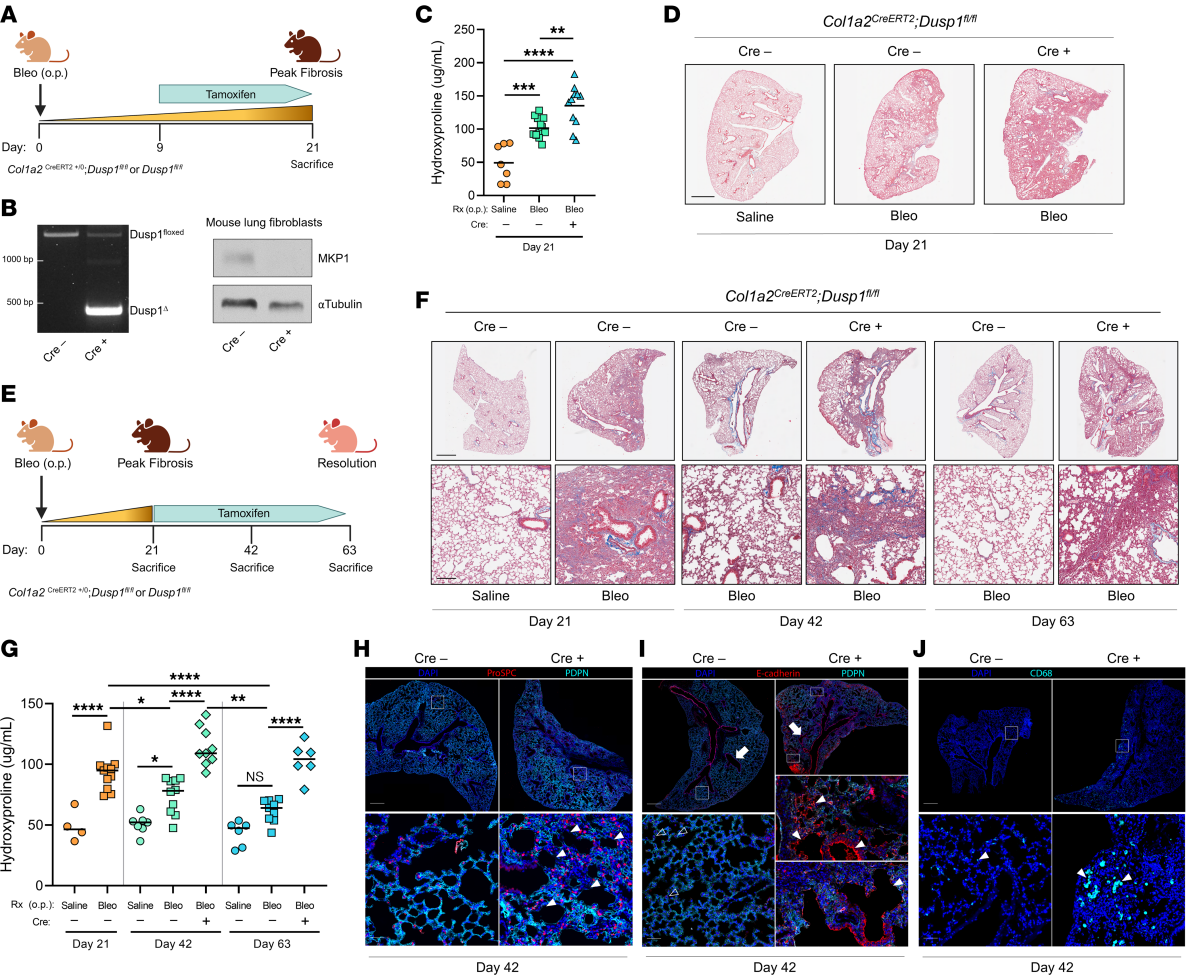
Lung fibroblast expression of MKP1 mitigates peak fibrosis and is essential for spontaneous fibrosis resolution following in vivo administration of bleomycin. (**A**) Schematic illustrating the peak fibrosis protocol. (**B**) PCR of the *Dusp1* locus in Cre^–^ and Cre^+^ mouse tails following tamoxifen administration (left), and subsequent MKP1 protein by Western blot in Cre^–^ or Cre^+^ cultured lung fibroblasts (right). (**C**) Hydroxyproline content quantified from the left and right upper/middle lobe lung homogenates in saline-treated, bleomycin-treated Cre^–^ , and bleomycin-treated Cre^+^ mice on day 21. (**D**) Representative images of Masson’s trichrome staining of the right lower lobe from the same mice used in **C**. Scale bar: 1 mm. (**E**) Schematic illustrating the resolution protocol. (**F**) Representative images of Masson’s trichrome staining of the right lower lobe in saline- and bleomycin-treated WT Cre^–^ mice on day 21 and bleomycin-treated Cre^–^ or bleomycin-treated Cre^+^ mice on days 42 or 63. Scale bars: 1 mm (top row) and 100 μm (bottom row). (**G**) Hydroxyproline content quantified in left and right upper/middle lobe lung homogenates from the same mice in **F**. (**H**–**J**) Immunofluorescence microscopy images of bleomycin-treated mice at mid-resolution (day 42) depicting type I (PDPN) and type II (pro-SPC) alveolar epithelial cells (**H**), parenchymal bronchiolization (E-cadherin) (**I**), and alveolar macrophages (CD68) (**J**). White arrows in **I** depict normal airways. Open white arrowheads depict E-cadherin staining of type II alveolar epithelial cells. Solid white arrowheads point to alveolar regions devoid of type I cells and type II cell hyperplasia in **H**, regions of parenchymal bronchiolization in **I**, and alveolar macrophages in **J**. Scale bars: 500 μm (top row) and 50 μm (bottom row). Each data point represents an individual mouse. Significance for hydroxyproline was determined by 1-way ANOVA. *P < 0.05, **P < 0.01, ***P < 0.001, and ****P < 0.0001. Bleo, bleomycin.

To assess the role of MKP1 in lung fibroblasts during fibrogenesis*, Col1a2^CreERT2^* *Dusp1^fl/fl^* and *Dusp1^fl/fl^* mouse lungs were instilled with bleomycin (1.0 U/kg) by oropharyngeal (o.p.) administration (day 0), followed by introduction of a tamoxifen chow diet starting on day 9, with sacrifice and lung harvest on day 21 (Figure 3A). The time frame of days 9–21 was chosen to reflect the postinflammatory fibrotic phase in this model. Tamoxifen successfully promoted *loxP* recombination of the *Dusp1^fl/fl^* locus (Figure 3B, left) resulting in a near-complete elimination of MKP1 protein (Figure 3B, right) in Cre^+^ mice compared with Cre control mice. As expected, bleomycin induced an approximate doubling of hydroxyproline content in Cre^–^ mouse lungs at day 21 (Figure 3C). Consistent with our in vitro finding that MKP1 deletion in MFs promoted higher collagen I expression (Figure 2A), bleomycin-treated lungs from Cre^+^ mice had significantly greater hydroxyproline content than did Cre^–^ mice (Figure 3C). Histopathologic analysis using Masson’s trichrome staining of collagens (Figure 3D) further indicated an increase in the severity of pulmonary fibrosis in Cre^+^ mice.

Pulmonary fibrosis arising from single-dose intrapulmonary bleomycin is well known to eventually resolve spontaneously in the lungs of young mice (9, 41, 42). Although this has often been viewed as a limitation of the model, it provides a unique opportunity to identify and study endogenous antifibrotic brakes whose deletion might abrogate such spontaneous resolution. We therefore sought to determine the role of lung fibroblast MKP1 during the resolution phase of bleomycin-induced pulmonary fibrosis. To specifically assess MKP1 during resolution, tamoxifen chow was provided to *Col1a2^CreERT2^* *Dusp1^fl/fl^* and *Dusp1^fl/fl^* mice following the establishment of bleomycin-induced peak fibrosis (at day 21) and maintained until sacrifice on day 42 or 63 (Figure 3E). As expected, and as measured by hydroxyproline quantitation of lung collagen content, Cre^–^ mice demonstrated partial fibrosis resolution at day 42 and near-complete resolution by day 63 (Figure 3G). By contrast, Cre^+^ mice showed no decrease in lung collagen content by days 42 or 63, indicating a failure of spontaneous fibrosis resolution (Figure 3G). Failure of spontaneous resolution was also demonstrated by histopathology in Cre^+^ mice at days 42 and 63 (Figure 3F). We performed immunofluorescence microscopic analysis of lung sections from mice harvested at each time point to assess the kinetics of MKP1 and αSMA expression during resolution. Similar to the transient TGF-β–mediated reduction of MKP1 in vitro, we found that MKP1 expression was markedly reduced in αSMA^+^ MFs in lung sections obtained from Cre^–^ mice at peak fibrosis but was restored in αSMA^+^ MFs in lung sections from these mice at day 42 (Supplemental Figure 3A). Notably, αSMA expression was far less abundant and stress fibers appeared to be disorganized in fibroblasts expressing MKP1 in Cre^–^ mice at days 42 and 63, consistent with MF clearance and dedifferentiation, respectively. Moreover, αSMA^+^ MFs from Cre^–^ day-42 lung sections displayed positive TUNEL staining, indicating apoptosis-mediated clearance (Supplemental Figure 3B). Consistent with our hydroxyproline and histopathologic data, and in contrast to the findings noted above in Cre^–^ mice, αSMA^+^ MFs remained abundant in lung sections from Cre^+^ mice at days 42 and 63.

Whole-lung mRNA expression of the fibrosis-associated genes *Fn1*, *Tgfb1*, *Cthrc1* (43), *Col3a1*, and antiapoptotic *Birc5* was increased in the lung homogenates of Cre^+^ mice sacrificed on day 42 compared with homogenates of Cre^–^ mice (Supplemental Figure 4A), a finding consistent with the nonresolving nature of fibrosis in mouse lungs whose fibroblasts lack MKP1. We also measured genes from whole-lung lysates specifically associated with fibroblast subtypes (44). Lipofibroblasts were identified by *Plin2* expression, fibrosis injury–associated fibroblasts by *Pdgfrb* expression, and distinct subsets of matrix fibroblasts by *Col13a1* or *Col14a1* expression (Supplemental Figure 4B). With the exception of *Col14a1*, each transcript was markedly upregulated at peak fibrosis. Moreover, *Col13a1* and *Pdgfrb* expression declined more slowly in Cre^+^ mouse lungs and remained significantly higher at day 42 than in lungs from Cre^–^ mice. Interestingly, transcript levels of each gene in Cre^+^ mice were reduced by day 63 to the levels measured in Cre^–^ mouse lungs, despite the persistence of fibrosis demonstrated by lung hydroxyproline content (Figure 3G) and histopathology (Figure 3F). Therefore, whole-lung mRNA levels of fibrosis-associated genes measured on day 42 better reflected global indices of persistent fibrosis/resolution than the levels determined on day 63, perhaps because day 42 represented a more dynamic point of the resolution response. Together, these data indicate that MKP1 within fibroblasts acted as a brake on peak lung fibrosis and was necessary for spontaneous fibrosis resolution.

Cellular crosstalk among mesenchymal, epithelial, and immune cells is an important aspect of tissue repair and homeostasis. We therefore assessed the effect of MKP1 deletion within lung fibroblasts on epithelial cells and macrophages by performing immunofluorescence microscopy at partial resolution (day 42). Lung sections from Cre^–^ mice displayed typical ratios of type I (podoplanin^+^ [PDPN^+^]) and type II (pro–surfactant protein C^+^ [pro-SPC^+^])alveolar epithelial cells, consistent with their grossly normal alveolar structure, whereas Cre^+^ lung sections displayed regions largely devoid of type I cells (Figure 3H). Additionally, abnormal regions of parenchymal bronchiolization (E-cadherin^+^ subpleural tubular structures) were present in Cre^+^ lung sections but completely absent in Cre^–^ lungs (Figure 3I). Likewise, substantially higher numbers of alveolar macrophages (CD68^+^ cells) were present in Cre^+^ mouse lungs compared with Cre^–^ control lungs, indicative of persistent inflammation (Figure 3J). We observed a similar pattern of patchy regions lacking type I epithelial cells associated with macrophage infiltration in lung sections harvested from mice at peak fibrosis (Supplemental Figure 3C). These data demonstrate that the impaired resolution observed in mice lacking MKP1 within the fibroblast compartment was characterized not only by accumulation of activated MFs, but also by dysregulation of epithelial cell and macrophage populations.

### p38 is the MAPK whose inhibition by MKP1 accounts for its ability to dedifferentiate MFs.

**Figure 4:**
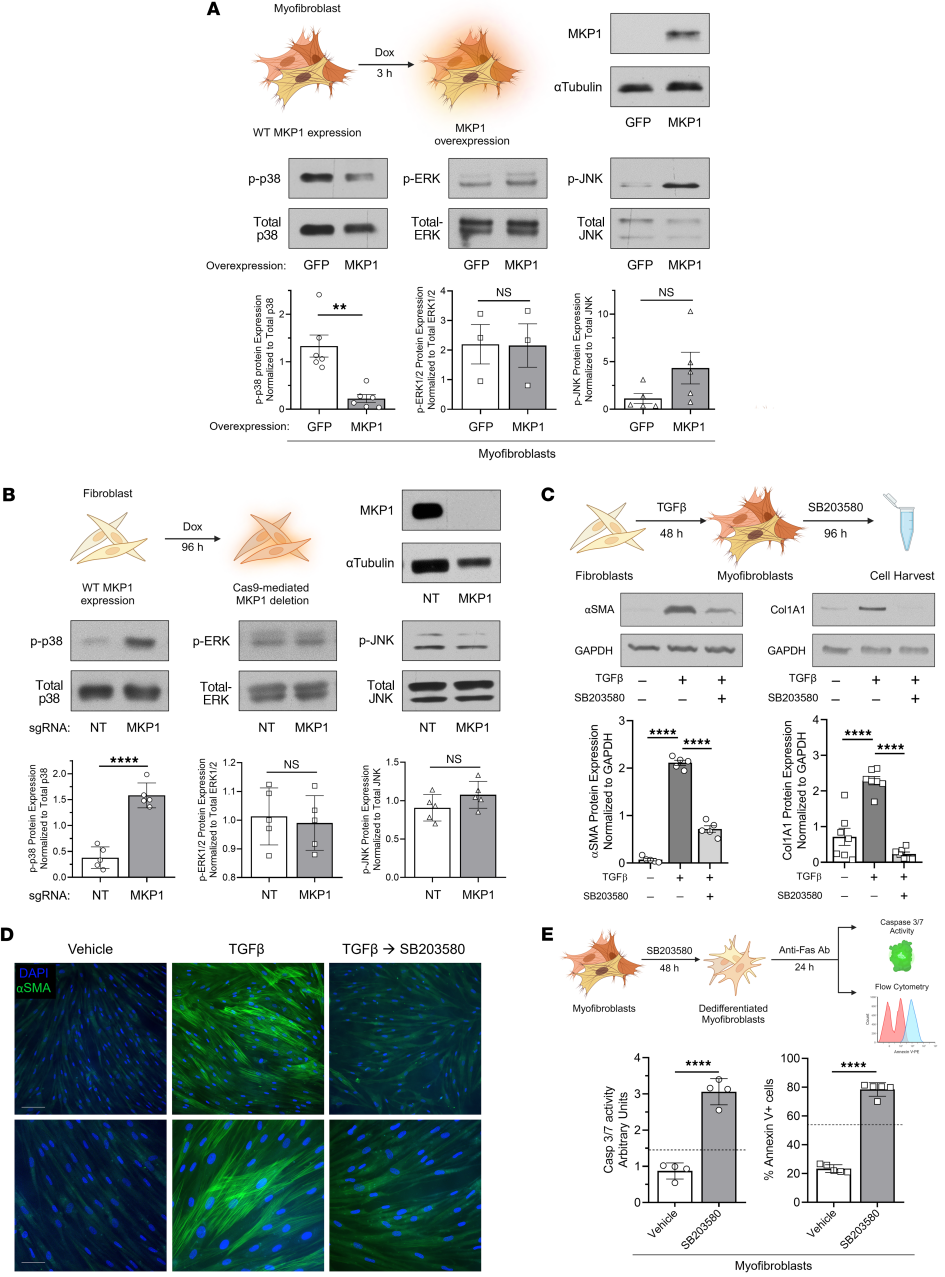
p38 is the MAPK whose inhibition by MKP1 accounts for its ability to dedifferentiate MFs. Effect of MKP1 overexpression (in MFs) or its deletion (in normal HLFs) on MAPK phosphorylation. (**A** and **B**) Western blots of MKP1 and phosphorylated and total MAPK proteins and densitometric analysis. (**C** and **D**) Normal HLFs were treated with TGF-β (2 ng/mL) for 48 hours to generate MFs, followed by treatment with the p38 inhibitor SB203580 (20 μM) for 96 hours. (**C**) Western blot analysis of the fibrosis-associated genes αSMA and Col1A1 and densitometric analysis. (**D**) αSMA stress fibers were identified by immunofluorescence microscopy using an anti–αSMA-FITC–conjugated antibody in MFs treated with SB203580 for 96 hours and fibroblast controls (using the same protocol as in **C**). Nuclei were stained with DAPI. Scale bars: 20 μm (top row) and 10 μm (bottom row). (**E**) Apoptosis sensitivity in SB203580- or vehicle-treated MFs (see protocol schematic) via anti-Fas–activating antibody (100 ng/mL) stimulation for 24 hours. Apoptosis was determined by caspase-3/-7 (Casp 3/7) activity assay (middle) or annexin V expression (right). Dashed lines represent caspase-3/-7 activity or annexin V expression in untreated, undifferentiated fibroblasts treated with anti-Fas. The sample number for each experiment (*n*) varied between 3 and 7 and is indicated by the number of data points in each histogram. Each blot grouping containing a protein of interest and its corresponding loading control were run on separate gels. **P < 0.01 and ****P < 0.0001, by 2-tailed *t* test (**A**, **B**, and **E**) and 1-way ANOVA (**C**).

MKP1 is known to target all 3 MAPK families — p38, JNK, and ERK (27, 45, 46) — but the cellular context and posttranslational modifications greatly influence the degree to which MKP1 interacts with or inhibits each (47, 48). To determine the kinase(s) whose inhibitory targeting is responsible for the ability of MKP1 to promote lung MF dedifferentiation, we assessed MKP1’s ability to dephosphorylate each MAPK in vitro. Utilizing the same MKP1 overexpression strategy as detailed in Figure 2B, we demonstrated that MKP1 promoted the dephosphorylation of p38 (p-p38) (using a Thr180/Tyr182-specific antibody), but not of p-ERK or p-JNK, in TGF-β–elicited lung MFs (Figure 4A). This effect of MKP1 overexpression on p-p38 was also demonstrated in IPF fibroblasts (Supplemental Figure 5A). In a complementary approach, Cas9-mediated deletion of MKP1 led to an increase in p-p38, with no change in p-ERK or p-JNK levels (Figure 4B). Additionally, immunohistochemical staining for p-p38 in mouse lung sections demonstrated greater staining within fibrotic regions in Cre^+^ mice (fibroblast-specific MKP1-null) than in Cre^–^ mice (WT fibroblast MKP1 expression) (Supplemental Figure 5B). To further correlate p-p38 levels with MKP1 over time, patient-derived normal HLFs were treated with TGF-β to activate p38. The TGF-β–mediated increase in p-p38 over 24 hours significantly correlated with a relative decline in MKP1 with a Pearson’s *r* coefficient of –0.64 (Supplemental Figure 5C). Taken together, these data therefore suggest that p38 was the major MAPK directly inhibited by MKP1 in lung MFs within this experimental context.

We next used a distinct experimental strategy to evaluate whether pharmacologic p38 inhibition dedifferentiates lung MFs. Indeed, we found that treatment of MFs with the commonly used p38 inhibitor SB203580 (49) reduced the expression of αSMA and Col1A1 proteins (Figure 4C) and transcripts (Supplemental Figure 5D) as well as the organization of αSMA into stress fibers (Figure 4D). p38 inhibition with SB203580 also restored Fas-mediated apoptosis sensitivity in lung MFs (Figure 4E). These data implicate p38 inactivation as the mechanism by which MKP1 promoted MF dedifferentiation and clearance.

### p38α is the isoform whose inhibition by MKP1 promotes MF dedifferentiation.

**Figure 5:**
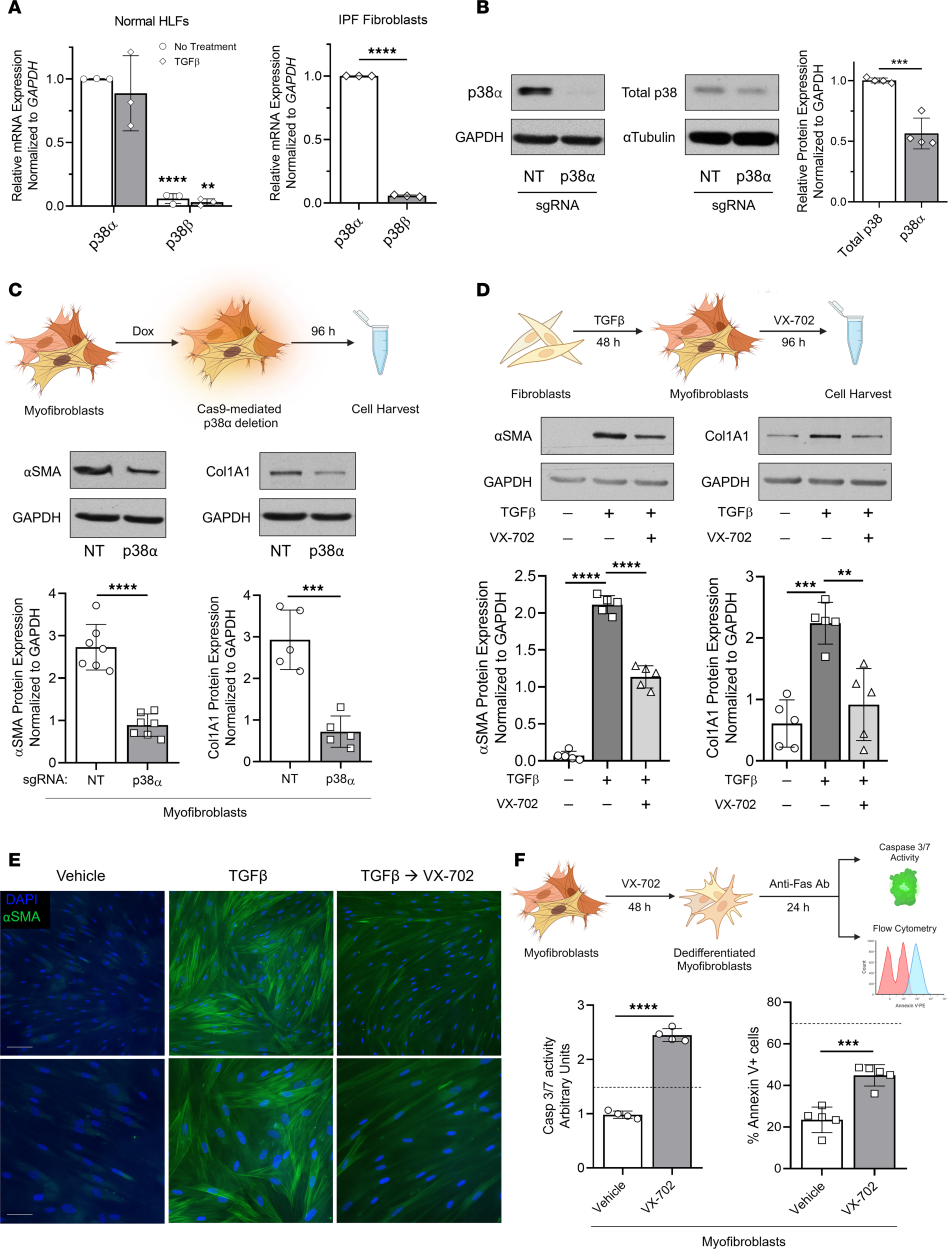
p38α is the isoform whose inhibition by MKP1 promotes MF dedifferentiation. (**A**) Relative p38α and β mRNA expression quantified by qPCR in normal HLFs or MFs and patient-derived IPF fibroblasts. (**B**) Protein quantification of p38α by Western blotting in normal HLFs following Cas9-mediated deletion of p38α using *MAPK14* sgRNA or a NT control. p38α was quantified by subtracting the densitometric value of the total p38 band in the isotype-deleted line from that of the total p38 band of the WT line. (**C**) Western blot analysis of the fibrosis-associated genes αSMA and Col1A1 and densitometric analysis following Cas9-mediated *MAPK14*/p38α deletion. (**D** and **E**) MFs were treatment with the p38 inhibitor VX-702 (50 μM) for 96 hours (protocol schematic is shown in **D**). (**D**) Western blot analysis of αSMA and Col1A1 and densitometric analysis. (**E**) αSMA stress fibers were identified by immunofluorescence microscopy using an anti–αSMA-FITC–conjugated antibody. Nuclei were stained with DAPI. Scale bars: 20 μm (top row) and 10 μm (bottom row). (**F**) Schematic of protocol showing that VX-702– or vehicle-treated MFs were treated with an anti-Fas–activating antibody (100 ng/mL) for 24 hours. Apoptosis sensitivity was determined by caspase-3/-7 activity assay or annexin V expression (right). Dashed lines represent caspase-3/-7 activity or annexin V expression in untreated, undifferentiated fibroblasts incubated with anti-Fas antibody. The sample number for each experiment (*n*) varied between 3 and 5 and is indicated by the number of data points in each histogram. Each blot grouping containing a protein of interest and its corresponding loading control were run on separate gels. **P < 0.01, ***P < 0.001, and ****P < 0.0001 by 2-tailed *t* test (**A**, **B**, and **F)** and 1-way ANOVA (**D**).

There are four p38 isoforms in the mammalian genome — α, β, γ, and δ (50) — with relative expression of each being dependent on tissue and cell type. The α and β isoforms are ubiquitously expressed, whereas γ and δ expression is tissue restricted (51). Moreover, p38α and β are readily dephosphorylated by several MKP proteins, while γ and δ are resistant to all known MKPs (52, 53). We thus measured p38α and β transcript levels by quantitative PCR (qPCR) in normal HLFs, TGF-β–elicited MFs, and IPF fibroblasts (Figure 5A). Each expressed substantially higher (~20-fold) p38α transcript levels compared with p38β. To determine whether p38α protein expression was indeed the most highly translated isoform, we measured the reduction in total p38 protein following CRISPR/Cas9-mediated deletion of *MAPK14* (p38α). This inducible CRISPR/Cas9 line was generated using the same TLCV2 plasmid and the same method to delete *DUSP1* (Figure 2A). Following successful deletion of p38α (Figure 5B, left), we quantified the relative proportion of total p38 attributable to p38α by subtracting the densitometric value of the total p38 band in fibroblasts containing the *MAPK14* sgRNA from that of the total p38 band in the control (Figure 5B, right). Quantification using this approach revealed that p38α accounted for approximately 50% of the total p38 protein expressed in lung MFs. Further analysis using a sgRNA targeting *MAPK12* revealed that p38γ accounted for the vast majority of remaining p38 mRNA and protein (Supplemental Figure 6, A and B), thus suggesting that p38α (as opposed to β) is the predominant target of MKP1 in lung fibroblasts.

To determine whether p38α is indeed the downstream isoform target of MKP1, whose inhibition is responsible for lung MF dedifferentiation, we used the aforementioned inducible p38α CRISPR line. Fibroblasts transduced with lentiCRISPR constructs expressing a *MAPK14*-targeting sgRNA were treated with TGF-β for 48 hours to establish MFs. Dedifferentiation was then assessed following doxycycline-induced Cas9 expression for 96 hours. We found that deletion of p38α promoted MF dedifferentiation, as demonstrated by the substantial reduction in αSMA and Col1A1 protein expression (Figure 5C).

To further confirm the influence of p38α in lung MFs, we applied a pharmacologic approach utilizing VX-702, the isoform-specific inhibitor of p38α (54). We found that elicited MFs treated with VX-702 indeed exhibited significantly reduced αSMA and Col1A1 protein (Figure 5D) and transcript (Supplemental Figure 6C) levels and promoted efficient disassembly of αSMA stress fibers by immunofluorescence microscopy (Figure 5E). Moreover, MFs dedifferentiated with VX-702 showed restoration of apoptosis sensitivity, as indicated by upregulation of cleaved caspase-3/-7 and annexin V (Figure 5F). Similar effects of dedifferentiation and apoptosis were observed in IPF fibroblasts treated with VX-702, independent of MKP1 levels (Supplemental Figure 6, D–F). These complementary data with both knockdown and pharmacologic inhibition indicate that p38α was the likely downstream target of MKP1, whose inhibition was sufficient to dedifferentiate lung MFs and restore their sensitivity to apoptosis.

### The p38α-specific inhibitor VX-702 mitigates bleomycin-induced fibrosis and restores spontaneous fibrosis resolution in mice with MKP1-deficient fibroblasts.

**Figure 6:**
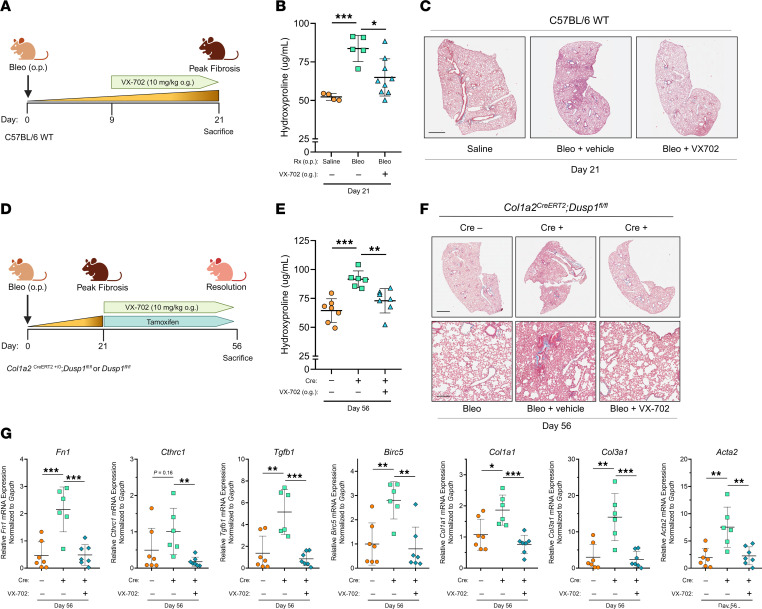
The p38α-specific inhibitor VX-702 mitigates bleomycin-induced fibrosis and restores spontaneous fibrosis resolution in mice with MKP1-deficient fibroblasts. (**A**) Schematic illustrating the peak fibrosis protocol. VX-702 was administered to mice daily by o.g. starting on day 9 through sacrifice on day 21. (**B**) Hydroxyproline content was quantified from left and right upper/middle lobe lung homogenates in saline-, bleomycin-, and bleomycin plus VX-702–treated mice on day 21. (**C**) Representative Masson’s trichrome–stained images of the right lower lobe from the same mice used in **B**. Scale bar: 1 mm. (**D**) Schematic illustrating the resolution protocol. A tamoxifen chow diet was introduced, and VX-702 was administered to Cre^+^ mice by o.g. daily starting on day 21 until sacrifice on day 56. (**E**) Hydroxyproline content was quantified in left and right upper/middle lobe lung homogenates in bleomycin-treated Cre^–^ mice, bleomycin-treated Cre^+^ mice, and bleomycin- plus VX-702–treated Cre^+^ mice (day 56). (**F**) Representative images of Masson’s trichrome staining of the right lower lobe from the same mice used in **E**. Scale bars: 1 mm (top row) and 100 μm (bottom row). (**G**) qPCR of whole-lung expression of *Fn1*, *Cthrc1*, *Tgfb1*, *Birc5*, *Col1a1*, *Col3a1*, and *Acta2* from the same lung homogenates used in **E**. Each data point represents an individual mouse. Significance for hydroxyproline was determined by 1-way ANOVA and for whole-lung RNA by unpaired, 2-tailed *t* test. *P < 0.05, **P < 0.01, and ***P < 0.001.

Having identified a crucial role of p38α as a determinant of the pathogenic MF phenotype in vitro and its robust inhibition by VX-702, we next evaluated the in vivo effect of pharmacologic p38α inhibition during fibrogenesis. Specifically, C57BL/6 male and female mice were treated daily with VX-702 (10 mg/kg) by oral gavage (o.g.) starting on day 9 following bleomycin (1.0 U/kg o.p.) administration until sacrifice and lung harvesting on day 21 (Figure 6A). Bleomycin-treated mice that received VX-702 exhibited less total lung collagen by hydroxyproline content compared with bleomycin-treated controls (Figure 6B). Similarly, peak fibrosis was less severe by histopathology among VX-702–treated mice compared with those treated with bleomycin alone (Figure 6C).

We next sought to test the hypothesis that p38α is a key driver of persistent fibrosis and that its inhibition by MKP1 following peak fibrosis promotes spontaneous fibrosis resolution. The same *Col1a2^CreERT2^* *Dusp1^fl/fl^* mice utilized in the previous in vivo studies (Figure 3) were treated with bleomycin (1.0 U/kg) followed at day 21 by introduction of a tamoxifen diet and daily treatment with VX-702 or vehicle by o.g. (Figure 6D). Mice were sacrificed on day 56 for lung harvesting — a time point at which fibrosis resolution is substantial and similar to that observed at day 63 (data not shown). Consistent with the data displayed in Figure 3, Cre^+^ *Dusp1^fl/fl^* mice treated with vehicle showed substantially higher hydroxyproline content than did Cre^–^ controls, indicating impaired resolution (Figure 6E). Importantly, VX-702 treatment in these Cre^+^ mice reduced hydroxyproline content nearly to the level of Cre^–^ mice exhibiting spontaneous fibrosis resolution (Figure 6E). Histopathology further demonstrated spontaneously resolving fibrosis among Cre^–^ mice, persistent fibrosis in the Cre^+^ cohort, and reversal of persistent fibrosis in Cre^+^ mice treated with VX-702 (Figure 6F). Whole-lung RNA measured in lung homogenates of vehicle-treated Cre^+^ mice at day 56 demonstrated elevated levels of the fibrosis-associated genes *Fn1*, *Tgfb1*, *Acta2*, *Col1a1*, and *Col3a1* as well as elevation of the prosurvival gene *Birc5* and a trend favoring elevation of the pathologic fibroblast marker *Cthrc1*. Remarkably, lung homogenates from Cre^+^ mice treated with VX-702 displayed a significant reduction in the expression of each of these genes to levels seen in Cre^–^ mice (Figure 6G). We measured transcript markers of fibroblast subsets in whole-lung lysates from each condition (Supplemental Figure 7B). Again, we found that *Col13a1* expression was elevated in Cre^+^ mice, but expression levels of *Col14a1*, *Plin2*, and *Pdgfrb* were not significantly different at this time point. Inhibition of p38α via VX-702 substantially reduced expression of the matrix fibroblast markers *Col13a1* and *Col14a1*. These in vivo data combining pharmacologic treatment of fibroblast-specific knockout mice strongly implicated p38α as the fibrotic driver whose inhibition by MKP1 within lung fibroblasts promoted spontaneous fibrosis resolution.

### MKP1 induction is essential for PGE_2_/cAMP/PKA-mediated inhibition of p38 and MF dedifferentiation.

**Figure 7:**
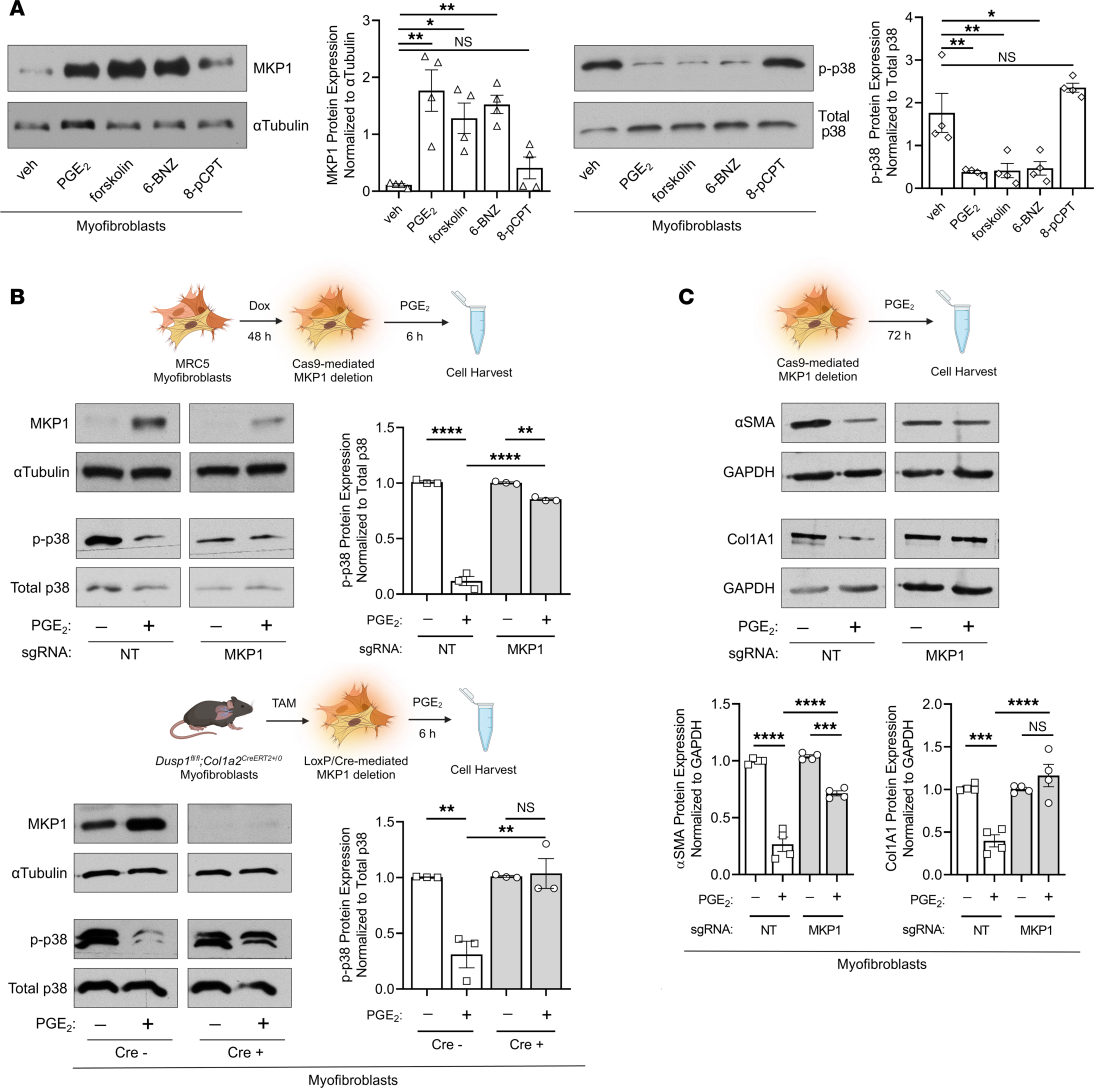
MKP1 induction is essential for PGE_2_/cAMP/PKA-mediated inhibition of p38 and MF dedifferentiation. (**A**) MKP1 and p-p38 protein quantification by Western blotting in MFs treated with PGE_2_ (1 μM), the direct adenylyl cyclase activator forskolin (20 μM), the PKA-specific agonist 6-BNZ-cAMP (2 mM), or the Epac-specific agonist 8-pCPT-cAMP (2 mM) for 6 hours (left: representative blot, right: densitometric analysis). (**B**) MKP1, p-p38, and total p38 expression was quantified by Western blotting in doxycycline-treated lentiCRISPR HLFs containing a *DUSP1*-specific or NT sgRNA (top protocol schematic in **B**) or in lung fibroblasts isolated from naive Cre^+^ or Cre^–^ *Col1a2^CreERT2^* *Dusp1^fl/fl^* mice (bottom protocol schematic in **B**). HLFs and mouse lung fibroblasts were subsequently treated with TGF-β (2 ng/mL for HLFs; 5 ng/mL for mouse lung fibroblasts) for 48 hours to promote the MF phenotype and were then treated with PGE_2_ or vehicle for 6 hours (left: representative blots, right: densitometric analysis). (**C**) Protein quantification of αSMA and Col1A1 seventy-two hours after PGE_2_ treatment of the same lentiCRISPR human MFs generated in **B**. The sample number for each experiment (*n*) varied between 3 and 4 and is indicated by the number of data points in each histogram. Each blot grouping containing a protein of interest and its corresponding loading control were run on separate gels. *P < 0.05, **P < 0.01, ***P < 0.001, and ****P < 0.0001, by 1-way ANOVA.

Our data suggest that MKP1 expression in lung MFs was necessary for spontaneous fibrosis resolution via p38α inhibition in vivo and for their dedifferentiation and clearance in vitro. Mediators and signaling events that might contribute to these proresolution phenomena are of obvious interest. cAMP signaling has been extensively investigated in this context and has been linked with a variety of antifibrotic effects (55). One such cAMP-dependent mediator is prostaglandin E_2_ (PGE_2_), which has been shown to be diminished in the lungs (56) and lung fibroblasts from patients with IPF (57) and to directly promote both MF dedifferentiation (12) and apoptosis (16, 58). PGE_2_ and cAMP have also been shown to induce MKP1 in a variety of cell types (59–61), but whether this is the case in lung fibroblasts, and whether such induction participates in the proresolution effects of PGE_2_ in mesenchymal cells have not been explored to our knowledge. We therefore assessed the effects of PGE_2_ and its canonical downstream cAMP effector molecules on MKP1 induction and p38 inhibition in lung MFs. We found that MKP1 protein expression was rapidly upregulated by PGE_2_, the direct adenylyl cyclase agonist forskolin, and the protein kinase A–specific (PKA-specific) cAMP analog 6-BNZ-cAMP (Figure 7A). Importantly, MKP1 induction by each of these agonists was associated with p38 dephosphorylation. Conversely, the exchange protein activated by the cAMP-specific (Epac-specific) agonist 8-pCPT–cAMP did not affect MKP1 gene expression or modulate p38 phosphorylation. PGE_2_-mediated induction of MKP1 with concomitant p38 dephosphorylation was confirmed in IPF fibroblasts (Supplemental Figure 8).

These results are consistent with our previously published findings that PGE_2_ prevents TGF-β–induced p38 phosphorylation (22) and promotes lung MF dedifferentiation through the cAMP/PKA axis (12, 16), and point to a possible role for MKP1 as a downstream effector of this pathway in lung fibroblasts. To test this possibility, we again applied a CRISPR/Cas9 approach to delete *DUSP1* in lung MFs followed by treatment with PGE_2_. Treatment of control MFs with PGE_2_ was associated with increased MKP1 expression and p38 dephosphorylation. By contrast, PGE_2_ treatment did not promote substantial p38 dephosphorylation in MKP1-null MFs (Figure 7B, top). Using an independent approach, we isolated lung fibroblasts from *Col1a2^CreERT2^* *Dusp1^fl/fl^* and *Dusp1^fl/fl^* mice 10 days after introduction of a tamoxifen chow diet and treated these cells with TGF-β for 48 hours to generate MFs. We then treated both WT and MKP1-null fibroblasts with PGE_2_ (Figure 7B, bottom). Indeed, p38 phosphorylation was greatly reduced in MKP1-expressing fibroblasts treated with PGE_2_ but remained unchanged in MKP1-null mouse lung fibroblasts. Furthermore, MKP1 deletion largely abrogated the PGE_2_-mediated reduction in αSMA protein and abolished its ability to reduce Col1A1 protein expression in human lung MFs (Figure 7C). These findings suggest that MKP1 was necessary for the ability of PGE_2_/cAMP signaling to negatively regulate p38 activity and to dedifferentiate lung MFs.

### The 2 FDA-approved antifibrotic drugs pirfenidone and nintedanib fail to dephosphorylate p38 and to promote dedifferentiation of human lung MFs.

**Figure 8:**
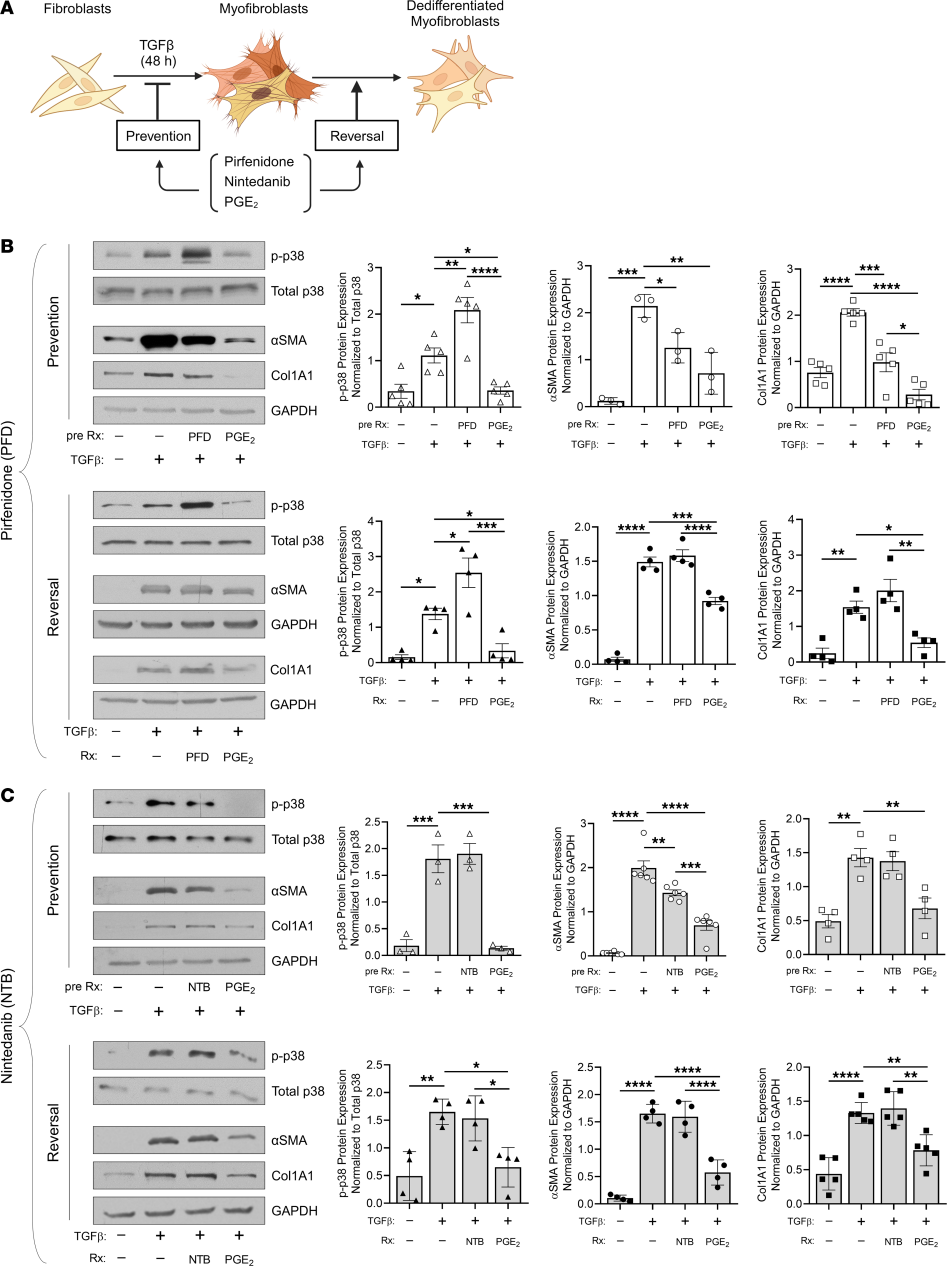
The 2 FDA-approved antifibrotic drugs pirfenidone and nintedanib fail to dephosphorylate p38 and to promote dedifferentiation of human lung MFs. (**A**) Schematic illustrating in vitro MF prevention and MF reversal treatment protocols. Treatment with the antifibrotic agent pirfenidone (1 mM) or nintedanib (2 μM) or the lipid mediator PGE_2_ (1 μM) was administered 15 minutes prior to (prevention) or 48 hours after (reversal) the addition of TGF-β (2 ng/mL) to the culture medium. (**B** and **C**) Protein quantification of p-p38, αSMA, and Col1A1 by Western blotting in normal HLFs following treatment with pirfenidone (**B**) or nintedanib (**C**) compared with PGE_2_ in a prevention or reversal protocol (left: representative blot, right: densitometric analysis). For the prevention studies, proteins were quantified by Western blotting using cell lysates harvested at the following post-treatment time points: p-p38, 6 hours; αSMA and Col1A1, 48 hours. For the reversal studies, proteins were quantified by Western blotting using cell lysates harvested at the following post-treatment time points: p-p38, 24 hours; αSMA, 72 hours; and Col1A1, 48 hours (**B**) or 72 hours (**C**). The sample number for each experiment (*n*) varied between 3 and 6 and is indicated by the number of data points in each histogram. Each blot grouping containing a protein (or proteins) of interest and its corresponding loading control were run on separate gels. *P < 0.05, **P < 0.01, ***P < 0.001, and ****P < 0.0001, by 1-way ANOVA.

Although the current FDA-approved drugs pirfenidone and nintedanib slow the decline in lung function in patients with IPF, they notably fail to resolve established fibrosis (62, 63). Whether these agents elicit dedifferentiation of MFs that may underly fibrosis resolution is unknown. Moreover, their effects on p38 signaling in MFs remain incompletely characterized. Nintedanib might be expected to influence p38 activation through its established inhibition of the FGF, PDGF, and VEGF receptors that signal via MAPKs (64). Pirfenidone, whose mechanism of action is not fully characterized, has been shown to reduce TGF-β signaling (65) and inhibit p38γ (66). We tested the capacity of each of these FDA-approved drugs to prevent fibroblast-to-MF differentiation, promote MF dedifferentiation, and modulate p38 phosphorylation as compared with the documented effects of PGE_2_ (Figure 8A). Cell lysates were harvested at the time points determined to be optimal for each protein of interest. Normal HLFs were pretreated with pirfenidone at a dose of 1 mM, selected on the basis of pilot studies (data not shown) and previously published literature on lung fibroblasts (67). This dose abrogated TGF-β–induced upregulation of αSMA and Col1A1 in a prevention protocol (Figure 8B, top panel). Notably, collagen reduction was less substantial than that of PGE_2_ and was associated with hyperphosphorylation — rather than hypophosphorylation — of p38. Moreover, and in contrast to PGE_2_, pirfenidone treatment of established MFs (reversal protocol) failed to promote their dedifferentiation and likewise resulted in an increase in p-p38 (Figure 8B, bottom panel). Pirfenidone-induced p38 hyperphosphorylation was not associated with modulation of MKP1 protein expression (Supplemental Figure 9A).

Nintedanib was dosed at 2 μM, a dose consistent with its previously published use in vitro (68). This dose of the drug markedly reduced ERK1/2 phosphorylation — a previously reported effect of nintedanib in lung fibroblasts (69) (Supplemental Figure 9B). Otherwise, the effects of nintedanib were similar to those of pirfenidone (Figure 8C). It partially prevented TGF-β–induced αSMA induction but had no effect on Col1A1 and failed to modulate p-p38 levels. Likewise, established MFs treated with nintedanib did not demonstrate evidence of dedifferentiation or changes in p-p38. These data show that neither FDA-approved antifibrotic agent recapitulated the ability of PGE_2_/MKP1 to inhibit p38 and dedifferentiate established MFs in vitro.

## Discussion

The vast preponderance of existing literature concerning lung fibroblasts has focused on the molecular determinants of their activation and differentiation in an effort to identify fibrotic drivers that may be candidate targets for pharmacotherapy. Although this remains an important and worthwhile strategy, far less is known about the brakes capable of promoting MF dedifferentiation and fibrosis resolution. Just as the appearance and persistence of tumor cells typically depend on the combination of oncogene activation and tumor suppressor inactivation, MF accumulation in fibrotic lungs likely involves the concomitant overactivation of drivers and the failure of critical endogenous brakes. Indeed, we have shown that the recognized tumor suppressor Krüppel-like factor 4 (KLF4) likewise serves as an antifibrotic brake in the lung that is deficient in fibrotic disease (40). Nevertheless, relatively little is known about such brakes and their capacity for promoting MF dedifferentiation, clearance, and fibrosis resolution. In this study, we established that expression of the dual-specificity phosphatase MKP1 in lung fibroblasts was critical for spontaneous fibrosis resolution following lung injury. Furthermore, we determined that MKP1 promoted MF dedifferentiation that reestablished apoptosis sensitivity through dephosphorylation of p38α and showed that the specific p38α inhibitor VX-702 restored fibrosis resolution in fibroblast-specific MKP1-null mice. Deletion of MKP1 in fibroblasts following peak fibrosis also impeded alveolar epithelial cell regeneration, promoted parenchymal bronchiolization, and resulted in persistent inflammation, highlighting the importance of cellular crosstalk between fibroblasts and nonmesenchymal cells, as previously noted in other models of nonresolving fibrosis (70). Notably, neither of the FDA-approved antifibrotic drugs was capable of dephosphorylating p38 or promoting MF dedifferentiation. To our knowledge, this is the first study to assess the role of MKP1 in pulmonary fibrosis and the first to specifically characterize its function and regulation by pertinent mediators and pharmacologic agents within fibroblasts.

Dysregulated MAPK activity has been associated with several pathologic inflammatory and fibrotic diseases (71) including pulmonary fibrosis (26). It therefore follows that precise and timely deactivation of MAPKs in key effector cells is crucial for the restoration of tissue homeostasis following the fibrotic phase of lung injury. MKP1 is one of 10 DUSP proteins that regulate MAPK signaling in a variety of mammalian cell types and tissues. The antifibrotic actions of MKP1 in lung fibroblasts in vitro (Figure 2) and its essential role in promoting fibrosis resolution in vivo (Figure 3) presented in this study further confirm the importance of MAPK regulation in pulmonary fibrosis and support the notion that MF dedifferentiation and clearance are necessary for fibrosis resolution. It is notable that both in vitro and in vivo deletion of MKP1 in lung fibroblasts was sufficient to potentiate Col1A1, FN1, and CTHRC1 expression as well as abrogate spontaneous resolution despite the ostensibly redundant MAPK inhibitory activity of other MKP proteins. One possibility for the dominant role of MKP1 in the lung fibroblast phenotype is that other DUSP/MKP genes are expressed at relatively low levels — as suggested by publicly available single-cell data (Figure 1) — and/or are not sufficiently upregulated during resolution. Alternatively, the specific regulatory niche fulfilled by each MKP may be distinct, owing to differences in spatial restriction and substrate selectivity. Consistent with this possibility, and in direct contrast with the actions of MKP1 demonstrated in this work, a previous study revealed that *DUSP10*/MKP5 was crucial for TGF-β–induced SMAD3 activation in lung fibroblasts and was upregulated in IPF fibroblasts, and that its global deletion mitigated bleomycin-induced pulmonary fibrosis (72). These findings highlight the important operative differences among these 2 MKP proteins. In contrast to MKP1, MKP5 lacks a nuclear localization sequence and can thus also influence MAPK activity from the cytoplasm (27). Furthermore, MKP1 and MKP5 have been shown to exhibit opposite regulatory roles in cardiac fibrosis (73) and muscle repair (31), with MKP1 serving as an antifibrotic brake in the heart and as a positive regulator of skeletal muscle myogenesis. The opposing actions of MKP1 and MKP5 are therefore not unprecedented and reinforce the complexity of MKPs in the regulation of cellular phenotypes. Although it remains to be determined whether MKP1 is the only dual-specificity phosphatase that can dedifferentiate MFs and function as a brake on pulmonary fibrosis, our in vivo work suggests that it is the most important endogenous brake among the MKPs.

Remarkably, although MKP1 is known to interact with all 3 MAPKs, our data demonstrate that its overexpression in lung fibroblasts substantially dephosphorylated p38 but not ERK or JNK (Figure 4A). Importantly, deletion of MKP1 led to hyperphosphorylation of p38, suggesting that MKP1 exerted a measurable baseline inhibitory tone on p38 activation in MFs. Further investigation revealed that the p38α isoform was the predominant downstream target of MKP1, given its established inability to inhibit the p38γ and δ isoforms and the paucity of p38β expression within lung fibroblasts (Figure 5). Posttranslational modifications to MKPs are known to influence their affinity for particular MAPK substrates (27) and thus may explain the selectivity of MKP1 for p38 in lung fibroblasts. One potential explanatory mechanism is acetylation of MKP1 at Lys59, which has been demonstrated to substantially increase its affinity for p38 (74). Regulation of protein acetylation itself is a complex process resulting from the net effect of acetyltransferases such as p300 — known to catalyze acetylation of MKP1 at Lys59 (47) — and deacetylases (SIRT proteins) whose actions are known to influence fibroblasts in IPF (75). MKP1 may therefore have different regulatory effects on MAPKs within various cell types in the lung that are dependent on complex contextual inputs on its posttranslational regulation.

The ability of the p38α-specific inhibitor VX-702 to restore fibrosis resolution in mouse lungs whose fibroblasts lack MKP1 (Figure 6) strongly suggests that p38α was indeed the singular p38 isoform driving persistent fibrosis and that its inhibition by MKP1 was required for spontaneous fibrosis resolution. These in vivo data are further supported by our findings that both CRISPR/Cas9-mediated deletion of p38α and the specific p38α inhibitor VX-702 led to MF dedifferentiation (Figure 5, C–E) and restoration of apoptosis sensitivity in vitro (Figure 5F). The ability of VX-702 to mitigate peak fibrosis in vivo suggests that the α isoform also drives pulmonary fibrogenesis. Notably, p38α expression in resident cardiac fibroblasts has been shown to promote their differentiation into MFs and drive cardiac fibrosis following ischemic injury (76), suggesting that p38α exerts a general profibrotic effect in fibroblasts within various tissues. Although our data reveal that fibroblasts represented the primary cell in which inhibition of p38α was likely to account for fibrosis resolution, we cannot exclude possible contributing actions and benefits of inhibiting p38α in other cell types.

The precise timing of MKP1-mediated p38α inhibition during fibrogenesis and fibrosis resolution, as well as what endogenous mediator(s) might upregulate MKP1 expression and/or influence its interaction with p38 remain important unanswered questions. With regard to its upregulation, we found that diminished MKP1 expression was restored in MFs by day 42 — a finding associated with disorganized αSMA stress fibers and apoptosis (Supplemental Figure 3A). Additionally, given that the cAMP/PKA pathway robustly increased MKP1 expression with concomitant p38 dephosphorylation (Figure 7), we speculate that this second messenger pathway might be important for the MKP1 restoration required for MF clearance and fibrosis resolution. Indeed, PGE_2_ — whose antifibrotic actions on MFs are due to cAMP/PKA (16) — required MKP1 to dephosphorylate p38 and promote MF dedifferentiation (Figure 7, B and C). We have also shown that expression of the antifibrotic transcription factor KLF4 is rapidly and substantially increased through the cAMP/PKA pathway (40). Whether the antifibrotic actions of MKP1 and KLF4 depend upon distinct downstream molecular mediators or are interdependent is another important question for future studies.

The FDA-approved drugs pirfenidone and nintedanib fail to halt or reverse the inevitable decline in lung function associated with IPF, thus exposing a major unmet clinical need for therapies that can reverse fibrosis. As MKP1 expression in lung fibroblasts serves as an antifibrotic brake capable of resolving pulmonary fibrosis, pharmacotherapies that increase its expression or directly inhibit p38α would be predicted to be beneficial. To provide further credence for this possibility, we demonstrated that neither pirfenidone nor nintedanib had the ability to dephosphorylate p38 or dedifferentiate MFs (Figure 8). Therefore, MKP1-mediated inhibition of p38α appeared to be mechanistically distinct from the actions of currently available antifibrotic therapies. Furthermore, despite its putative p38γ-inhibitory effect (77), pirfenidone actually promoted total p38 phosphorylation in both a prevention and reversal context in precise contrast to the actions of PGE_2_ (Figure 8B), direct adenylyl cyclase activation, or PKA activation (Figure 7A). It is intriguing that these in vitro results correlate with the apparent superior clinical benefit of a phosphodiesterase 4B inhibitor (which enhances intracellular cAMP levels) in patients with IPF compared with pirfenidone and nintedanib in a recent phase II clinical trial (78). The fact that both of these approved therapies exhibited a modest ability to prevent TGF-β–mediated upregulation of αSMA and Col1A1, but failed to promote MF dedifferentiation, is mechanistically consistent with their ability to slow disease progression while failing to reverse fibrosis.

Collectively, our findings identify MKP1 as an antifibrotic brake in lung fibroblasts, cAMP/PKA as a candidate endogenous pathway promoting MKP1 expression/activity, and p38α as the essential fibrotic driver inhibited by MKP1. Future studies will be necessary to determine the mechanism by which MKP1 expression and p38α activity within lung fibroblasts influence other cell types within the lung, their effect on nonpulmonary fibroblasts, and their influence within other lung disease contexts, such as lung cancer, where MKP1 expression is altered (79). Our study provides insight into the molecular pathways within lung fibroblasts that promote fibrosis resolution following lung injury and thus identifies potential targets for new therapies in IPF that are mechanistically distinct from those of pirfenidone and nintedanib.

## Methods

Additional details on the methods used in this study are provided in the Supplemental Methods.

### Sex as a biological variable.

Our study examined male and female animals, and similar findings are reported for both sexes.

### Animal studies.

WT male and female C57BL/6 mice were obtained from The Jackson Laboratory and used at 8–10 weeks of age. Transgenic mice containing *loxP* sites flanking exons 2 and 3 of the *Dusp1* locus were generated as previously detailed (39). These *Dusp1^fl/fl^* mice were crossed with *Col1a2*^CreERT2+/0^ mice (The Jackson Laboratory, strain no. 029567) to generate tamoxifen-inducible, fibroblast-specific conditional MKP1-KO mice. Genotyping was performed using genomic DNA extracted from tails. Briefly, genomic DNA was extracted using the REDExtract-N-Amp Tissue PCR Kit (MilliporeSigma). Cre genotyping was performed by quantitative PCR (qPCR) using a *Col1a2-*Cre–specific primer pair (forward, 5′-CAGGAGGTTTCGACTAAGTTGG-3′ and reverse, 5′-CATGTCCATCAGGTTCTTGC-3′). PCR primers designed to bind to exon 3 and the 3′ *loxP* were used (5′-GCTGGCAGAGGTCTAGGAGG-3′ and 5′-CATGAGGTAAGCAAGGCAGATG-3′, respectively) to verify the homozygosity of the floxed allele for *Dusp1*. PCR primers designed to flank the *loxP* regions of the *Dusp1^fl^* locus (forward, 5′-TCCTTCTTCGCTTTCAACGC-3′ and reverse, 5′-GCCTGGCAATGAACAAACAC-3′) were used to confirm successful recombination following tamoxifen administration in Cre^+^ mice.

For peak and resolution studies, pulmonary fibrosis was induced in mouse lungs following single-dose o.p. bleomycin administration (1.0 U/kg, MilliporeSigma, B5507), as described previously (40). To determine the role of MKP1 during fibrogenesis or spontaneous fibrosis resolution, its fibroblast-specific deletion was achieved through tamoxifen-induced Cre activation. Specifically, tamoxifen chow (40 mg/mouse/day; Inotiv, TD.130859) was administered starting on day 9 or 21 to delete MKP1 during the fibrotic phase (day 9) or resolution phase (day 21) and continued until harvest at the indicated time points. To assess the role of p38α during fibrogenesis in WT mice or the resolution phase in mice whose fibroblasts lacked MKP1, VX-702 (10 mg/kg o.g.) was administered daily starting on day 9 or 21 until sacrifice. Mice were sacrificed on days 21, 42, 56, or 63 as indicated. Lungs were perfused with cold PBS, and the right upper, middle, and left lung lobes were harvested to assess fibrotic endpoints. Specifically, lung lobes were combined in PBS, homogenized, and assessed for hydroxyproline content to estimate total collagen and whole-lung RNA, while the right lower lobe was used to generate fibroblast cultures in vitro or sectioned to perform Masson’s trichrome staining, followed by imaging with bright-field, whole-slide imaging with a Vectra Polaris Brightfield Scanner.

### Gain- and loss-of-function studies.

MKP1- or GFP-overexpressing fibroblast lines were generated by infection with lentiviral particles containing pLVX-TetOne-Puro-hDUSP1 or pLVX-TetOne-Puro-eGFP (Addgene) plasmids. Stable inducible fibroblast lines capable of CRISPR/Cas9-mediated deletion of MKP1, p38α, and p38γ were generated via infection with lentiviral particles containing pLentiCRISPR v2 TLCV2 plasmids (Addgene no. 87360) controlling the following sgRNA sequences: *DUSP1*, 5′-CGTCCAGCAACACCACGGCG-3′; *MAPK14*, 5′-CACAAAAACGGGGTTACGTG-3′; and *MAPK12*, 5′-CTCATGAAACATGAGAAGCT-3′. The NT sequence 5′-AAATGTGAGATCAGAGTAAT-3′ (Thermo Fisher Scientific, A35526) was likewise incorporated into TLCV2 and used as a negative control. Polybrene (2 μg/mL, MilliporeSigma, TR-1003-G) or protamine sulfate (8 μg/mL, MilliporeSigma, P4020) was added to lentiviral suspensions to enhance transduction efficiency in MRC5 (normal fetal HLFs) and primary IPF cells, respectively, followed by puromycin selection (0.8 μg/mL) for 48–72 hours. Conditional overexpression or CRISPR/Cas9-mediated deletion of the aforementioned genes was achieved by addition of doxycycline (1 μg/mL, Cayman Chemicals) to conditioned medium.

### Statistics.

Statistical analysis was performed using GraphPad Prism, version 9.1.0 (GraphPad Software). Experimental data are presented as the mean and were analyzed for statistical significance by 1-way ANOVA with Tukey’s multiple-comparison test or paired/unpaired 2-tailed *t* test, as appropriate. A *P* values of less than 0.05 was considered significant. Data represent the mean ± SEM.

### Study approval.

All animal experiments were carried out with the approval of the University of Michigan IACUC and conformed to the Animal Research: Reporting of In Vivo Experiments (ARRIVE) guidelines.

### Data availability.

Publicly available RNA-Seq data were obtained from https://cellxgene.cziscience.com/ and http://www.ipfcellatlas.com/ Accession numbers: European Genome-phenome Archive (EGA), EGAS00001004344 (used to construct Azimuth lung v1 in Supplemental Figure 1A); Gene Expression Omnibus (GEO) GSE149563 (used to construct Supplemental Figure 2A); and IPF Atlas, GSE136831 and GSEW135893 (Supplemental Figure 2, C and D). Values and statistics for all data points in graphs can be found in the supplemental Supporting Data Values file.

## Author contributions

SMF, NMW, LRP, and MPG designed the in vitro and in vivo experiments. Experiments were performed by SMF, NMW, JMS, JDB, RLZ, and QS. Data were analyzed by SMF and NMW. Transgenic mice and intellectual contributions were provided by AMB. Patient-derived normal and IPF fibroblasts were provided by SKH. The manuscript was written by SMF and MPG. All authors reviewed the manuscript.

## Supplementary Material

Supplemental data

Unedited blot and gel images

Supporting data values

## Version 1 03/21/2024

In-Press Preview

## Version 2 05/15/2024

Electronic publication
