# Supplementary material for: MAPK phosphatase 1 inhibition of p38α within lung myofibroblasts is essential for spontaneous fibrosis resolution
Source: J Clin Invest. 2024 Mar 21;134(10):e172826. doi: 10.1172/JCI172826 (PMC11093610; doi:10.1172/JCI172826)
Supplement: Supplemental data [file jci-134-172826-s118.pdf]

MAP kinase phosphatase-1 inhibition of p38α within lung myofibroblasts is essential for spontaneous fibrosis resolution

Sean M. Fortier, Natalie Walker, Loka R. Penke, Jared Baas, Qinxue Shen, Jennifer Speth, Steven K. Huang, Rachel L. Zemans, Anton M. Bennett, Marc Peters-Golden

Supplemental Methods, Figures, and Tables

Supplemental Methods

Reagents. Unless otherwise specified, pharmacologic agents were reconstituted in DMSO as stock solutions and stored at –80°C with working concentrations indicated in parentheses. Recombinant human TGFβ (2 ng/mL for human fibroblasts, 5 ng/mL for mouse fibroblasts) was purchased from R&D (7754-BH) and resuspended in filter-sterilized 1% BSA. The non-specific p38 inhibitor SB203580 (20 μM) and p38α inhibitor VX-702 (50 μM) were purchased from Cayman Chemicals (13067) and Selleckchem (HY-10401), respectively, and stored at –20°C. PGE_2 (1 μM to human fibroblasts, 250 nM to mouse fibroblasts), the direct adenylyl cyclase activator forskolin (20 μM), and antifibrotic drugs pirfenidone (1 mM) and nintedanib (2 μM) were purchased from Cayman Chemicals (14010, 11018, 13986, and 11022). The PKA-selective cAMP agonist 6-BNZ cAMP (2 mM) and the Epac-selective agonist 8-pCPT cAMP (2 mM) were purchased from BioLog (B009 and C041) and reconstituted in sterile water. Fast and Power SYBR Green Master Mix and StepOne real-time PCR system were procured from Applied Biosystems.

Cell culture. CCL210 normal adult and MRC5 normal fetal HLFs were obtained from the American Type Culture Collection. MRC5 HLFs were utilized for all gain- and loss-of-function studies while CCL210s were used for all other in vitro studies involving normal HLFs unless otherwise specified. Primary IPF fibroblast lines and normal patient-derived HLFs were obtained from our institutional biorepository as described previously (1). All cells were initially cultured in low glucose DMEM (Invitrogen) supplemented with 10% FBS (Hyclone), 100 units/mL penicillin, and 100 μg/mL streptomycin (both from Invitrogen). Cells were then serum starved in FBS-free DMEM overnight, and differentiation to MFs was induced by treatment with TGFβ for 48 h. TGFβ–elicited MFs were then treated for specified time points as described followed by harvesting. Fibroblasts from lung tissue of saline- or bleomycin-treated mouse lungs were obtained during lung harvest at the indicated times as

previously described (2). For all in vitro experiments, a minimum of three independent experiments were performed, and the results are presented as mean ± SEM.

Western Blot. Cells were lysed in RIPA buffer supplemented with protease inhibitors (Roche Diagnostics, 11836153001) and a phosphatase inhibitor cocktail (EMD Biosciences, 524624 and 524625). Proteins were separated by SDS-PAGE and transferred to a nitrocellulose or polyvinylidene difluoride membrane. Membranes were subsequently blocked with 5% BSA and probed with a mouse antibody specific to αSMA (Agilent, M0851), CTHRC1 (Proteintech 16534-1-AP), α-tubulin (Millipore, T5168) or the rabbit antibodies targeting mouse MKP1 (Santa Cruz, 37384), human MKP1 (Cell Signaling Technologies; CST, 48625), collagen 1A1 (CST, 91144), fibronectin I (CST, 26836), total p38 (CST, 9212), phospho-p38 (CST, 9211), p38α (CST, 9218), p38ɣ (CST, 2307), total JNK (CST, 9252), phospho-JNK (CST, 9251), total ERK (CST, 9102), phospho-ERK (CST, 4370), and GAPDH HRP conjugate (CST, 8884). For Western blots involving MAPKs (p38, ERK, JNK), membranes were probed for the phosphorylated MAPK followed by membrane stripping and re-probing for its corresponding total MAPK which served as a loading control. For CRISPR/Cas9 studies in which p38α or p38γ were quantified, membranes were probed for each p38 isotype, stripped, and re-probed for GAPDH as loading control. α-tubulin served as a loading control and was directly probed on the same membrane for all Western blots involving MKP1, and in studies involving CRISPR/Cas9 deletion of p38 isoforms in which total p38 was measured. GAPDH was used as a loading control for all remaining Western blots. Proteins of interest grouped together with a loading control were run on the same gel.

qPCR. Analysis of transcript expression was performed by extracting total cellular RNA using an RNeasy kit (Qiagen). cDNA was prepared using the High-Capacity cDNA Reverse Transcription Kit (Applied Biosystems), amplified with Fast SYBR Green Master Mix, and analyzed on a StepOne real

time PCR system (Applied Biosystems). Fold changes were normalized to the expression levels of the housekeeping gene GAPDH. Primer pair sequences used for qPCR are listed in Supplemental Table 1.

Apoptosis assays. Apoptosis was determined either by measuring caspase 3/7 activity, extracellular Annexin V expression, or by TUNEL staining of fixed lung sections (Roche, 11684795910). For caspase 3/7 and Annexin V readouts, fibroblasts or MFs were treated with 100 ng/mL of anti-Fas activating antibody (EMD Biosciences, CH11 05-201) overnight to induce apoptosis. Caspase 3/7 activity was measured using the luciferase Caspase-Glo 3/7 kit (Promega, G8091) per the manufacturer's protocol (3). Annexin V+ cells were quantified using flow cytometry (BD Fortessa flow cytometer). For MKP1 overexpression in which Annexin V was quantified by flow cytometry, a vehicle control was used in lieu of GFP overexpression to avoid fluorescence interference. Briefly, fibroblasts or MFs were treated with the indicated therapies followed by exposure to anti-Fas (100 ng/mL) for 24 h. Cells were then lifted with 0.25% trypsin, pelleted in FACS tubes, and stained with AlexaFluor 488 conjugated Annexin V antibody (Invitrogen, V13241) per the manufacturer's protocol (4). TUNEL staining of mouse lung tissue was achieved using 10 µg/mL proteinase K digestion at 37°C for 30 minutes followed by signal development per the manufacturer's protocol.

Immunofluorescence microscopy and immunohistochemistry. CCL210 or MRC5 fibroblasts were plated and cultured in single-chamber slides and serum starved overnight. Fibroblast differentiation into MFs was achieved by addition of TGFβ at 2 ng/mL for 48 h and treatment with either vehicle or the indicated therapies to elicit MF dedifferentiation. Chamber slides were then washed twice with chilled PBS, fixed with freshly prepared 4% formaldehyde for 20 min, washed with PBS, and quenched with 100 mM glycine for 15 min. Blocking and permeabilization were achieved by incubating the slides for 1 h in PBS containing 10% FBS and 0.1% Triton X-100 (Sigma-Aldrich).

Mouse lungs were fixed with 10% formalin, embedded in paraffin, sectioned, and mounted on glass slides. Subsequent deparaffinization, antigen retrieval blocking and staining was performed as previously described (5). Staining of fixed fibroblasts and murine lung tissue was performed with the following primary antibodies: anti–αSMA-FITC (1:500; F3777, Sigma-Aldrich) or anti-αSMA–Cy3 (1:500; Millipore, C6198) overnight at 4°C. MKP1 (1:100, Millipore 07-535), rabbit anti-p-p38 (1:500; CST 4511), CD68 (1:500; Abcam ab125212), E-cadherin (1:100; BD Biosciences 610181), proSPC (1:500; Millipore AB3786), PDPN (1:100; University of Iowa DSHB 8.1.1). Unconjugated primary antibodies were imaged via fluorescent secondary antibodies: Cy3 (1:250; Jackson 711-166-152) and Cy5 (1:250; Jackson 107-605-142) with the exception of MKP1 which was imaged using Cy5 tyramide (AKOYA, TS-000103) after citrate retrieval per the manufacturer's protocol. Mounting medium containing DAPI (Invitrogen) was used to stain nuclei. Images were obtained using a Nikon Eclipse Ti2 Inverted Confocal or BX53 (Olympus) microscopes. Whole slide trichrome images were obtained using a Vectra Polaris Brightfield Scanner.

References

1. Scruggs AM, Grabauskas G, Huang SK. The Role of KCNMB1 and BK Channels in Myofibroblast Differentiation and Pulmonary Fibrosis. Am J Respir Cell Mol Biol 2020; 62: 191-203.

2. Penke LRK, Speth JM, Huang SK, Fortier SM, Baas J, Peters-Golden M. KLF4 is a therapeutically tractable brake on fibroblast activation which promotes resolution of pulmonary fibrosis. JCI Insight 2022.

3. Penke LRK, Speth J, Wettlaufer S, Draijer C, Peters-Golden M. Bortezomib Inhibits Lung Fibrosis and Fibroblast Activation Without Proteasome Inhibition. Am J Respir Cell Mol Biol 2021.

4. Devries-Seimon T, Li Y, Yao PM, Stone E, Wang Y, Davis RJ, Flavell R, Tabas I. Cholesterol-induced macrophage apoptosis requires ER stress pathways and engagement of the type A scavenger receptor. J Cell Biol 2005; 171: 61-73.

5. Wang F, Ting C, Riemondy KA, Douglas M, Foster K, Patel N, Kaku N, Linsalata A, Nemzek J, Varisco BM, Cohen E, Wilson JA, Riches DW, Redente EF, Toivola DM, Zhou X, Moore BB, Coulombe PA, Omary MB, Zemans RL. Regulation of epithelial transitional states in murine and human pulmonary fibrosis. J Clin Invest 2023; 133.

Supplemental Figures and Tables

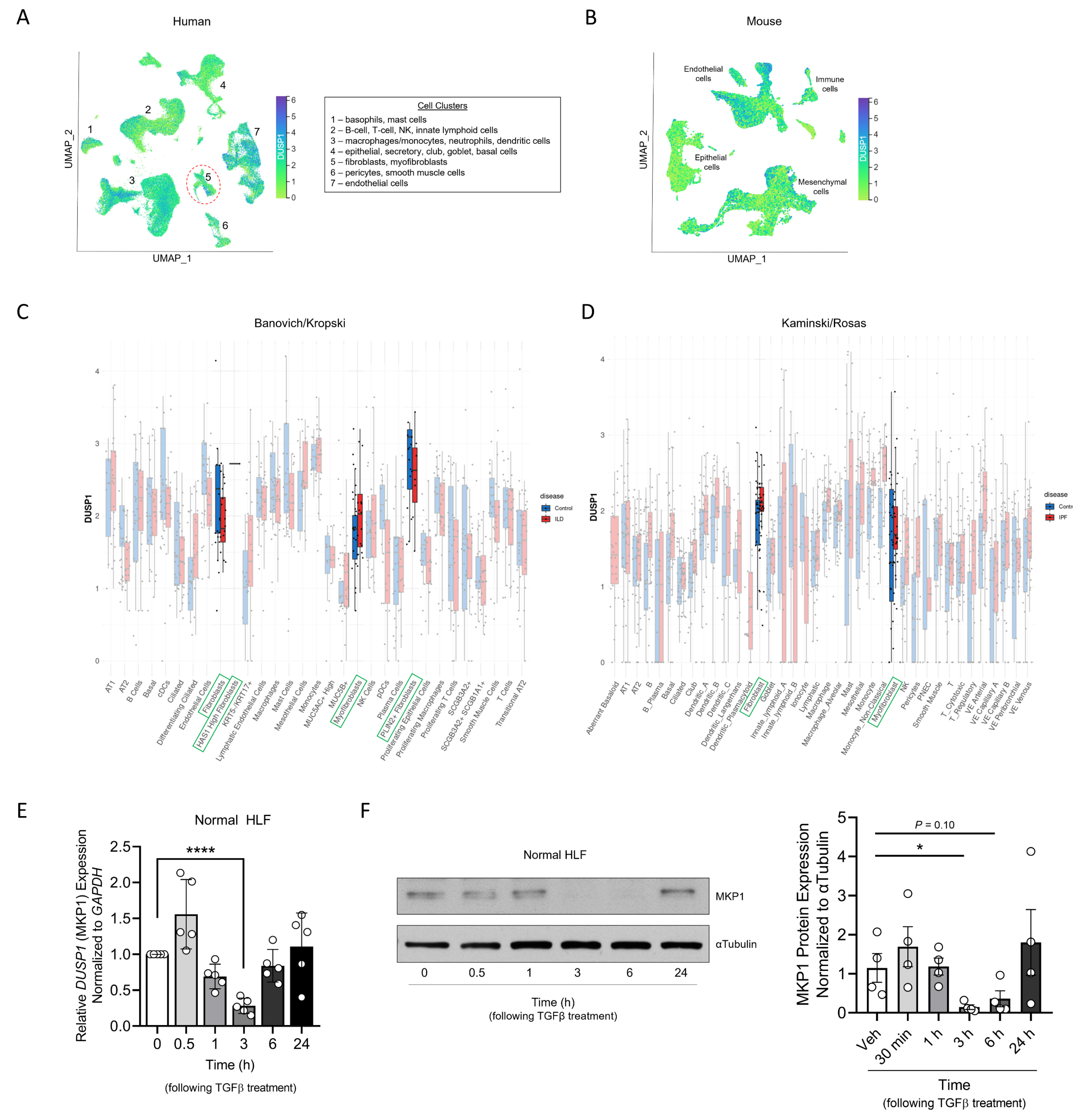

Supplemental Figure 1: (**A** and **B**) UMAP plots of human (**A**) and mouse (**B**) lung cells generated via the Chan Zuckerberg Initiative online database (https://cellxgene.cziscience.com/). Relative MKP1 transcript (*DUSP1*) levels in each plot are indicated by the color map legend. Human and mouse lung cell types are numbered (identified in the legend, right) and directly labeled, respectively. Fibroblast and MF subpopulations in (**A**) are encircled by the dashed red oval. (**C** and **D**) Relative expression of human *DUSP1* (MKP1) transcript in various lung cells from normal versus IPF patients (**C**) and from normal patients versus patients with various ILDs (**D**) derived from publicly available single cell RNA sequencing data (http://www.ipfcellatlas.com/). Fibroblast subtypes are outlined in green. (**E**) Kinetics of *DUSP1* (MKP1) transcript expression in patient-derived normal HLFs following TGFβ (2 ng/mL) treatment. (**F**) Kinetics of MKP1 protein expression in patient-derived normal HLFs following TGFβ (2 ng/mL) treatment. Each data point represents a distinct experiment and patient-derived cell line. Significance for qPCR in **E** (n = 5) and densitometry in **F** (n = 4) was determined by 2-tailed t-test. * $P<0.05$, **** $P<0.0001$.

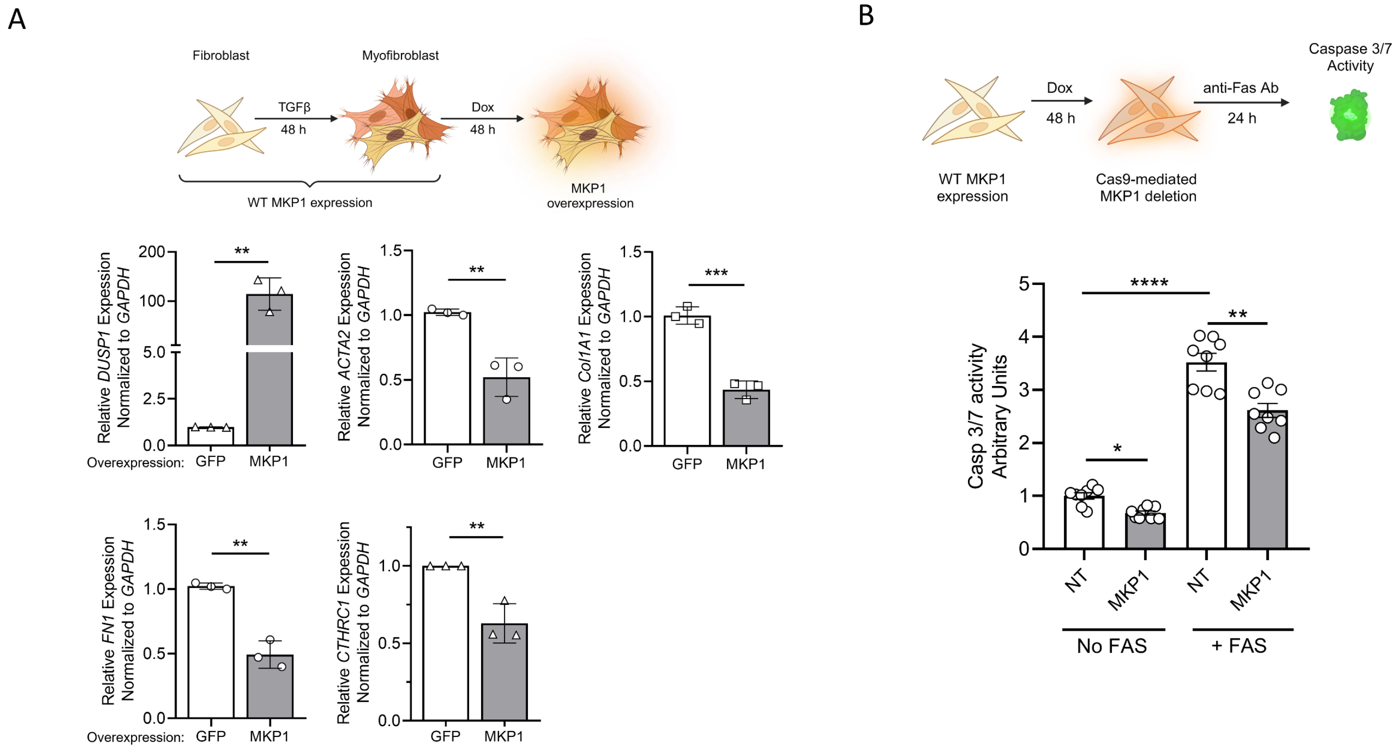

Supplemental Figure 2: Inducible MKP1 overexpression in human lung MFs (protocol schematic in **A**, top). (**A**) Graphs represent relative transcript levels of *DUSP1* (MKP1), *ACTA2*, *COL1A1*, *FN1*, and *CTHRC1* determined by qPCR (bottom). Normal HLFs containing inducible CRISPR/Cas9 non-targeting (NT) or MKP1 sgRNAs used in (**A**), were treated with doxycycline followed by anti-Fas antibody (100 ng/mL) for 24 h (protocol schematic used in **B**, top). (**B**) Apoptosis was determined by caspase 3/7 activity assay. Each data point represents an individual experiment. Significance for qPCR and densitometry was determined by 2-tailed t-test in **A** (n = 3) and by 1-way ANOVA in **B** (n = 8). * *P*<0.05, ***P*<0.01, *** *P*<0.001, **** *P*<0.0001.

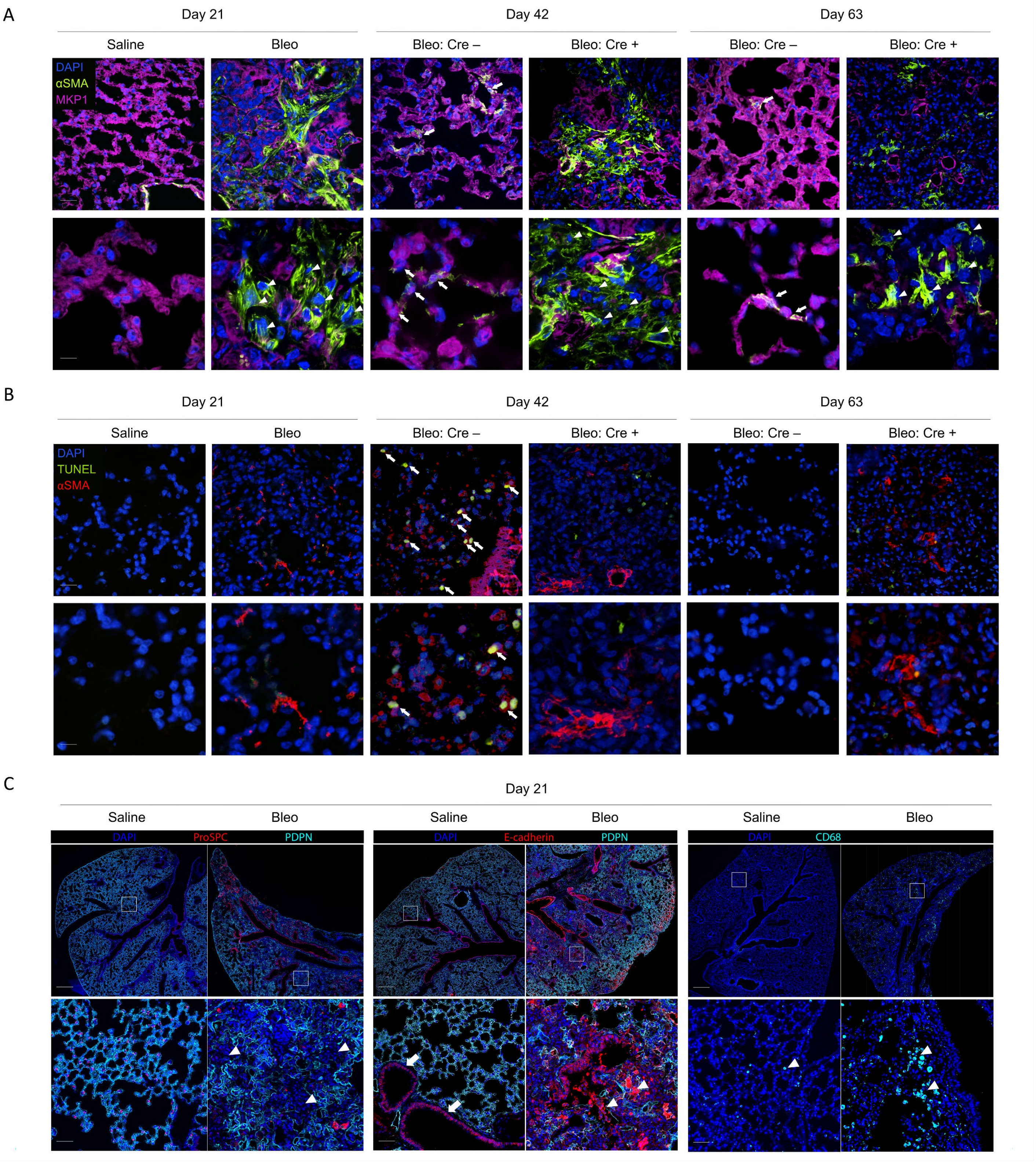

A
Day 21
Day 42
Day 63
Saline
Bleo
Bleo: Cre –
Bleo: Cre +
Bleo: Cre –
Bleo: Cre +
DAPI
αSMA
MKP1
B
Day 21
Day 42
Day 63
Saline
Bleo
Bleo: Cre –
Bleo: Cre +
Bleo: Cre –
Bleo: Cre +
DAPI
TUNEL
αSMA
C
Day 21
Saline
Bleo
Saline
Bleo
Saline
Bleo
DAPI
ProSPC
PDPN
DAPI
E-cadherin
PDPN
DAPI
CD68

Supplemental Figure 3: (**A**) Immunofluorescence microscopy of MKP1 (Cy5 tyramide) and αSMA (FITC) in fixed lung sections from mice harvested 21, 42, or 63 days following oropharyngeal (o.p.) saline or bleomycin administration. Nuclei are stained with DAPI. White arrowheads: MFs lacking Cy5 stain (αSMA+/MKP1–). White arrows: double positive cells (αSMA+/MKP1+). Scale bars: 20 μm (top row), 7.5 μm (bottom row). (**B**) TUNEL (FITC) and αSMA (Cy3) staining by immunofluorescence microscopy in fixed lung sections from mice harvested 21, 42, or 63 days following o.p. saline or bleomycin administration. White arrows: double positive cells (TUNEL+/αSMA+). Nuclei are stained with DAPI. Scale bars: 20 μm (top row), 10 μm (bottom row). (**C**) Immunofluorescence microscopy of mouse lung sections harvested 21 days after administration of bleomycin (or saline) displaying type I (PDPN) and type II (proSPC) cells (left), E-cadherin positive cells (middle), and alveolar macrophages (CD68, right). Scale bars: 500 μm (top row), 50 μm (bottom row). White arrows: normal airways. White arrowheads: alveolar regions devoid of type I cells (left), regions of parenchymal bronchiolization (middle), and alveolar macrophages (right).

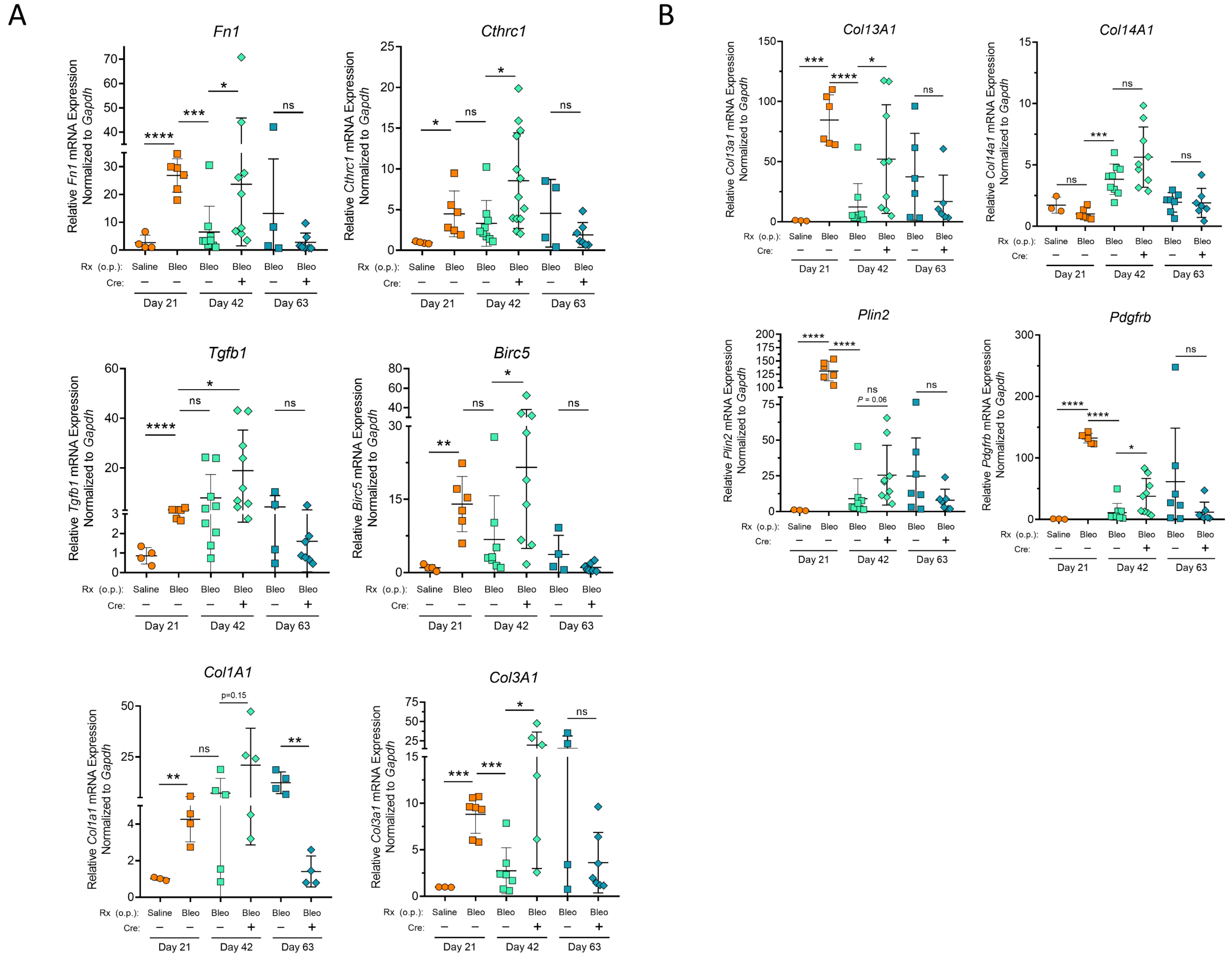

Supplemental Figure 4: (**A** and **B**) Quantification by qPCR from left and right upper/middle lobes harvested on days 21, 42, or 63 (same homogenates used to quantify hydroxyproline in Figure 3G). (**A**) Pathologic fibroblast/fibrosis-associated genes. (**B**) Genes defining specific fibroblast subsets. Each point represents an individual mouse. Significance was determined by unpaired 2-tailed t-test. * *P*<0.05, ** *P*<0.01, *** *P*<0.001, **** *P*<0.0001.

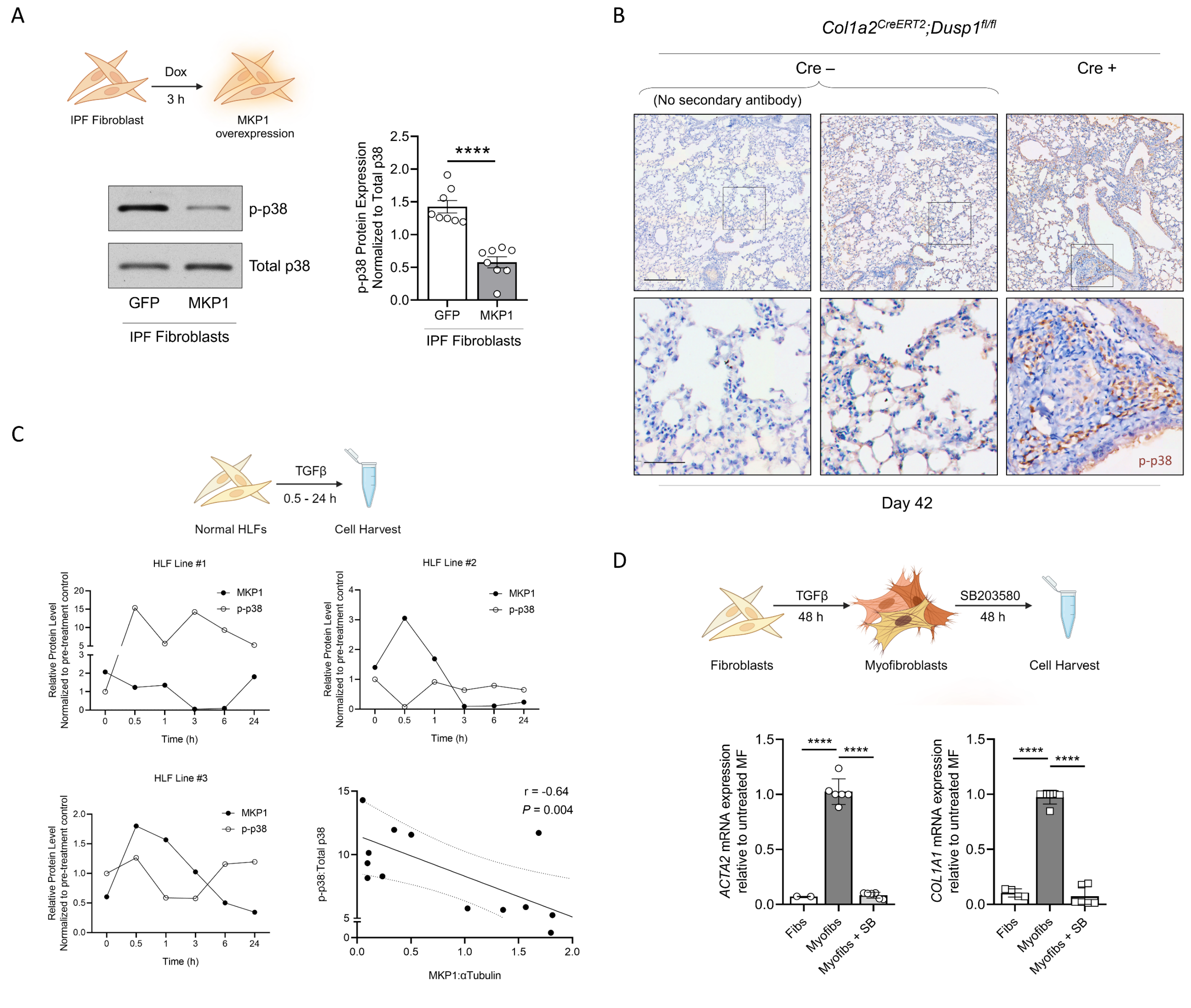
A
Dox
3 h
IPF Fibroblast
MKP1 overexpression
p-p38
Total p38
GFP
MKP1
IPF Fibroblasts
p-p38 Protein Expression Normalized to Total p38
****
GFP
MKP1
IPF Fibroblasts
B
Col1a2CreERT2;Dusp1fl/fl
Cre –
Cre +
(No secondary antibody)
p-p38
Day 42
C
TGFβ
0.5 - 24 h
Normal HLFs
Cell Harvest
HLF Line #1
HLF Line #2
HLF Line #3
MKP1
p-p38
Relative Protein Level Normalized to pre-treatment control
Time (h)
r = -0.64
P = 0.004
p-p38:Total p38
MKP1:αTubulin
D
TGFβ
48 h
SB203580
48 h
Fibroblasts
Myofibroblasts
Cell Harvest
ACTA2 mRNA expression relative to untreated MF
COL1A1 mRNA expression relative to untreated MF
****
Fibs
Myofibs
Myofibs + SB

Supplemental Figure 5: (**A**) Inducible MKP1 overexpression in IPF fibroblasts followed by treatment with doxycycline for 3 h (protocol schematic in **A**, top left). Cells were then harvested and protein quantification of p-p38 was performed by Western blot. (**B**) Immunohistochemistry staining using p-p38 primary antibody within lung sections from Cre negative and positive mouse lungs harvested on day 42. Black squares: regions imaged in high power. Brown: DAB, Blue: hematoxylin. Scale bars: 500 μm (top row), 100 μm (bottom row). (**C**) Normal patient-derived HLFs were treated with TGFβ (2 ng/mL) for 0.5, 1, 3, 6, or 24 h (protocol schematic in **C**, top). Time course kinetics of MKP1 and p-p38 protein levels quantified by Western blot in normal patient-derived HLFs after treatment with TGFβ and linear regression analysis of MKP1 levels compared to p-p38 levels (bottom, right). The correlation coefficient "r" was determined using a Pearson correlation test. (**D**) Normal HLFs were treated with TGFβ (2 ng/mL) for 48 h to generate MFs followed by treatment with or without the p38 inhibitor SB203580 (20 μM) for 48 h (protocol schematic in **D**, top). Each graph represents relative transcript levels of *ACTA2* (left), and *COL1A1* (right) determined by qPCR. Each data point represents a distinct experiment. Significance was determined by unpaired 2-tailed t-test in **A** (n = 8) and 1-way ANOVA in **D** (n = 6). **** $P<0.0001$.

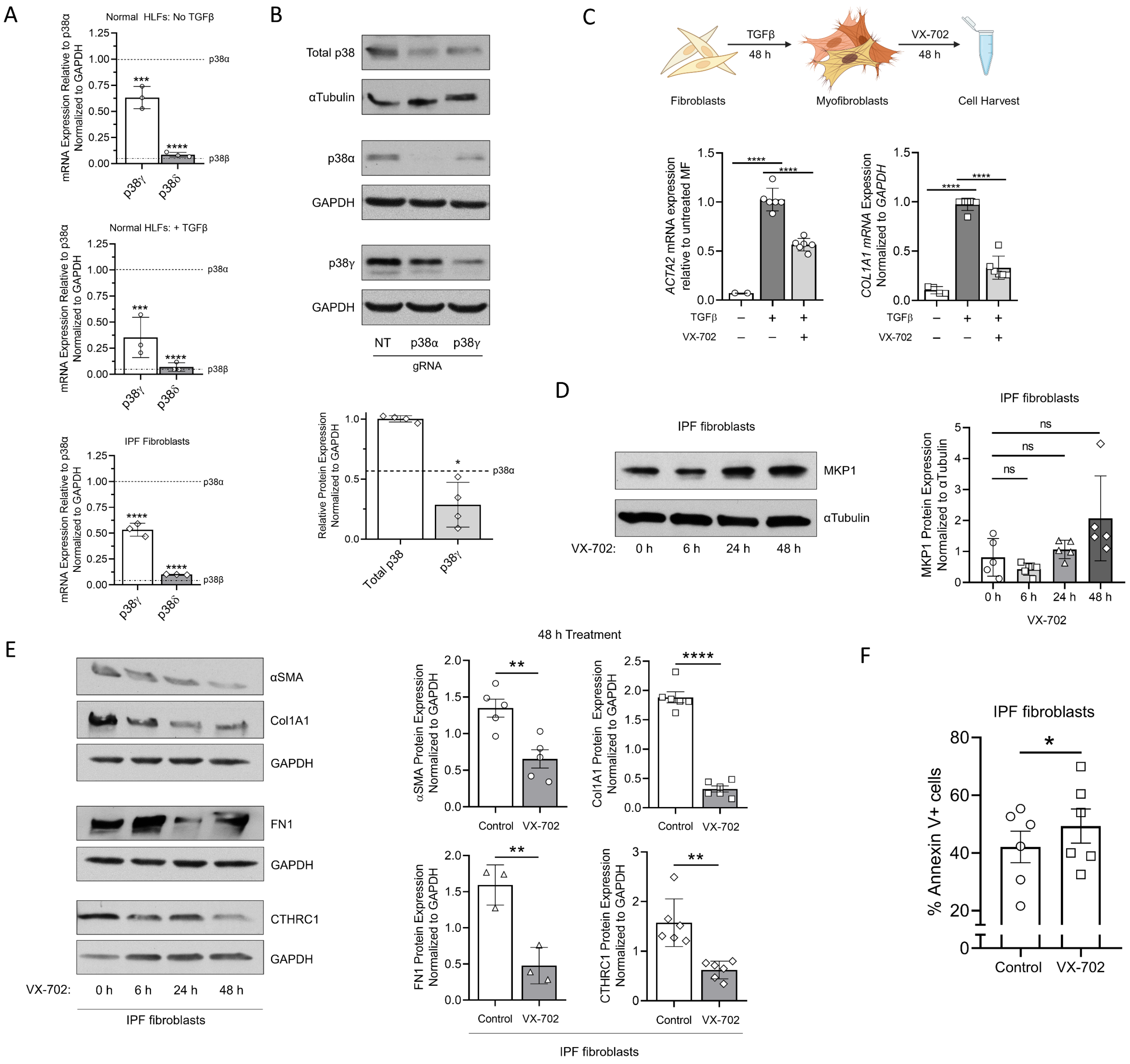
A
Normal HLFs: No TGFβ
mRNA Expression Relative to p38α Normalized to GAPDH
p38α
p38β
p38γ
p38δ
Normal HLFs: + TGFβ
IPF Fibroblasts
B
Total p38
αTubulin
p38α
GAPDH
p38γ
NT
gRNA
Relative Protein Expression Normalized to GAPDH
Total p38
C
TGFβ
48 h
VX-702
Fibroblasts
Myofibroblasts
Cell Harvest
ACTA2 mRNA expression relative to untreated MF
COL1A1 mRNA Expression Normalized to GAPDH
D
IPF fibroblasts
MKP1
VX-702: 0 h 6 h 24 h 48 h
MKP1 Protein Expression Normalized to αTubulin
ns
E
αSMA
Col1A1
FN1
CTHRC1
48 h Treatment
αSMA Protein Expression Normalized to GAPDH
Col1A1 Protein Expression Normalized to GAPDH
FN1 Protein Expression Normalized to GAPDH
CTHRC1 Protein Expression Normalized to GAPDH
Control
F
% Annexin V+ cells

Supplemental Figure 6: (**A**) Relative expression of p38ɣ (*MAPK12*) and p38δ (*MAPK13*) transcripts determined by qPCR from normal untreated HLFs (top), TGFβ-treated HLFs for 48 h (middle), or patient-derived IPF lung fibroblasts (bottom). Dashed lines in each graph represent the relative levels of p38α or p38β (Figure 5A). (**B**) Protein quantification of p38ɣ by Western blot (top) in normal HLFs following Cas9-mediated deletion of p38α, p38ɣ, or use of a non-targeting (NT) sgRNA control. In the graph depicting the relative expression of p38ɣ (bottom), the value was derived by subtracting the densitometric value of the total p38 band in the isoform deleted line from that of the total p38 band of the WT line and adjusting this value to account for incomplete isoform knockdown. The dashed line represents the relative protein expression of p38α (Figure 5B). (**C**) MF treatment with VX-702 (50 μM) (protocol schematic, top). Relative transcript levels of *ACTA2* (left) and *COL1A1* (right), determined by qPCR. (**D**) Protein quantification by Western blot of MKP1 in IPF fibroblasts treated with VX-702 for the indicated times. (**E**) Protein quantification by Western blot of αSMA, Col1A1, FN1, and CTHRC1 in IPF fibroblasts treated with VX-702 (left) densitometry (right). (**F**) Annexin V expression measured by flow cytometry in IPF fibroblasts treated with VX-702 (50 μM) followed by anti-Fas activating antibody (100 ng/mL) for 24 h. Each data point represents a distinct experiment or patient derived line. Each blot grouping containing a protein(s) of interest and its corresponding loading control was run on a separate gel. Significance was determined by unpaired 2-tailed t-test in **A** (n = 3), **D** (n = 5), **E** (n = 5), and **F** (n = 5) and by 1-way ANOVA in **B** (n = 4) and **C** (n = 6). * $P<0.05$, ** $P<0.01$, *** $P<0.001$, **** $P<0.0001$.

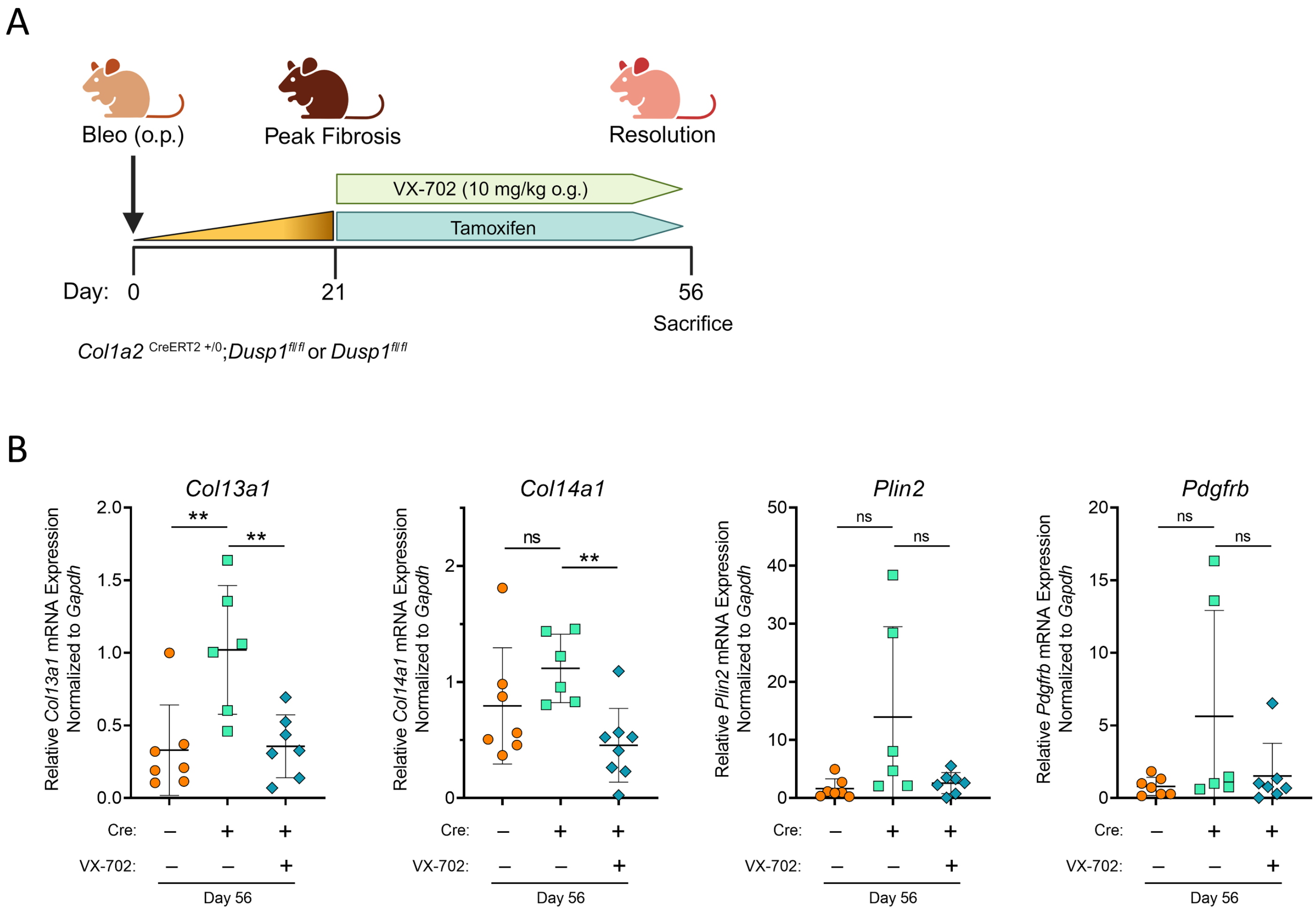

Supplemental Figure 7: (**A**) Schematic illustrating the “resolution protocol”. A tamoxfen chow diet was introduced and VX-702 was administered to Cre positive mice by oral gavage (o.g.) daily starting on day 21 until sacrifice on day 56. (**B**) Fibroblast subset gene quantification by qPCR of *Col13a1*, *Col14a1*, *Plin2*, and *Pdgfrb* from left and right upper/middle lobe lung homogenates in Cre negative, Cre positive, and Cre positive mice treated with VX-702 harvested on day 56 following bleomycin (1 U/kg). Each point represents an individual mouse. Significance for was determined by unpaired 2-tailed t-test. ** $P<0.01$.

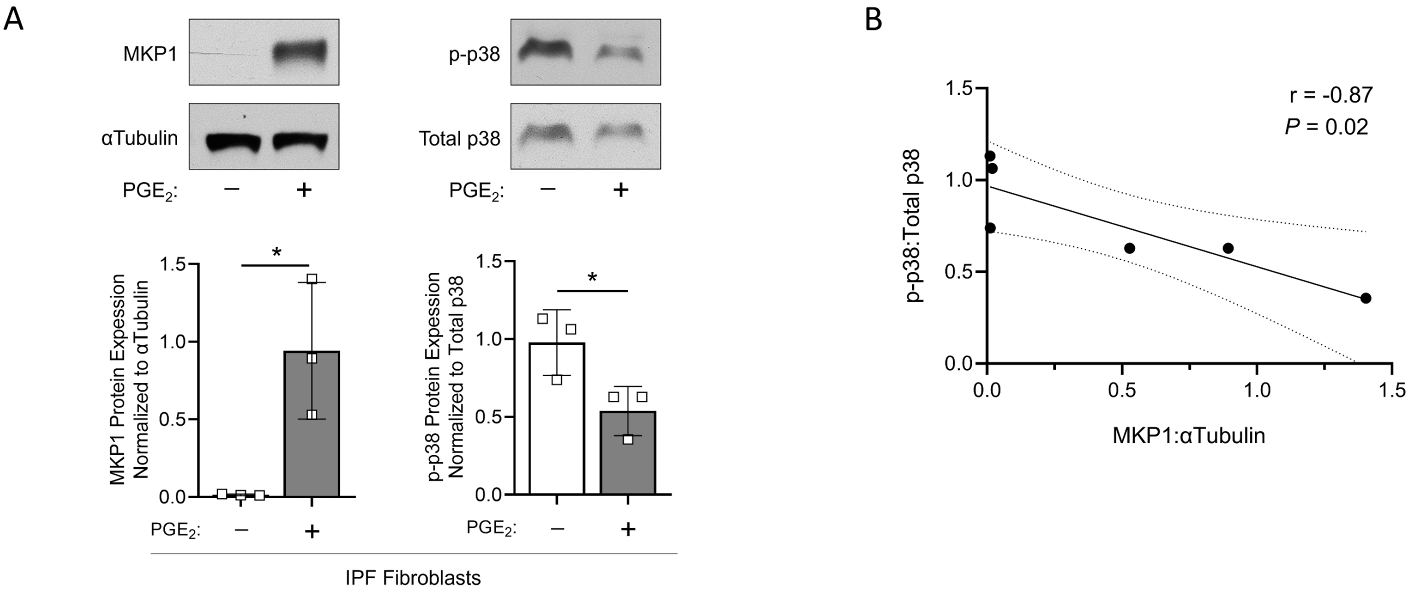

Supplemental Figure 8: (**A**) Protein quantification by Western blot of p-p38 and MKP1 within IPF fibroblasts treated with PGE_2 for 6 h. (**B**) Linear regression analysis of MKP1 levels compared to p-p38 levels from densitometric analysis in **A**. The correlation coefficient "r" was determined using a Pearson correlation test. For the blot in **A**, MKP1, p-p38, total p38, and GAPDH were run on separate gels in parallel. Each blot grouping containing a protein(s) of interest and its corresponding loading control was run on a separate gel. Significance in **A** (n = 3) was determined by unpaired 2-tailed t-test. * $P<0.05$.

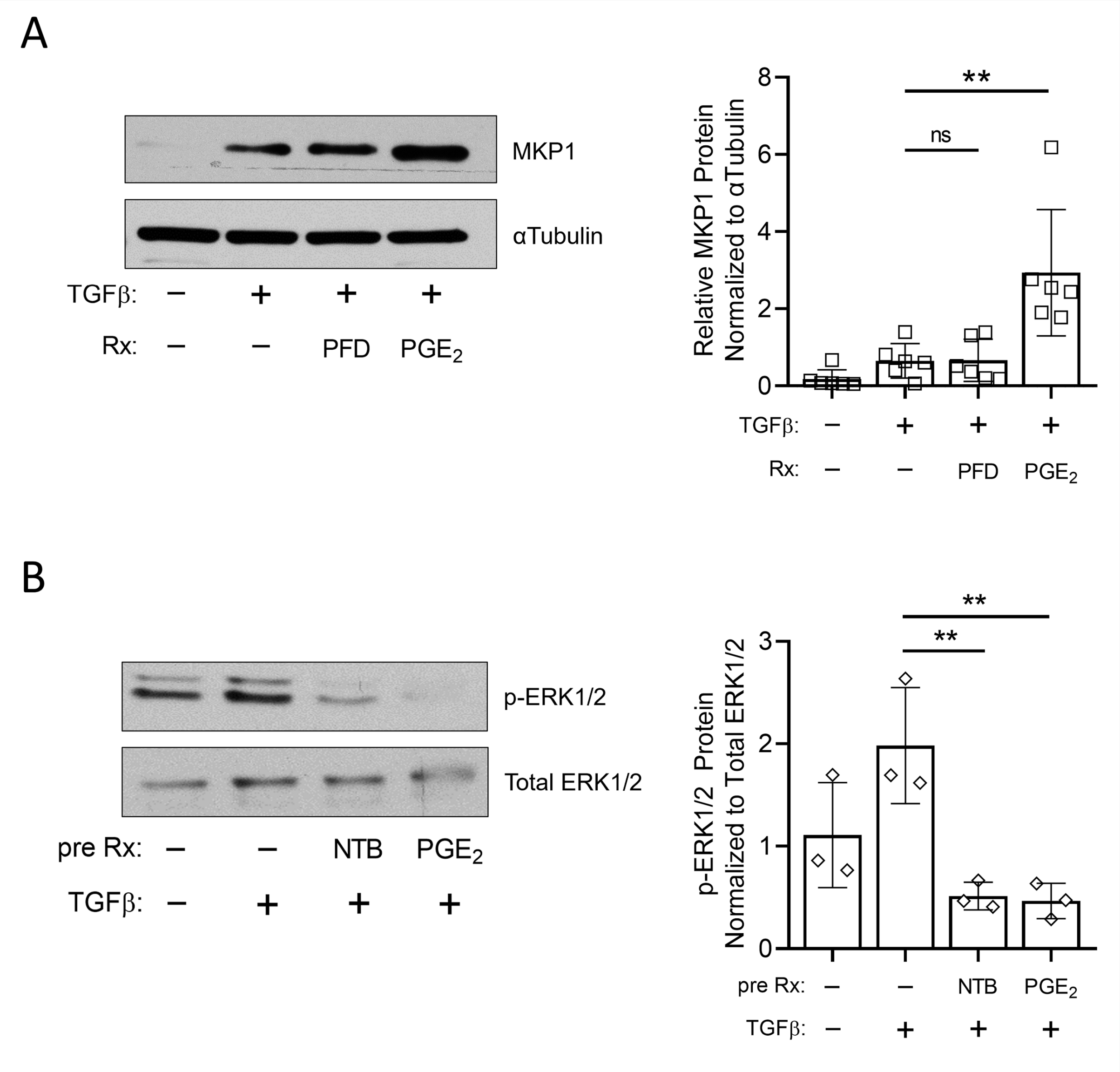

Supplemental Figure 9: (**A**) MKP1 protein quantification by Western blot and densitometry analysis of normal HLFs treated with vehicle and MFs (generated by treatment with TGFβ at 2 ng/mL for 48 h) treated with vehicle or pirfenidone (1 mM) or PGE_2 (1 μM) for 24 h. (**B**) Phosphorylated ERK1/2 quantification by Western blot and densitometry analysis of normal HLFs treated with nintedanib (2 μM) for 6 h. Significance in **A** (n = 6) and **B** (n = 3) was determined by 2-tailed t-test. ** *P*<0.01.

	Gene	Forward sequence (5' to 3')	Reverse sequence (5' to 3')
Human Primers	*GAPDH*	CAGCCTCAAGATCATCAGCA	ACAGTCTTCTGGGTGGCAGT
	DUSP1	TGCCTTGATCAACGTCTCAG	ACCCTTCCTCCAGCATTCTT
	ACTA2	ATCACCAACTGGGACGACAT	CATACATGGCTGGGACATTG
	COL1A1	CTGCTGGCAAGAGTGGTGAT	GGTGACCCTTTATGCCTCTG
	FN1	CACCACAGCCATCTCACATT	CTGGCCCTCGTATACCACAC
	CTHRC1	GCTCACTTCGGCTAAAATGC	CAGCACCAATTCCTTCACAA
	MAPK11	AGTGACCAGAGGGTCAGTGC	GGGCTTGAAGCTGAGGACT
	MAPK12	GGACCAGCTGAAGGAGATCA	TTCTCCAGGAGGTTCACAGC
	MAPK13	GATGACTGGCTACGTGGTGA	CTTTCAGGATCTGGGTCAGC
	MAPK14	TGCACATGCCTACTTTGCTC	CTTCTTGGTCAAGGGGTGGT
Mouse Primers	*Gapdh*	GTGTTTCCTCGTCCCGTAGA	GCCGTGAGTGGAGTCATACT
	Fn1	GAAGTCGCAAGGAAACAAGC	GTTGTAGGTGAACGGGAGGA
	Cthrc1	GACCAAGGAAGCCCTGAGTT	ACCCAGATGGCCACATCTAC
	Tgfb1	CAACAATTCCTGGCGTTACC	GCTGAATCGAAAGCCCTGTA
	Birc5	ATCGCCACCTTCAAGAACTG	GCTCCTCTATCGGGTTGTCA
	Col1a1	ACCTCAGGGTATTGCTGGAC	CACCACTTGATCCAGAAGGA
	Col3a1	GCACAGCAGTCCAACGTAGA	CGCAAAGGACAGATCCTGAG
	Acta2	TCCCTGGAGAAGAGCTACGA	GCTGACTCCATCCCAATGAA
	Col13a1	TCTGGGATTGCCTGGTACTC	TGTCCCCTTTAGGCCCTCTA
	Col14a1	TTGTGGATGACTTTGACGCC	ACCAGGCTCCATAGAAACCC
	Plin2	CTCTGCTCTCGGGCTTATCA	CACCCACGAGACATAGAGCT
	Pdgfrb	CCACATTCCTTGCCCTTCAA	CACAGGGTCCACGTAGATGT

Supplemental Table 1: Forward and reverse human and mouse primer sequences used to determine transcript levels of the indicated gene in qPCR experiments.
