# Supplementary material for: MAPK phosphatase 1 inhibition of p38α within lung myofibroblasts is essential for spontaneous fibrosis resolution
Source: J Clin Invest. 2024 Mar 21;134(10):e172826. doi: 10.1172/JCI172826 (PMC11093610; doi:10.1172/JCI172826)

Full unedited blots for Figure 1:

1B

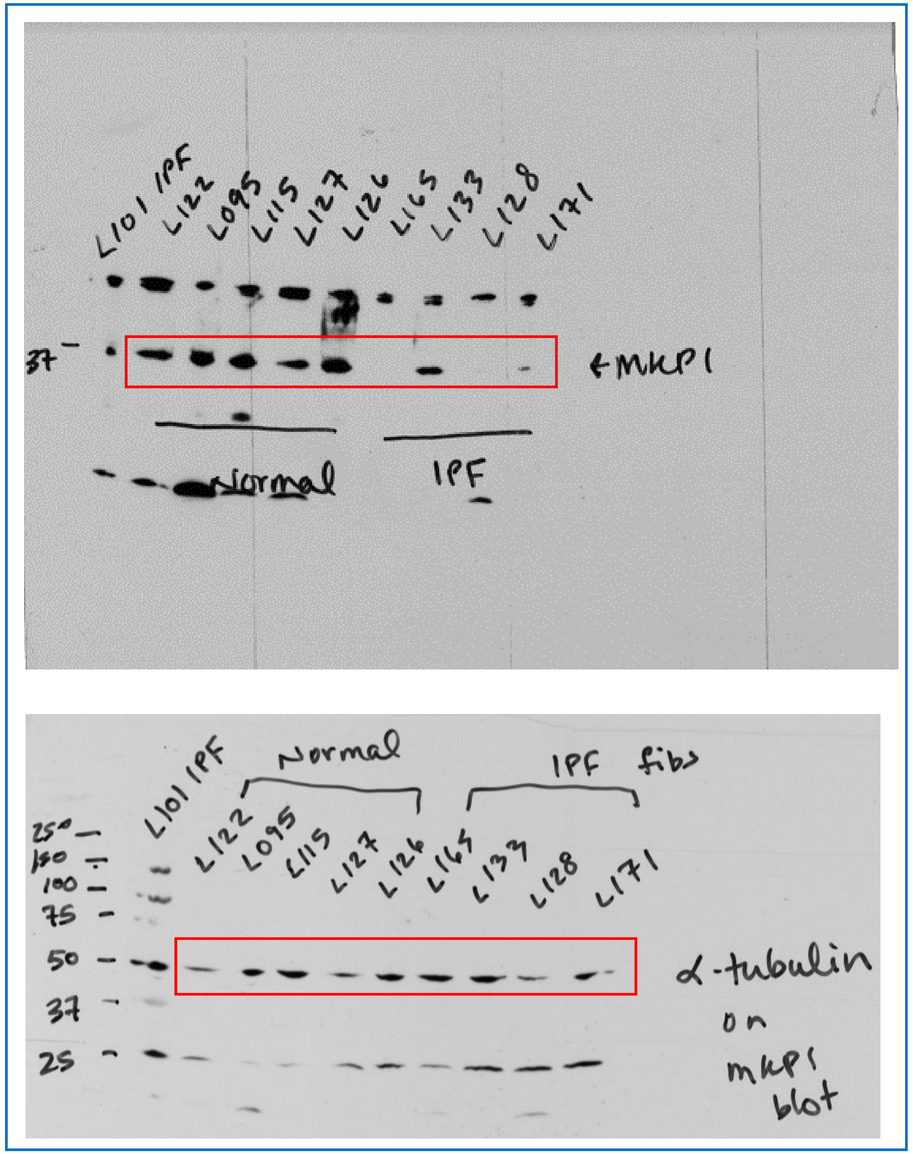

1C

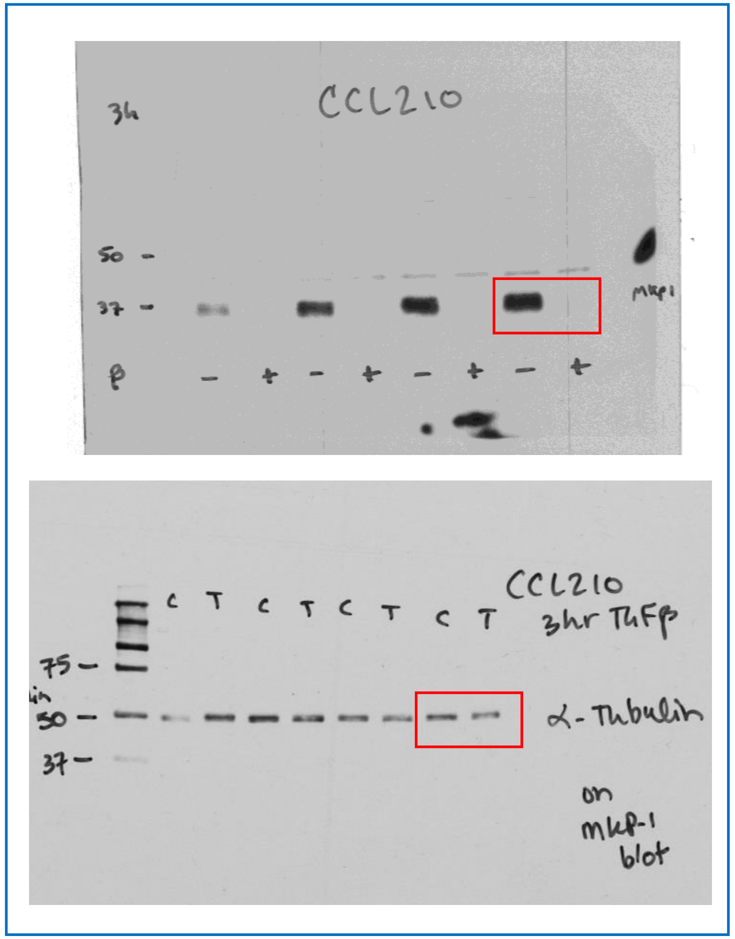

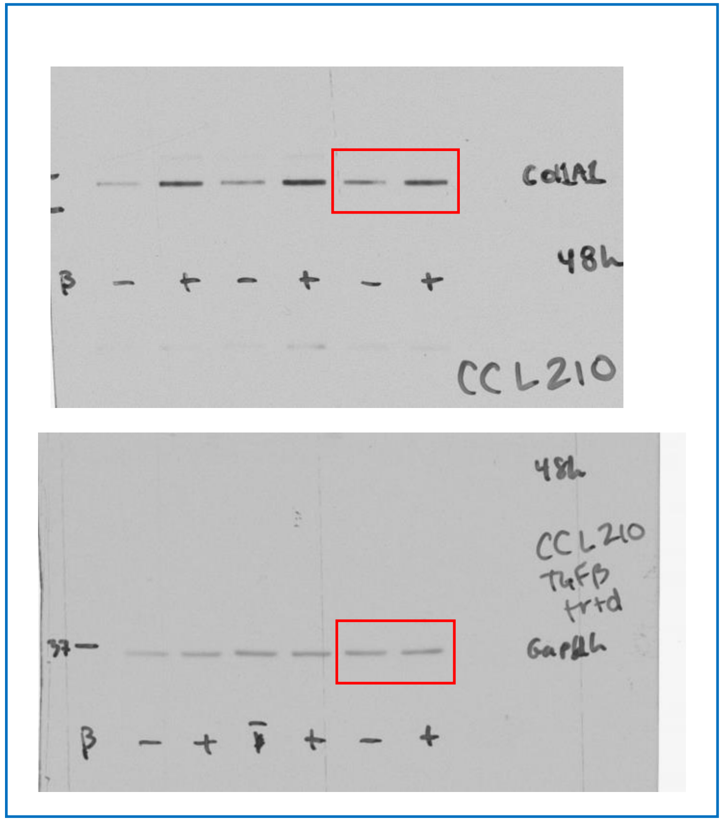

RIGHT: GAPDH (BOTTOM) was run in the same lanes on the same gel as the Col1a1 bands (TOP). The membrane was cut and developed separately.

Full unedited blots for Figure 2A: MKP1, Collagen, and SMA

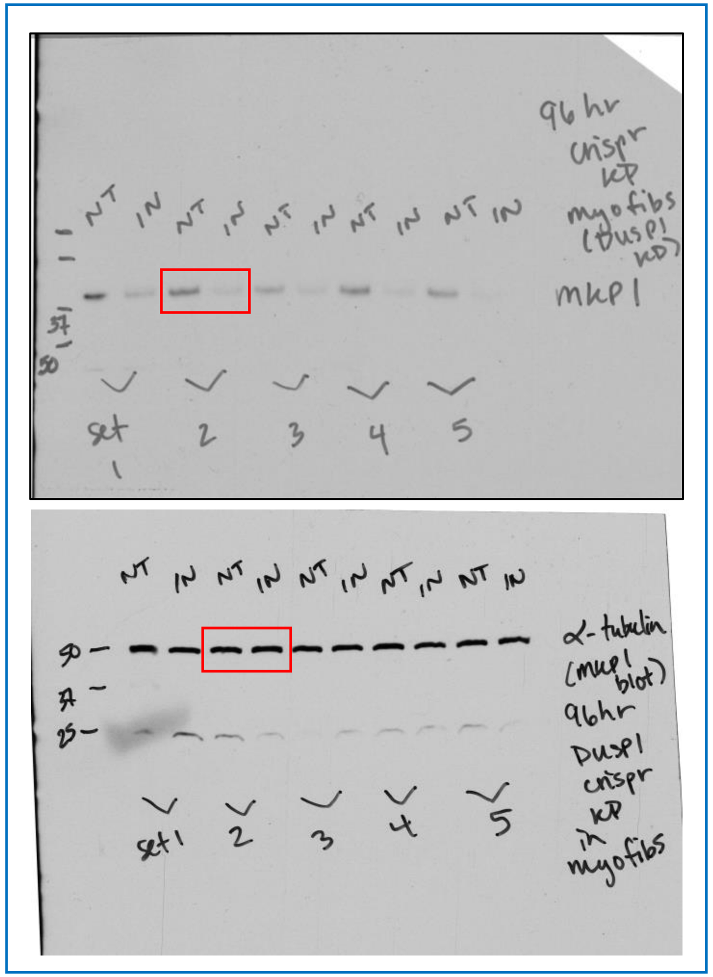

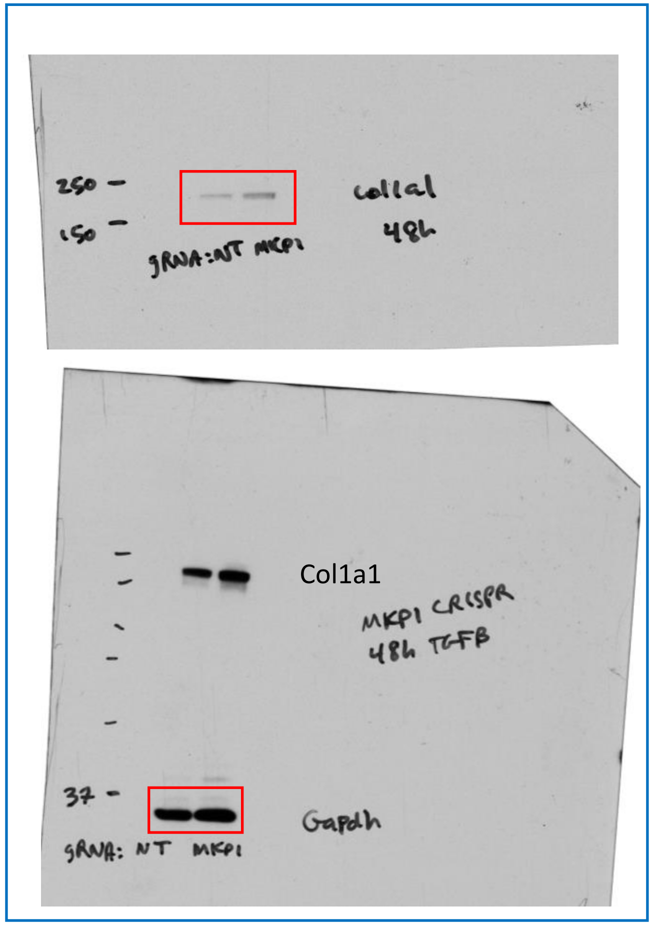

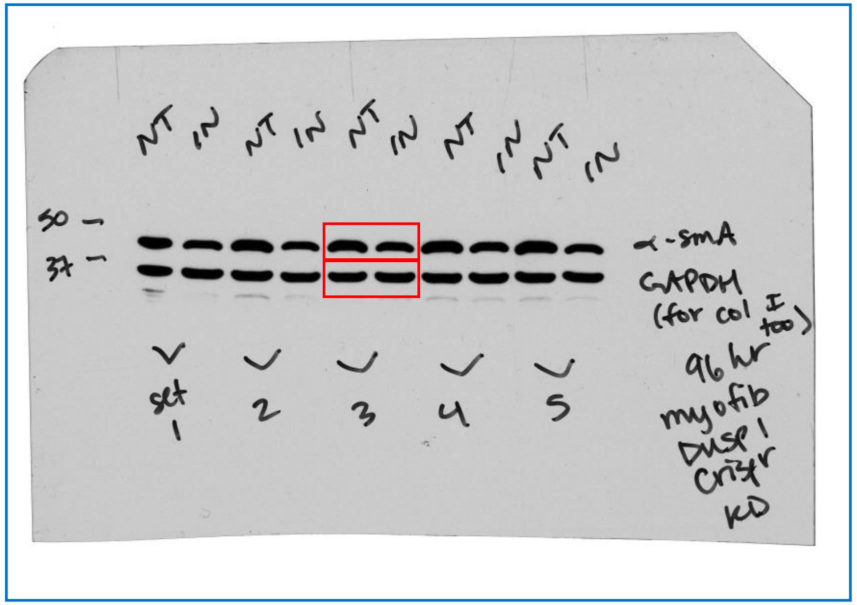

Full unedited blots for Figure 2A: FN1 and CTHRC1

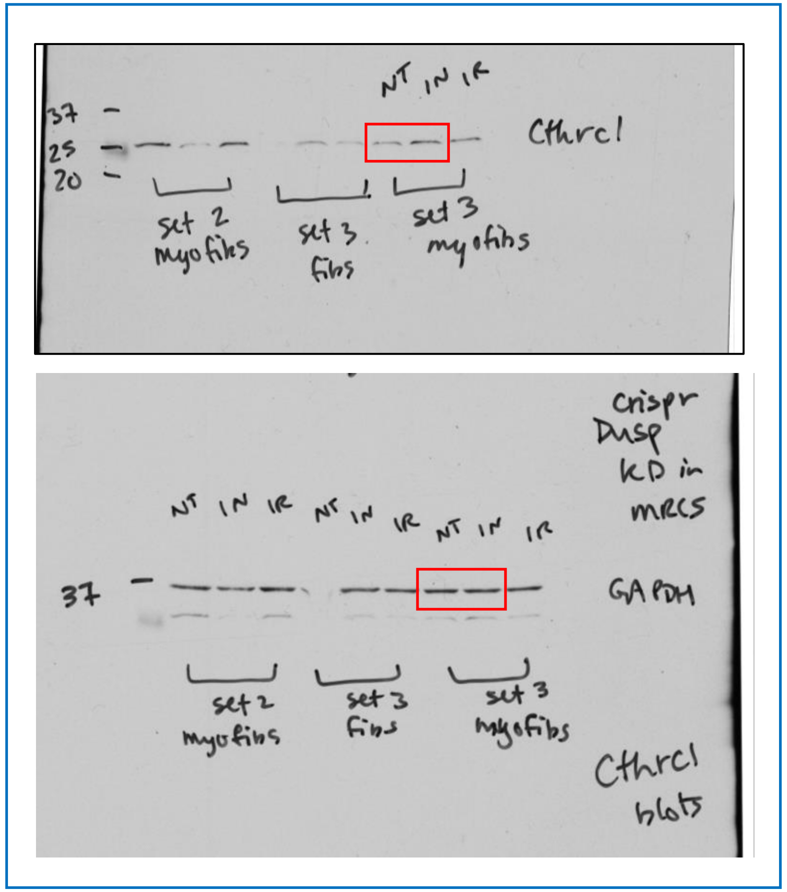

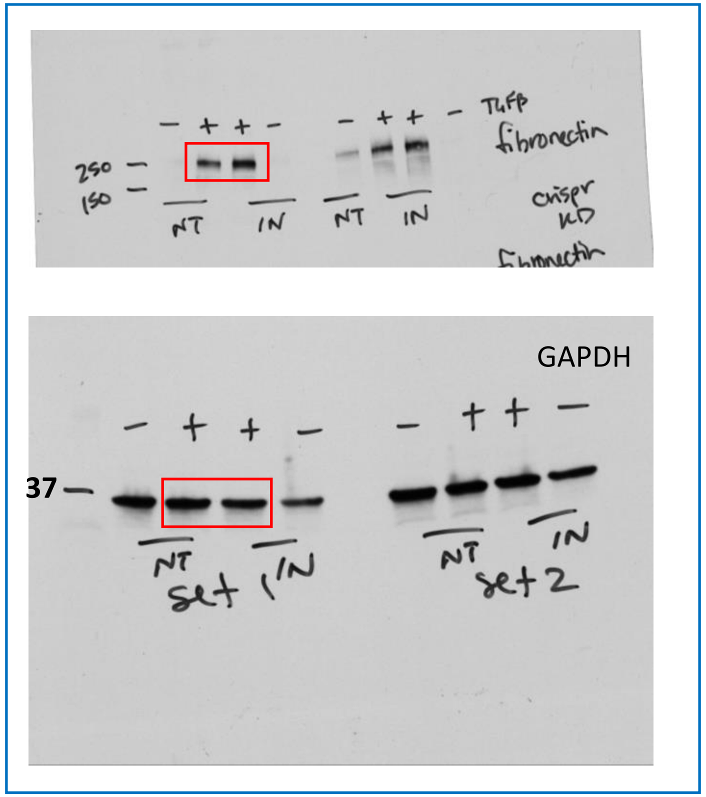

GAPDH from set 1 (BOTTOM) was run in the same lanes and on the same gel as the fibronectin bands (TOP LEFT). The membrane was cut and developed separately.

Full unedited blots for Figure 2B: MKP1, Collagen, SMA

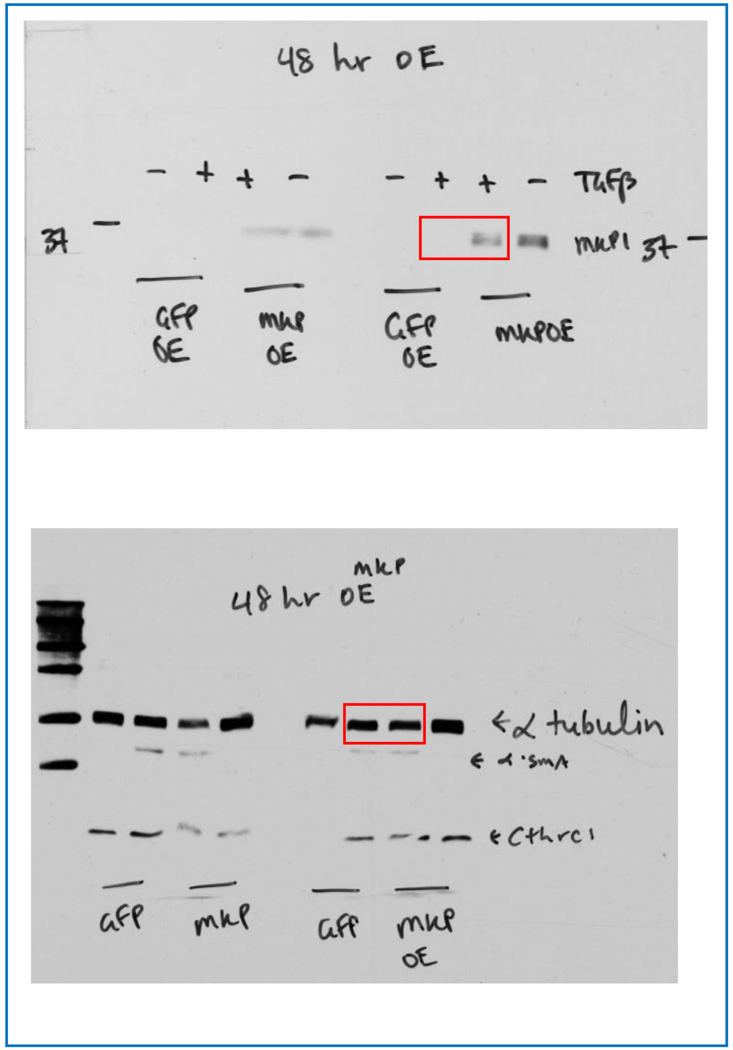

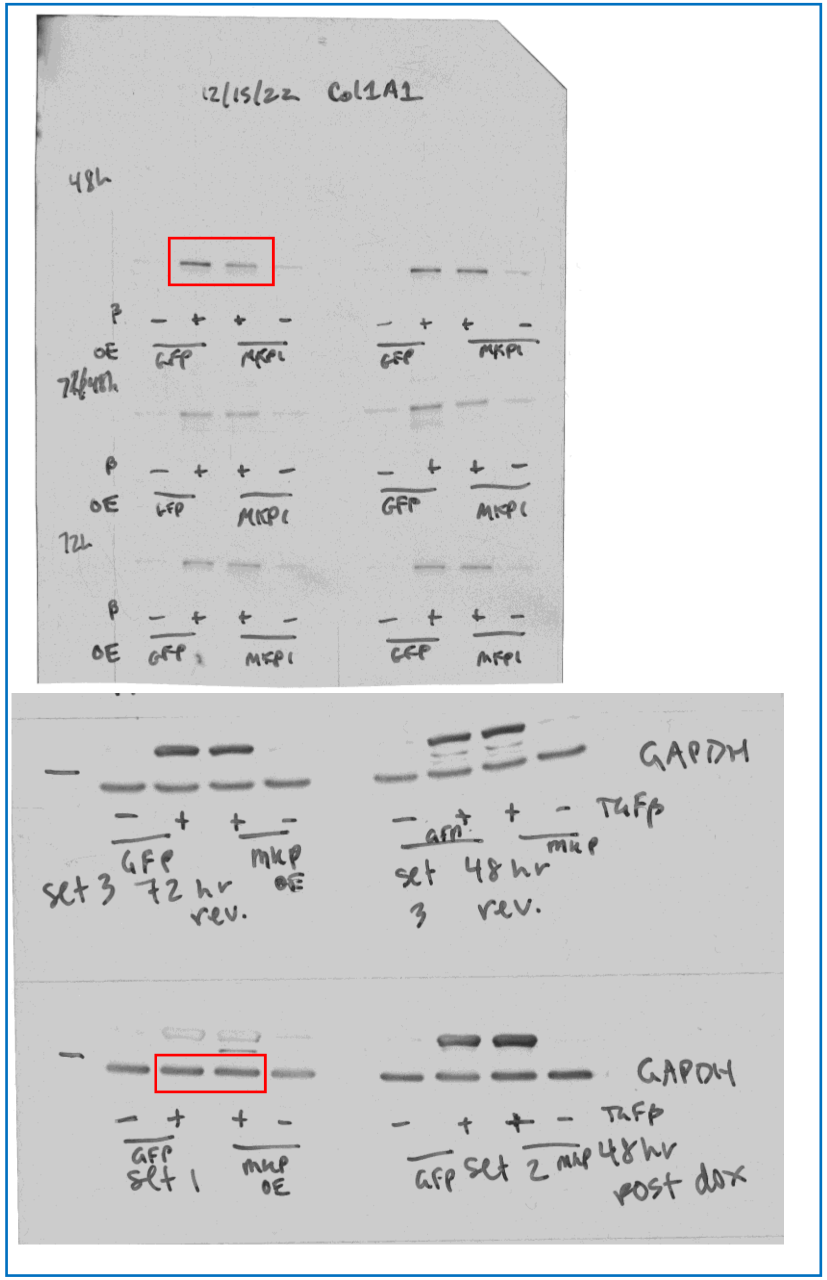

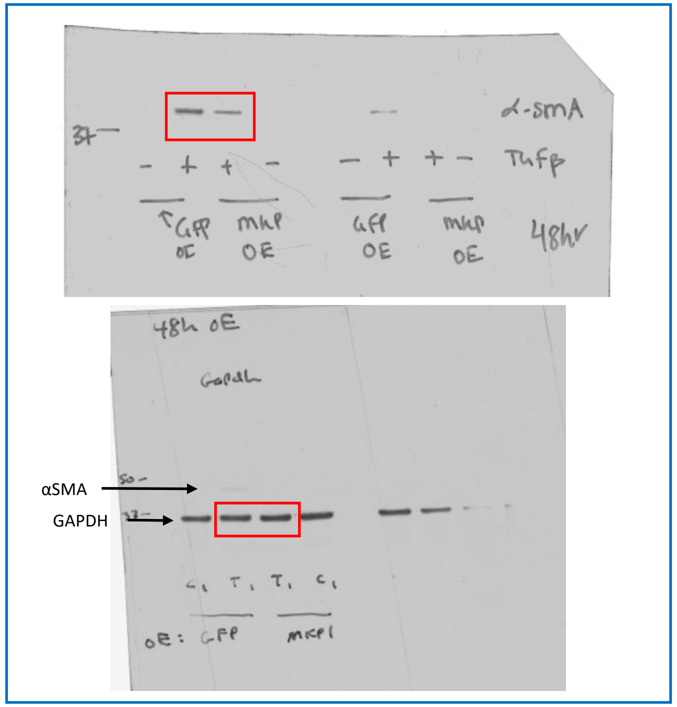

MIDDLE: GAPDH (BOTTOM LEFT) was run in the same lanes on the same gel as the Col1a1 bands (TOP LEFT). The membrane was cut and developed separately.

Full unedited blots for Figure 2B: FN1 and CTHRC1

LEFT: GAPDH (BOTTOM) was run in the same lanes on the same gel as the fibronectin bands (TOP). The membrane was cut and developed separately.

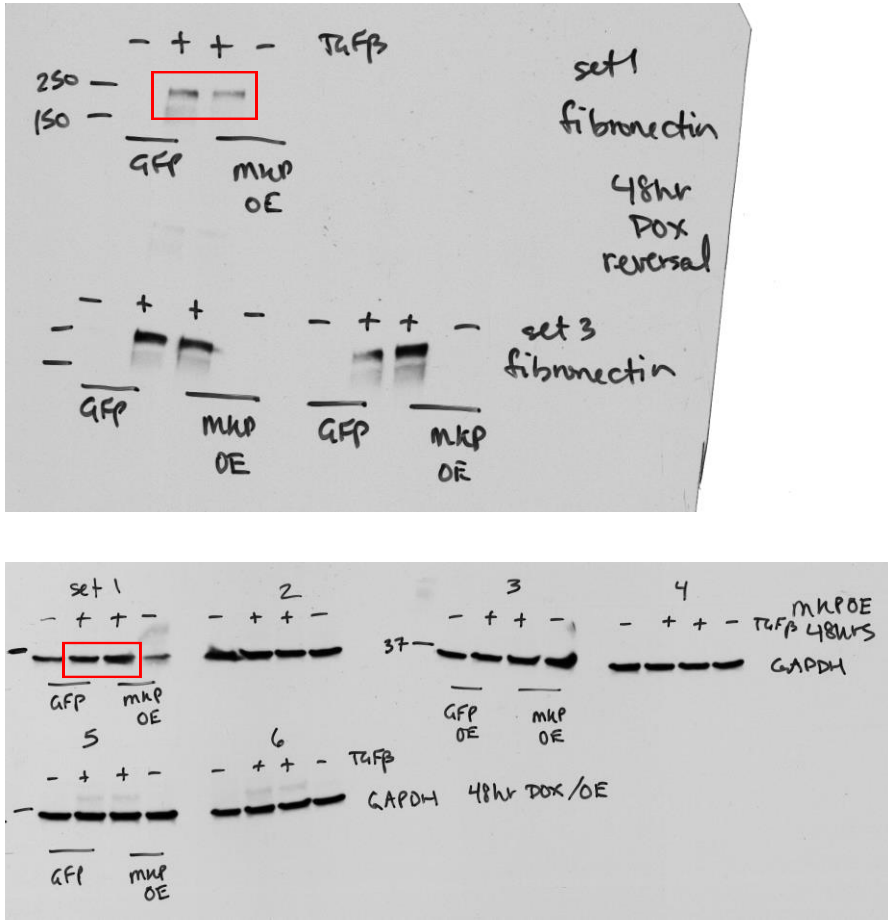

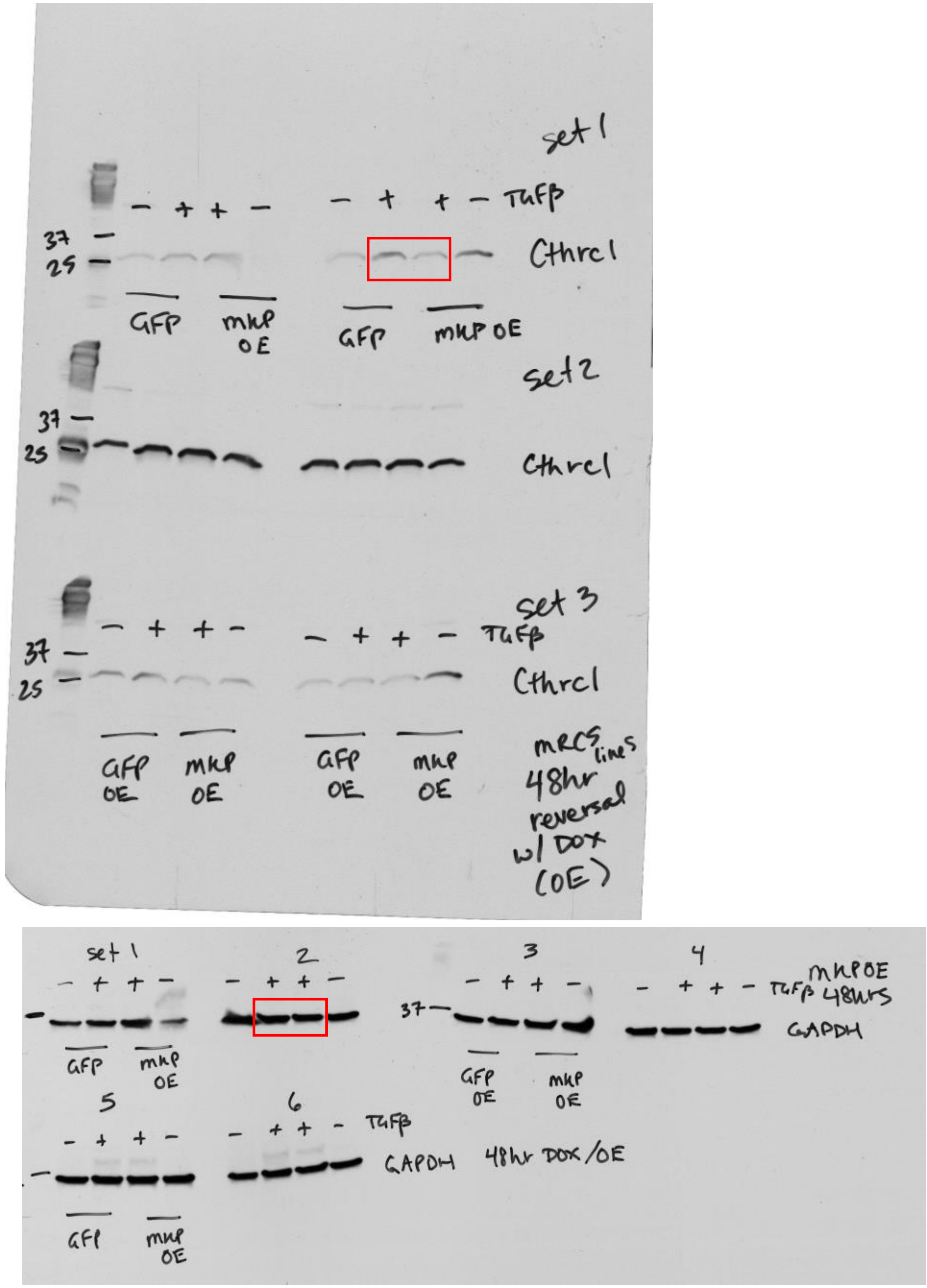

Full unedited blots for Figure 2D:

Collagen was run on the same gel as SMA and the corresponding GAPDH. Likewise, FN1 was run on the same gel as CTHRC1 and its corresponding GAPDH loading control. For collagen and FN1, the membrane was cut and developed separately.

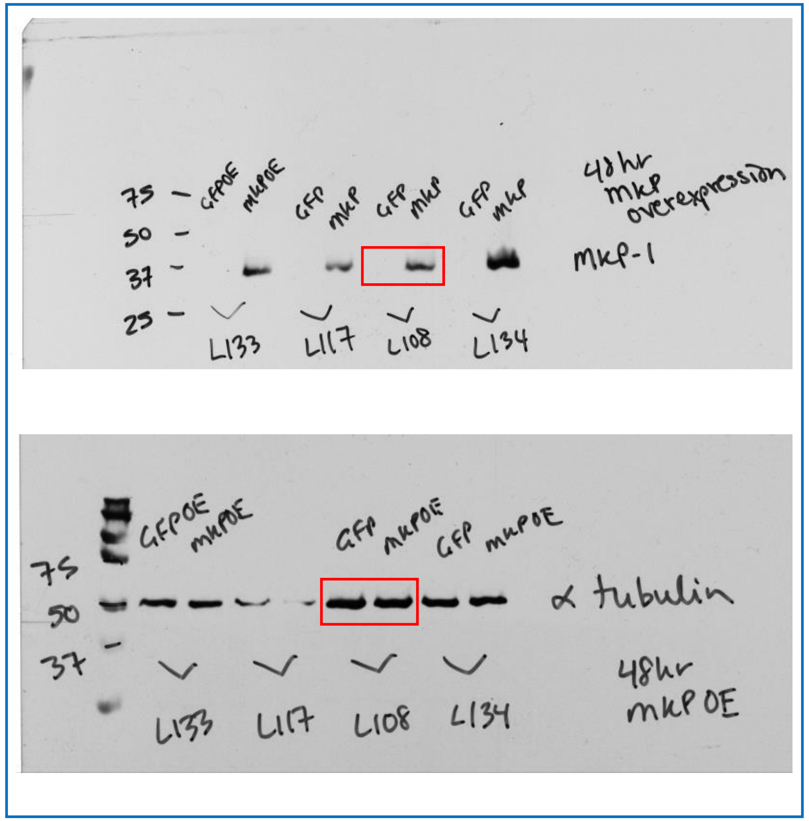

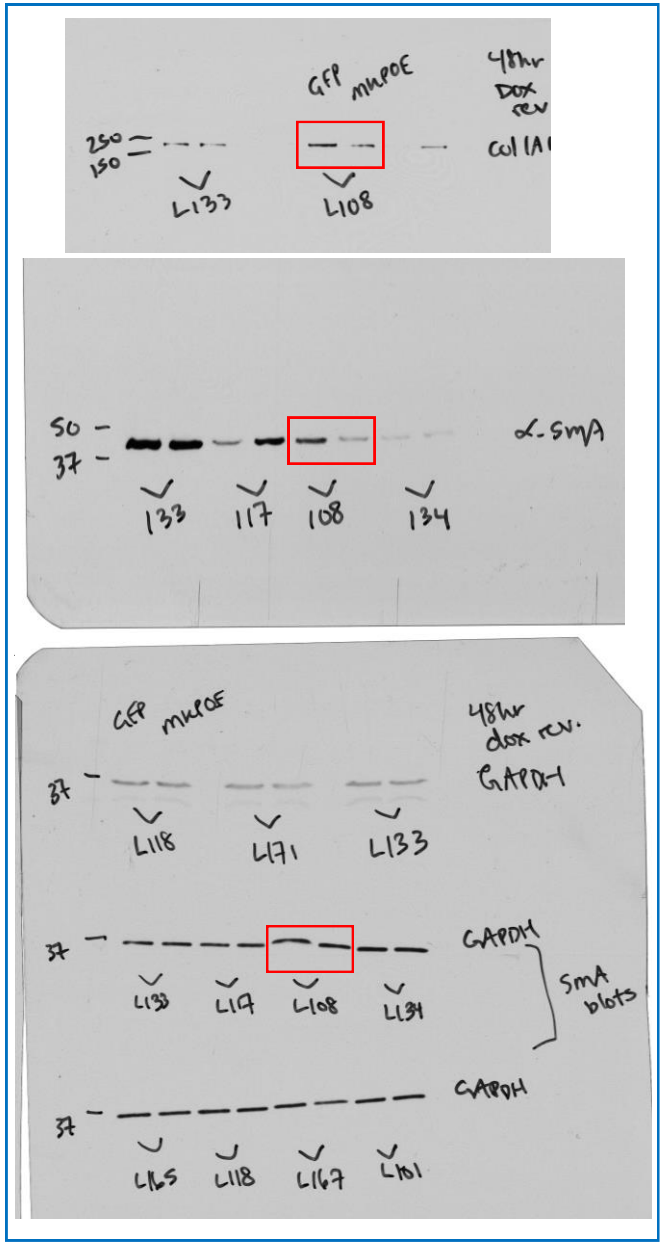

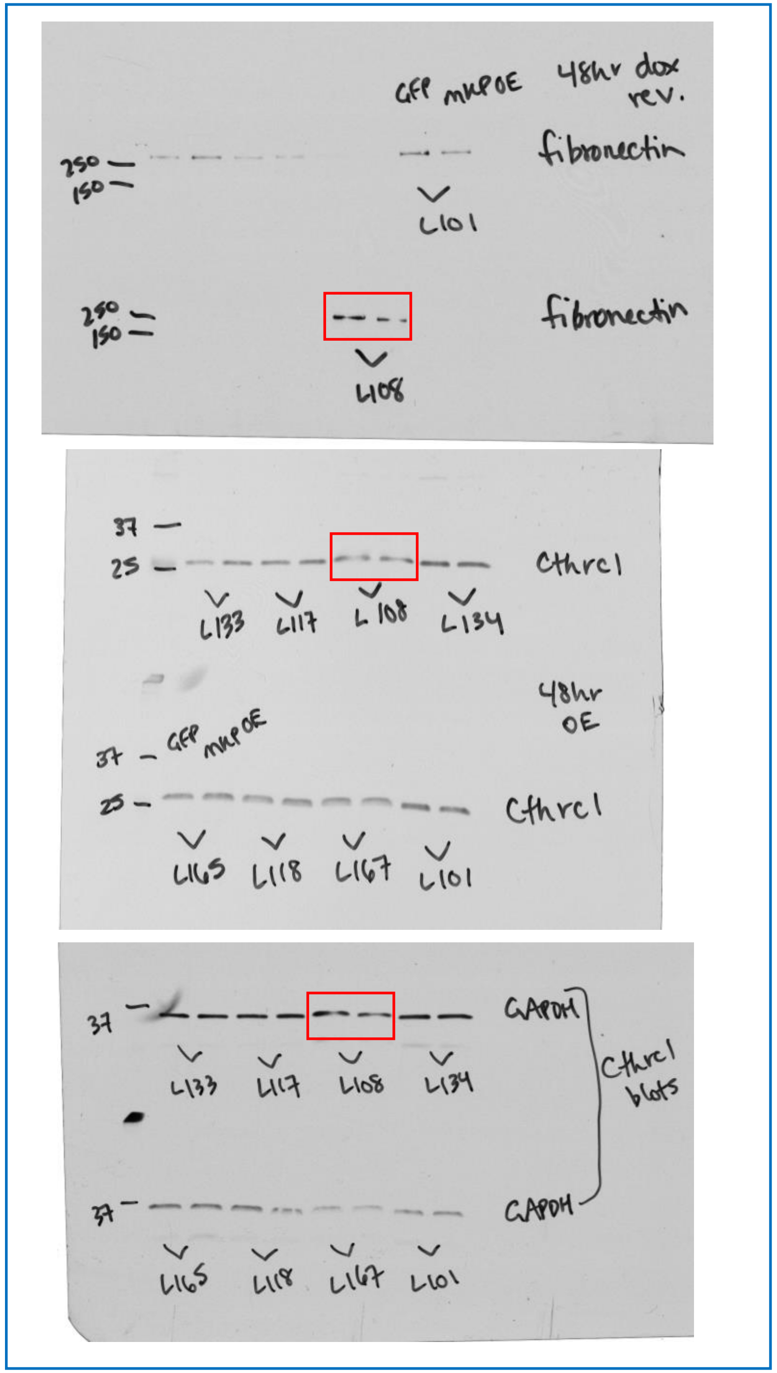

Full unedited blots for Figure 3B:

Left

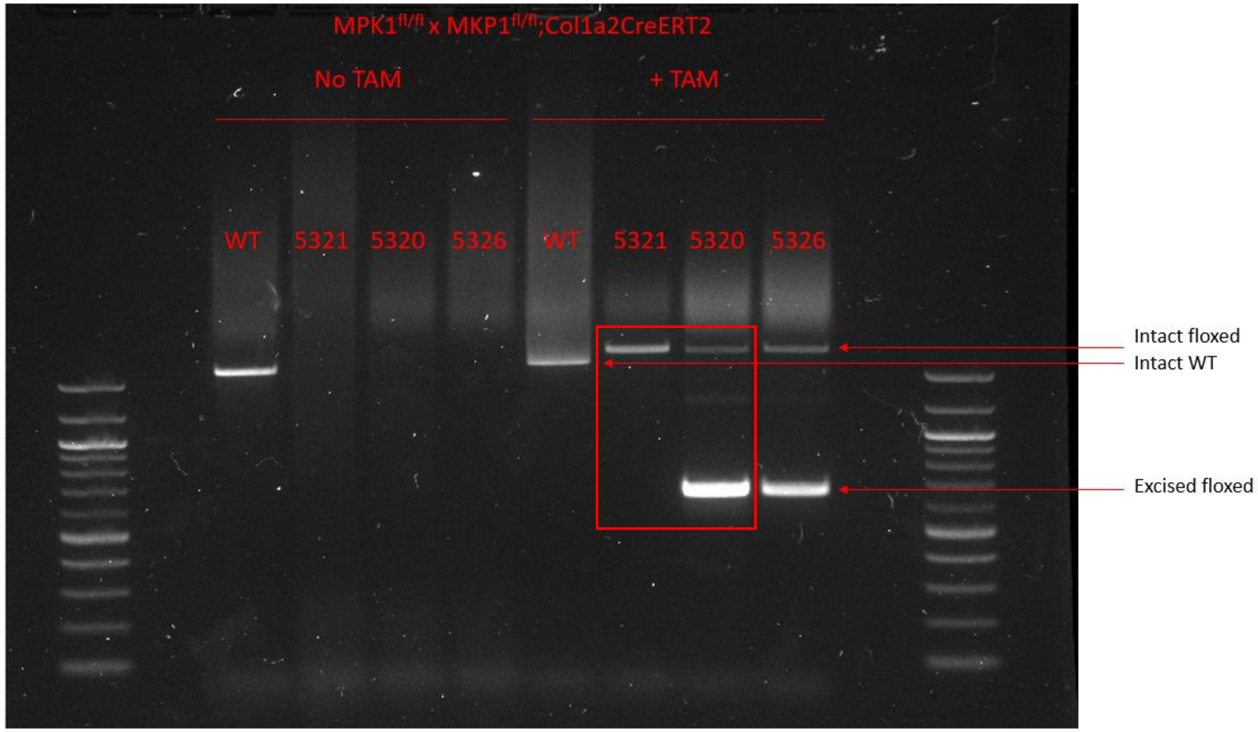

Right

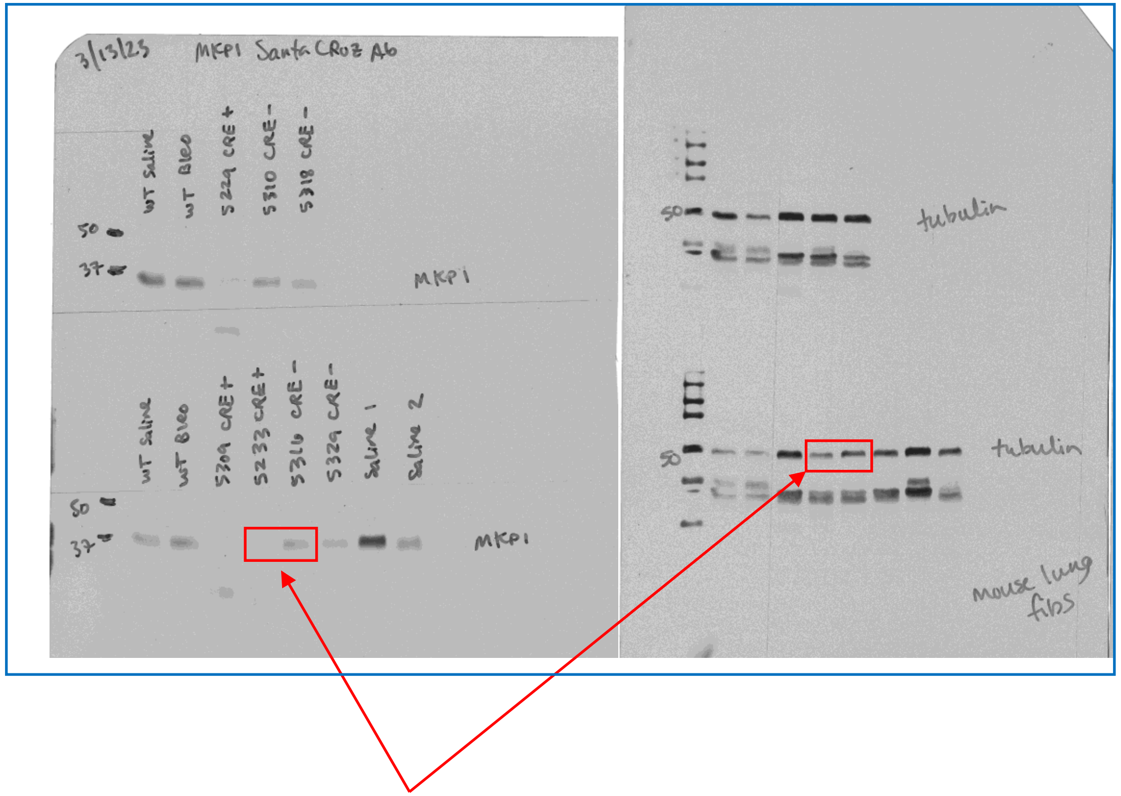

Each pair of bands was horizontally flipped for the final figure so that “Cre –” displayed to the left of “Cre+”.

Full unedited blots for Figure 4A:

For each MAPK blot below (p38, ERK, JNK), the membrane was probed with the p-MAPK primary antibody followed by anti-rabbit HRP secondary antibody and developed. The membrane was then stripped and probed with total MAPK primary followed by anti-rabbit HRP secondary and developed.

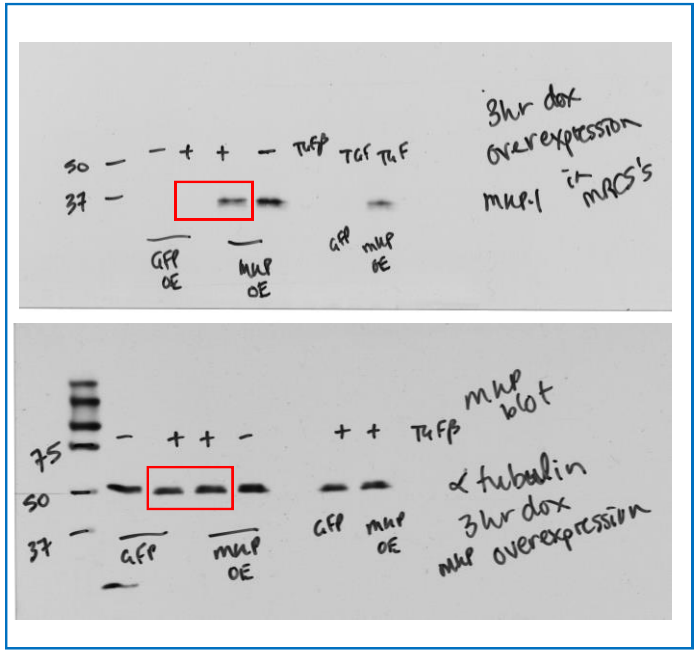

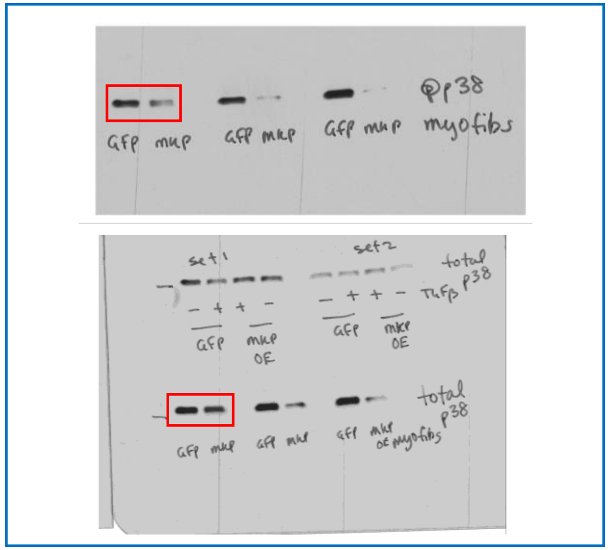

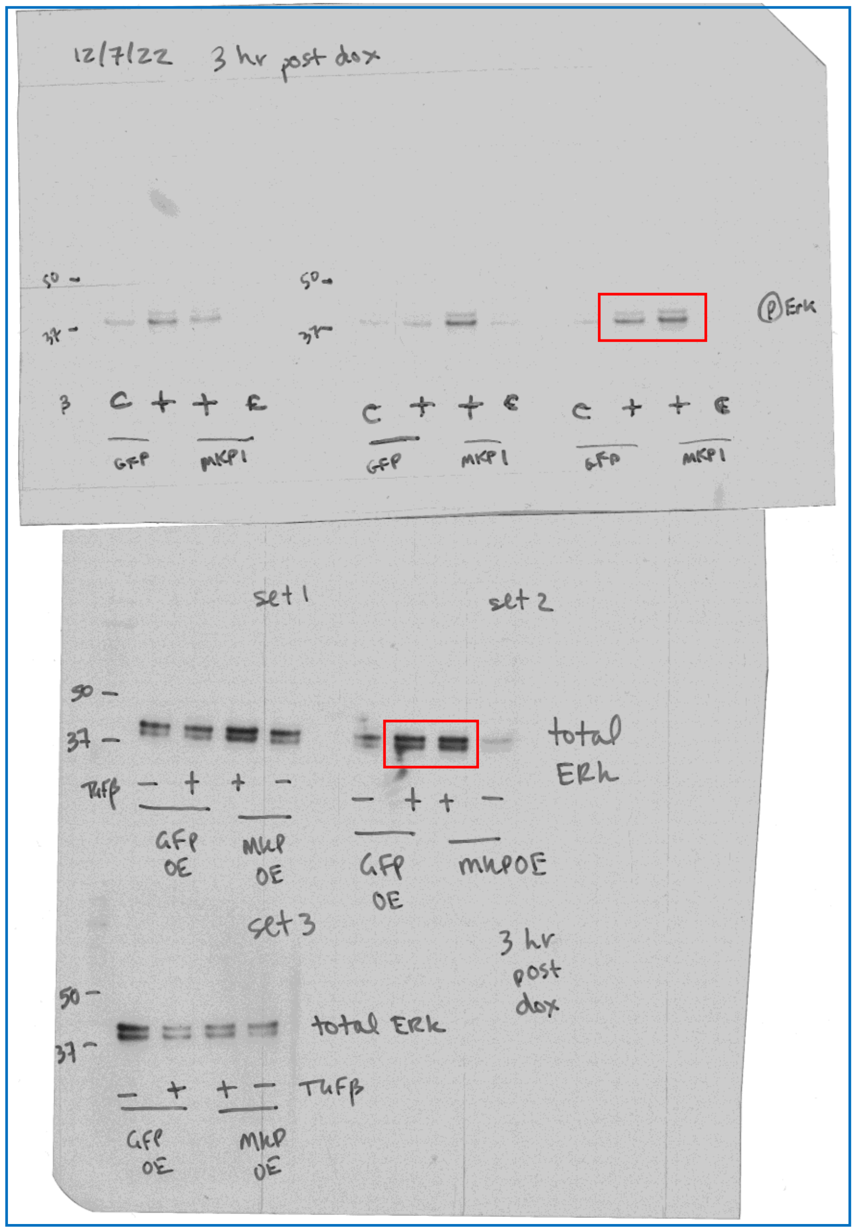

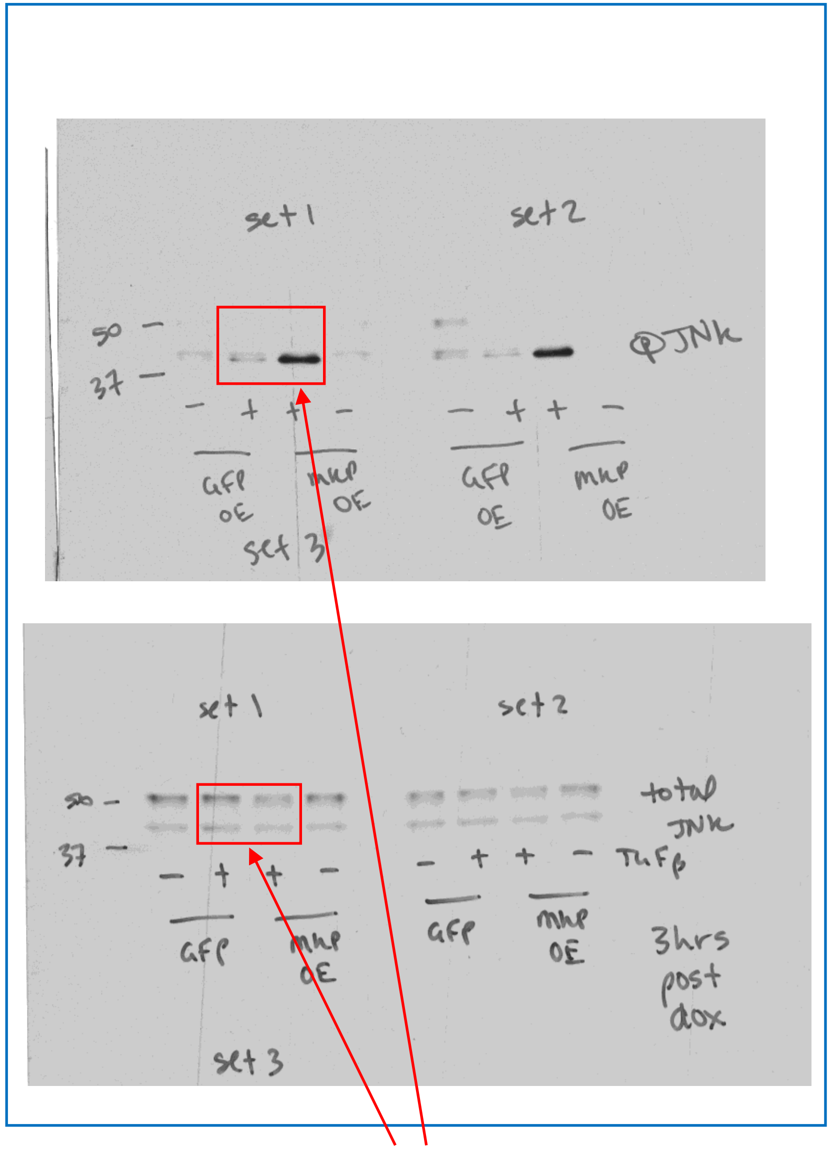

Compressed vertically in final Figure to fit into cropped image

Full unedited blots for Figure 4B: MKP1

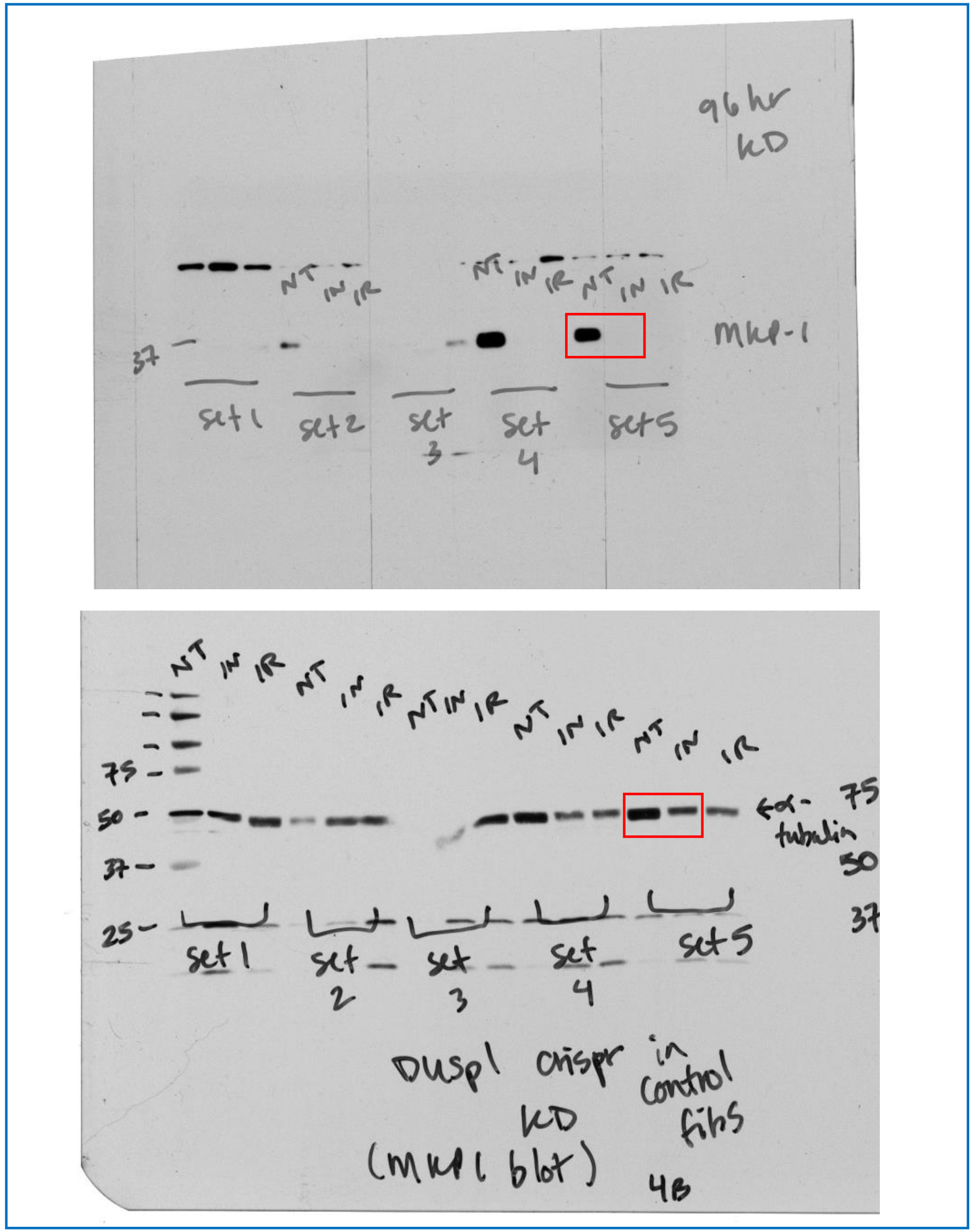

Full unedited blots for Figure 4B: p38, ERK, JNK

For each MAPK blot below (ERK and JNK), the membrane was probed with the p-MAPK primary antibody followed by anti-rabbit HRP secondary antibody and developed. The membrane was then stripped and probed with total MAPK primary followed by anti-rabbit HRP secondary and developed.

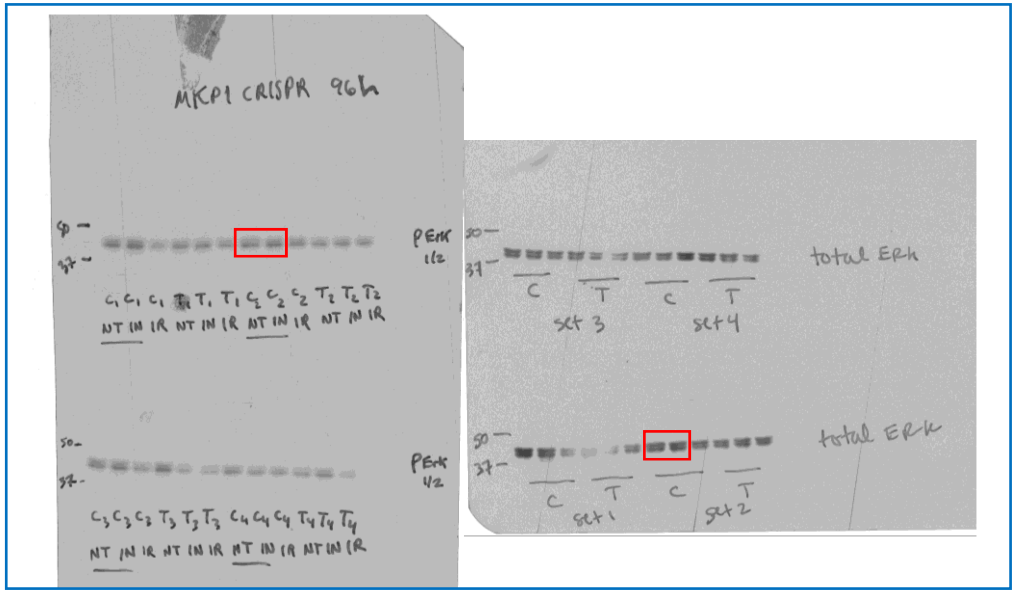

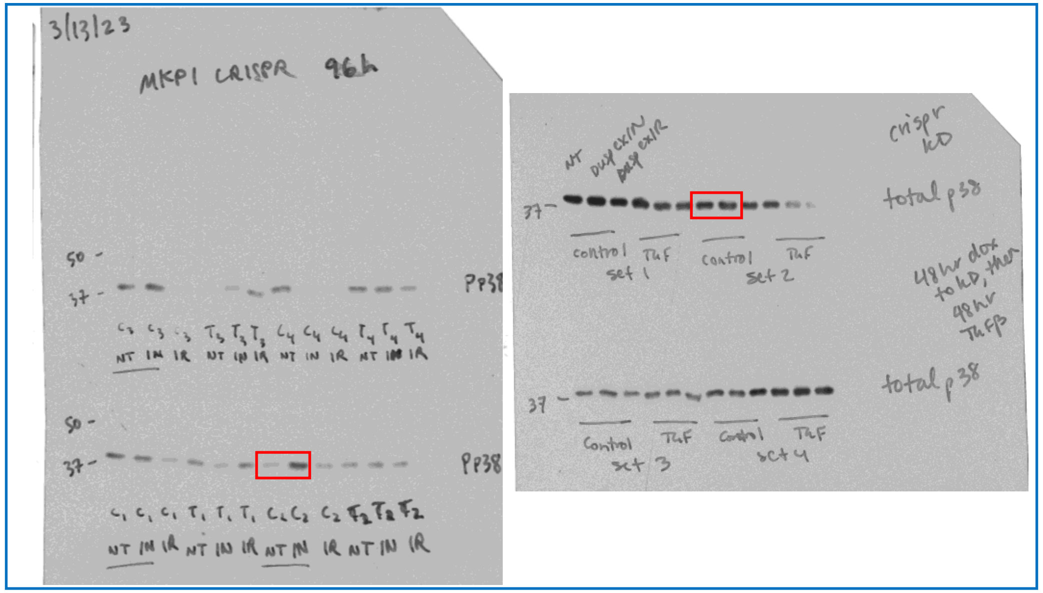

Compressed vertically in final Figure to fit into cropped image

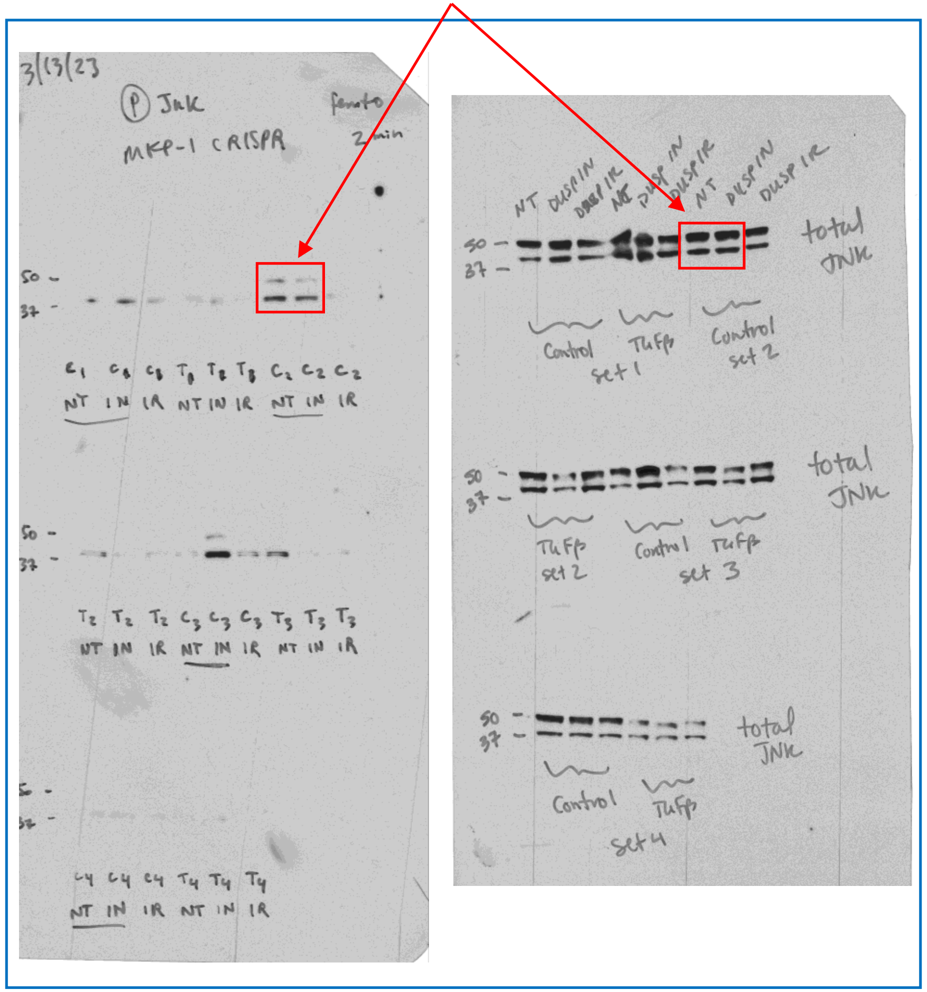

Full unedited blots for Figure 4C:

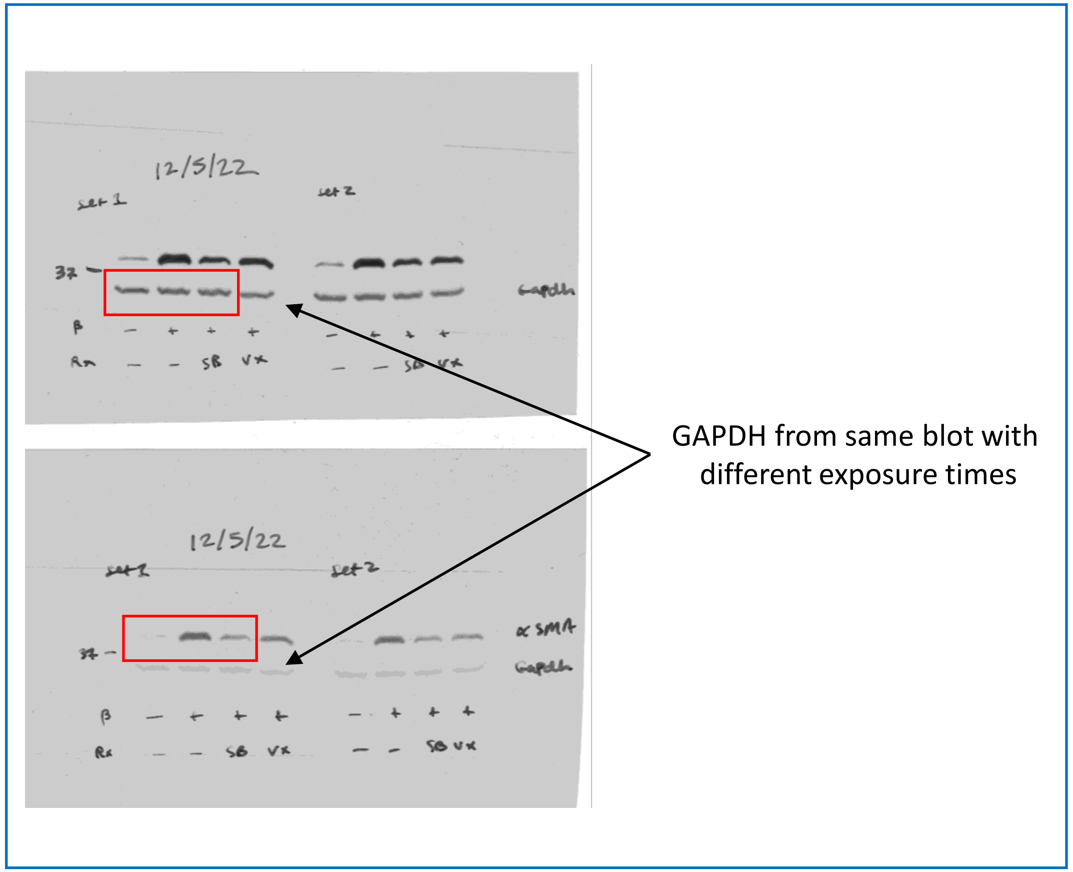

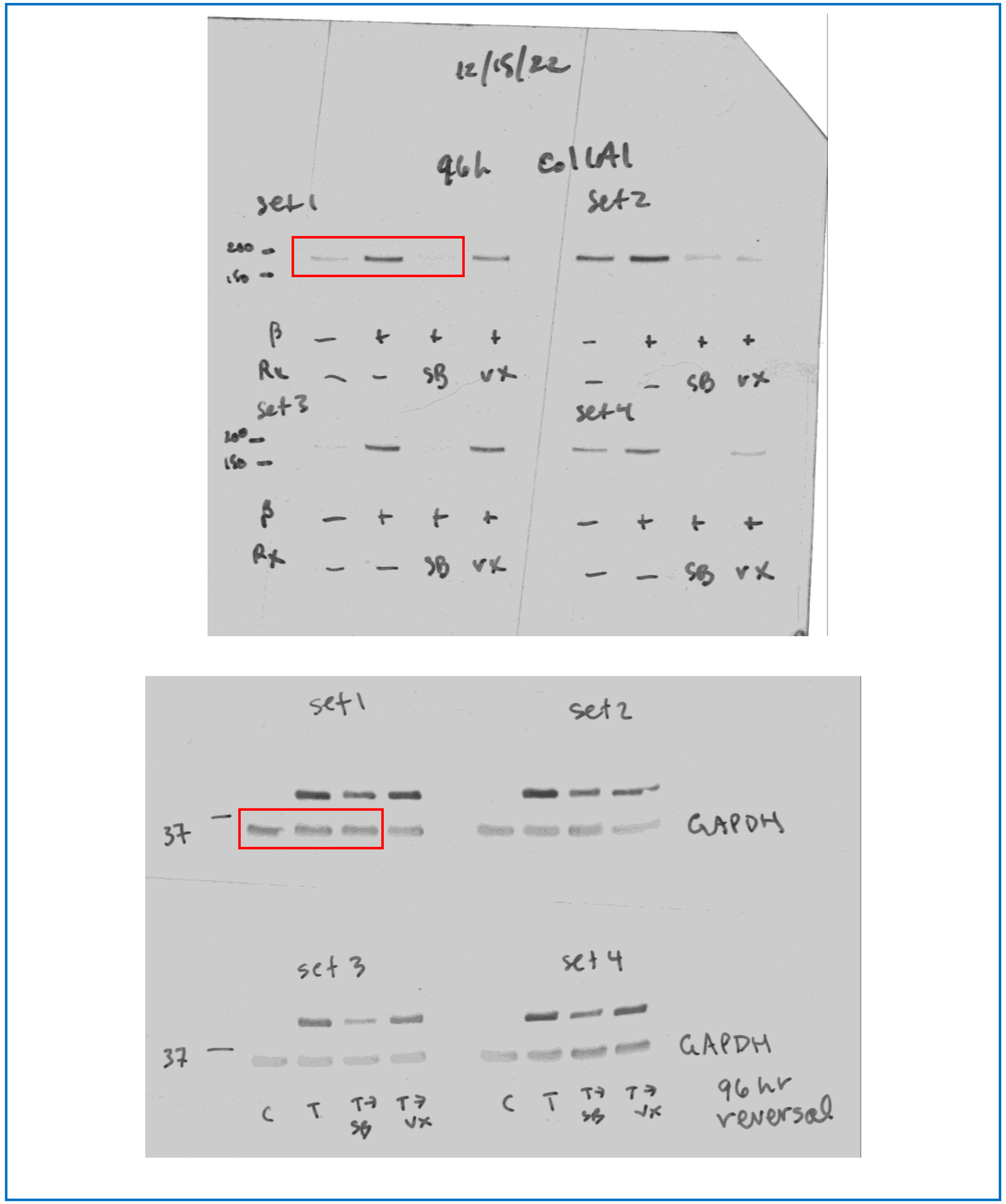

Full unedited blots for Figure 5B:

LEFT: The membrane was probed with total p38 primary antibody followed by anti-rabbit HRP secondary antibody and developed. The membrane was then probed for alpha-tubulin followed by anti-mouse secondary antibody HRP and developed.

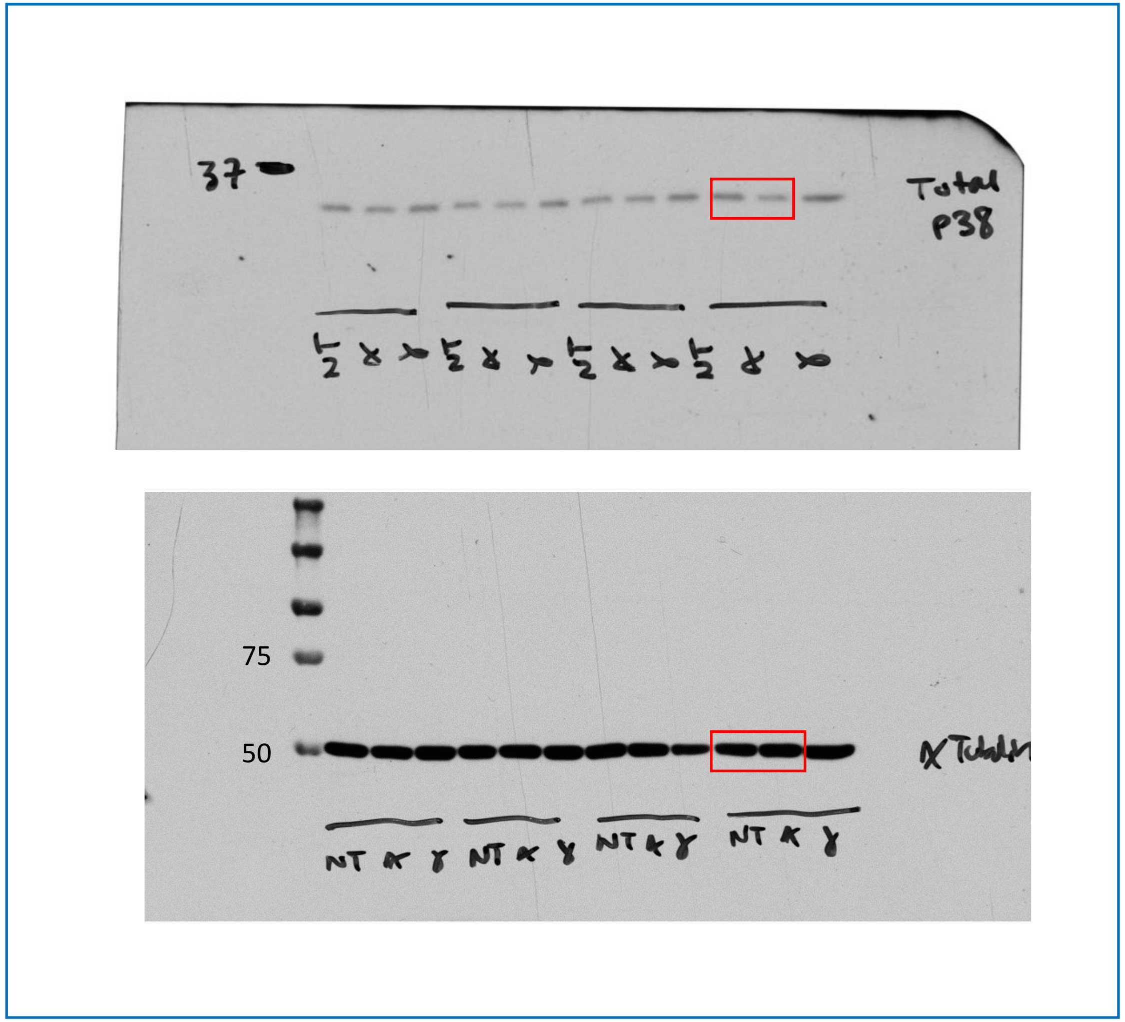

RIGHT: The membrane was probed for p38-alpha primary antibody, then anti-rabbit secondary HRP antibody and developed. The membrane was then stripped and re-probed with GAPDH primary HRP antibody and developed.

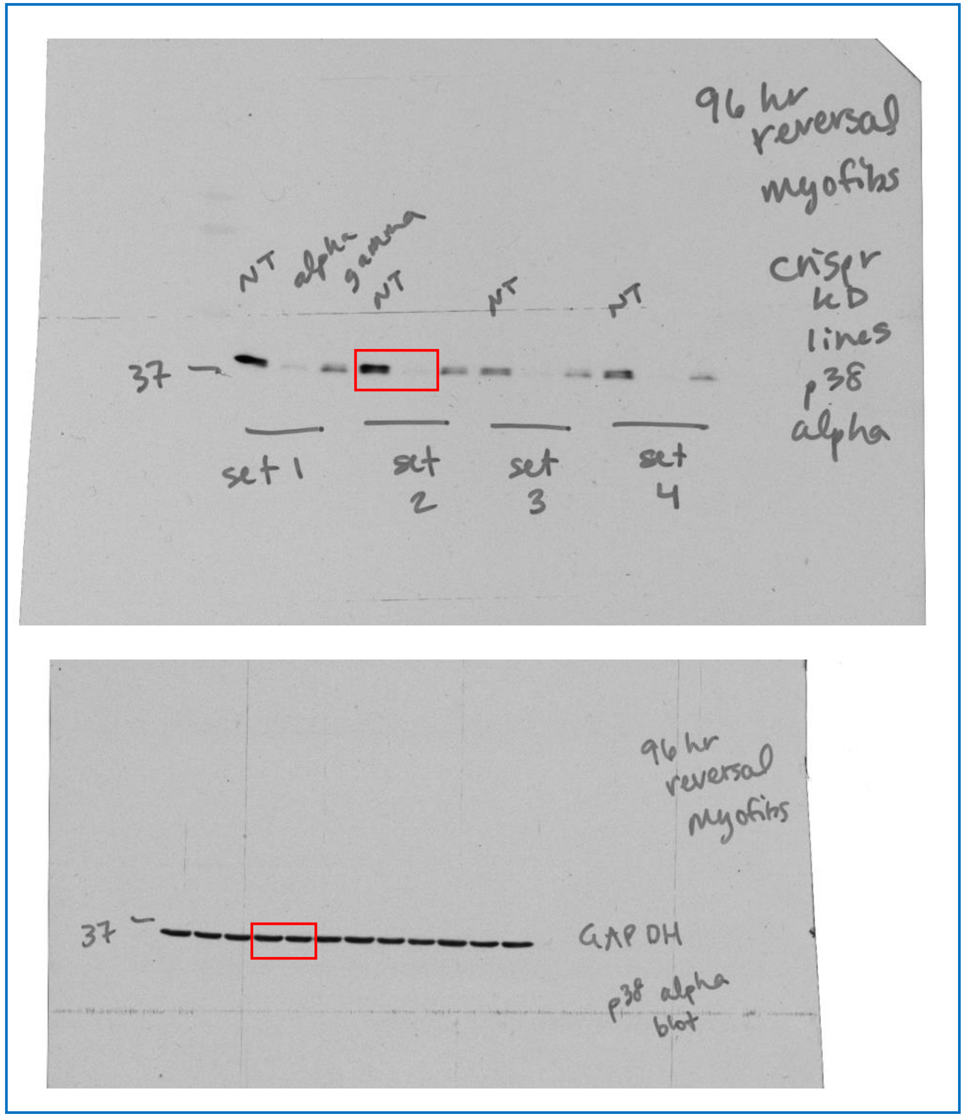

Full unedited blots for Figure 5C:

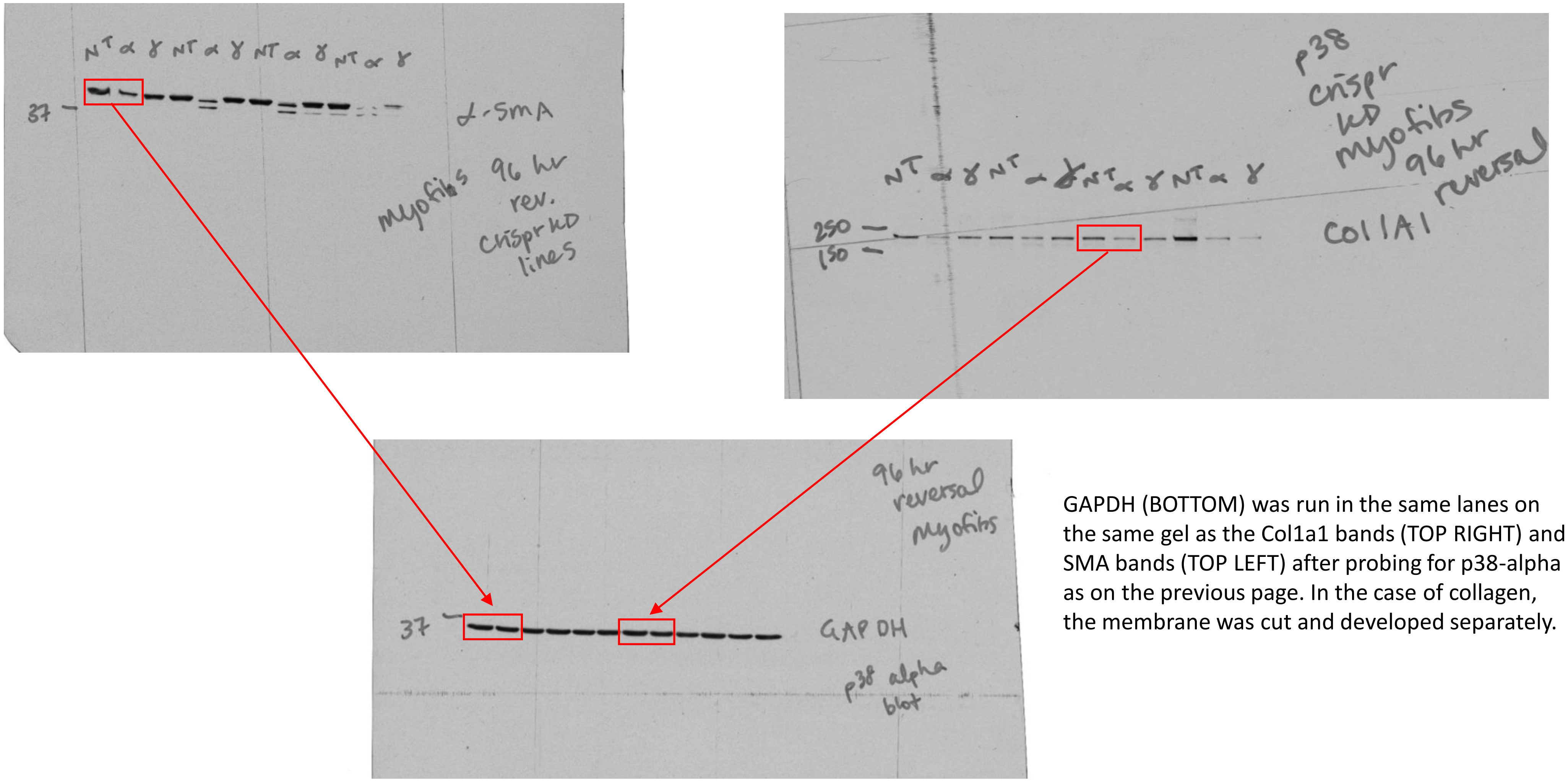

GAPDH (BOTTOM) was run in the same lanes on the same gel as the Col1a1 bands (TOP RIGHT) and SMA bands (TOP LEFT) after probing for p38-alpha as on the previous page. In the case of collagen, the membrane was cut and developed separately.

Full unedited blots for Figure 5D:

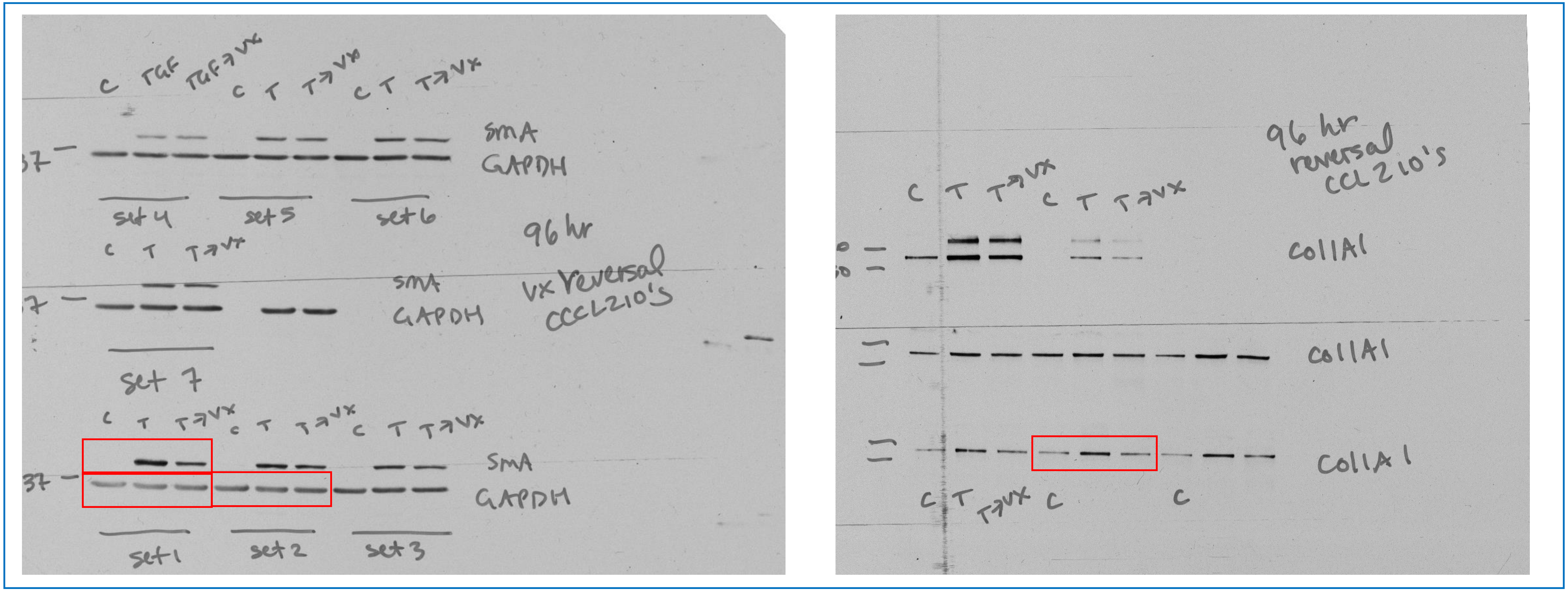

GAPDH from set 2 (LEFT) was run in the same lanes on the same gel as the Col1a1 bands (RIGHT). The membrane was cut and developed separately.

Full unedited blots for Figure 7A:

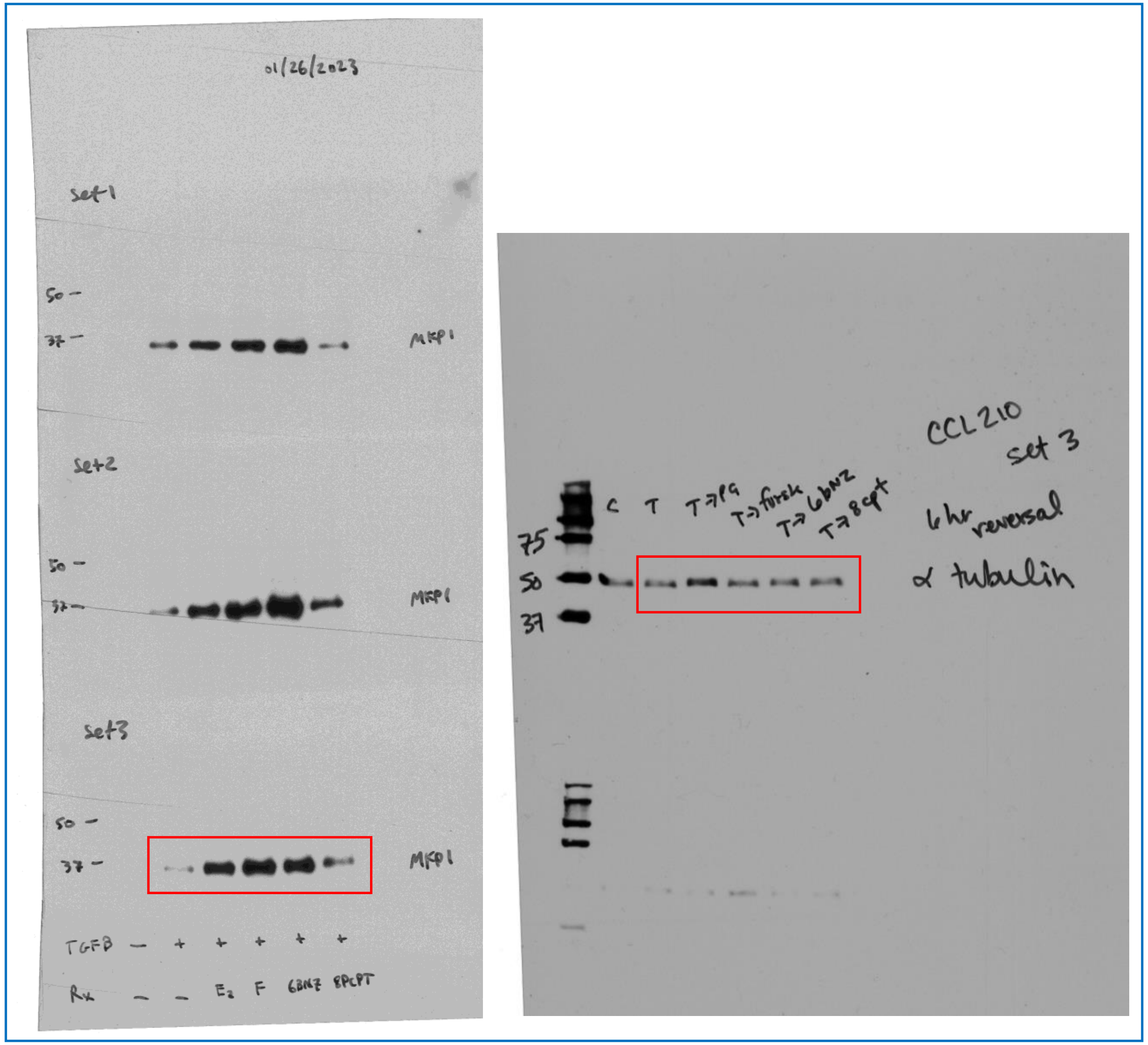

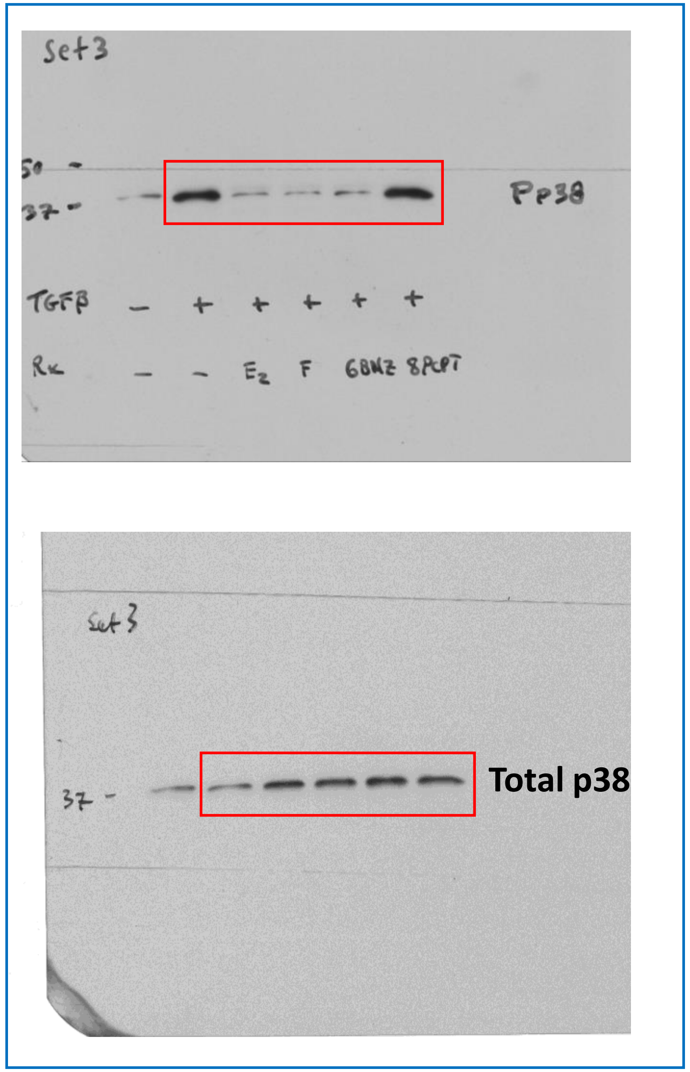

The membrane was probed with p-p38 primary antibody followed by anti-rabbit HRP secondary antibody and developed. The membrane was then stripped and probed with total p38 primary followed by anti-rabbit HRP secondary and developed.

Full unedited blots for Figure 7B: Human fibroblasts – Top

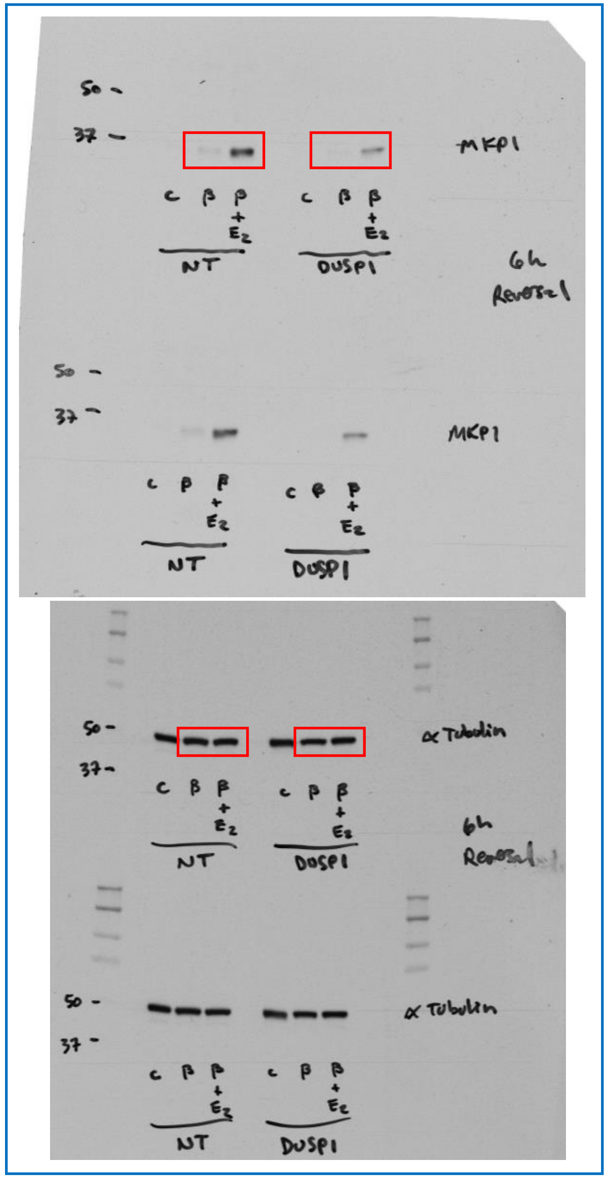

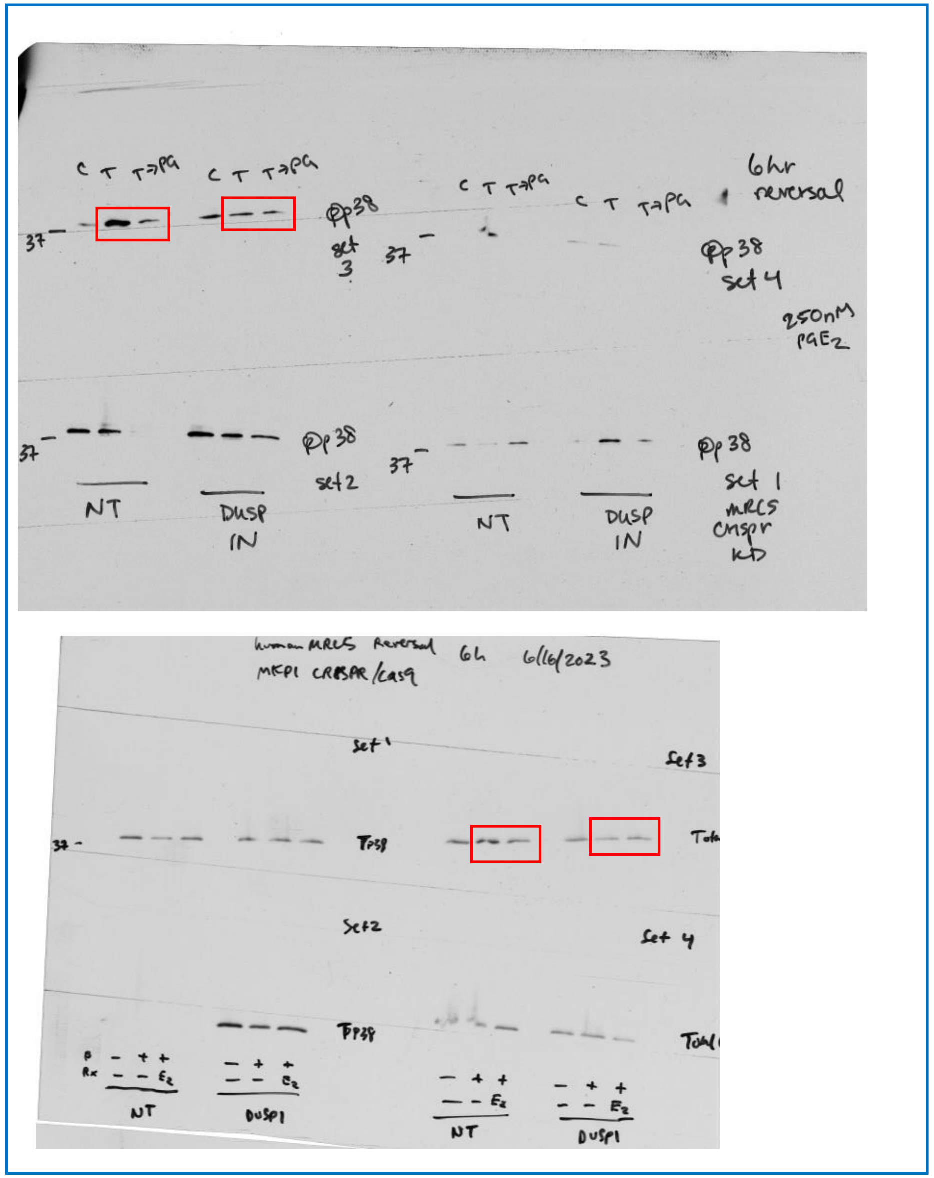

RIGHT: The membrane was probed with p-p38 primary antibody followed by anti-rabbit HRP secondary antibody and developed. The membrane was then stripped and probed with total p38 primary followed by anti-rabbit HRP secondary and developed.

Full unedited blots for Figure 7B: Mouse fibroblasts – Bottom

Each membrane was probed with p-p38 primary antibody followed by anti-rabbit HRP secondary antibody and developed. Each membrane was then stripped and probed with total p38 primary followed by anti-rabbit HRP secondary and developed.

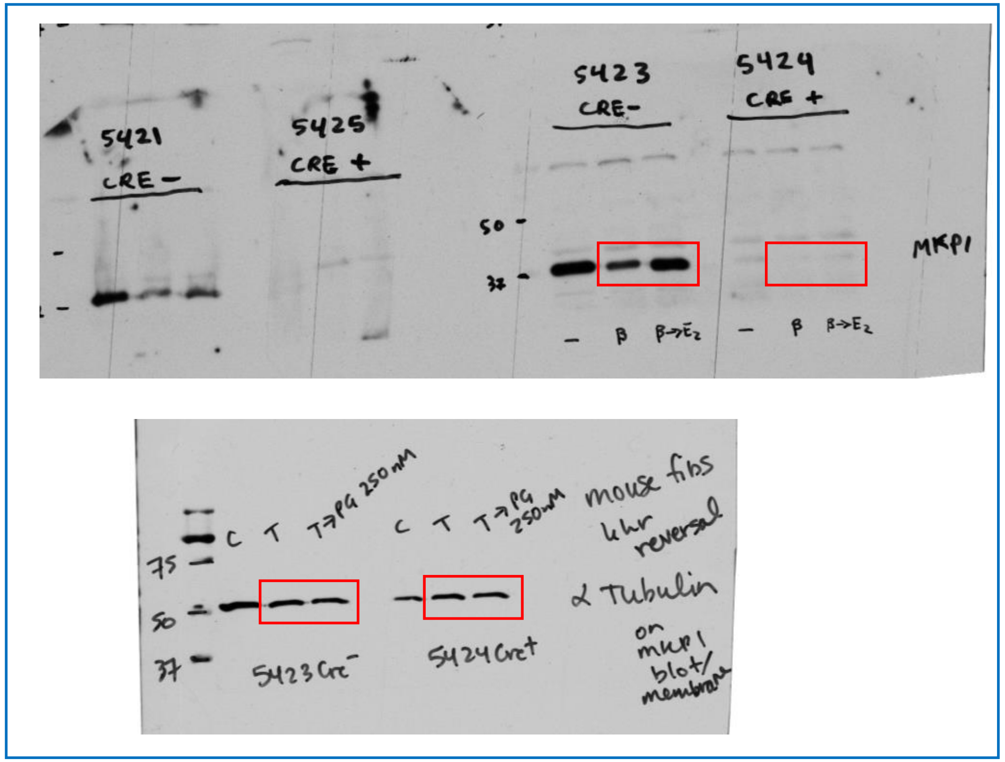

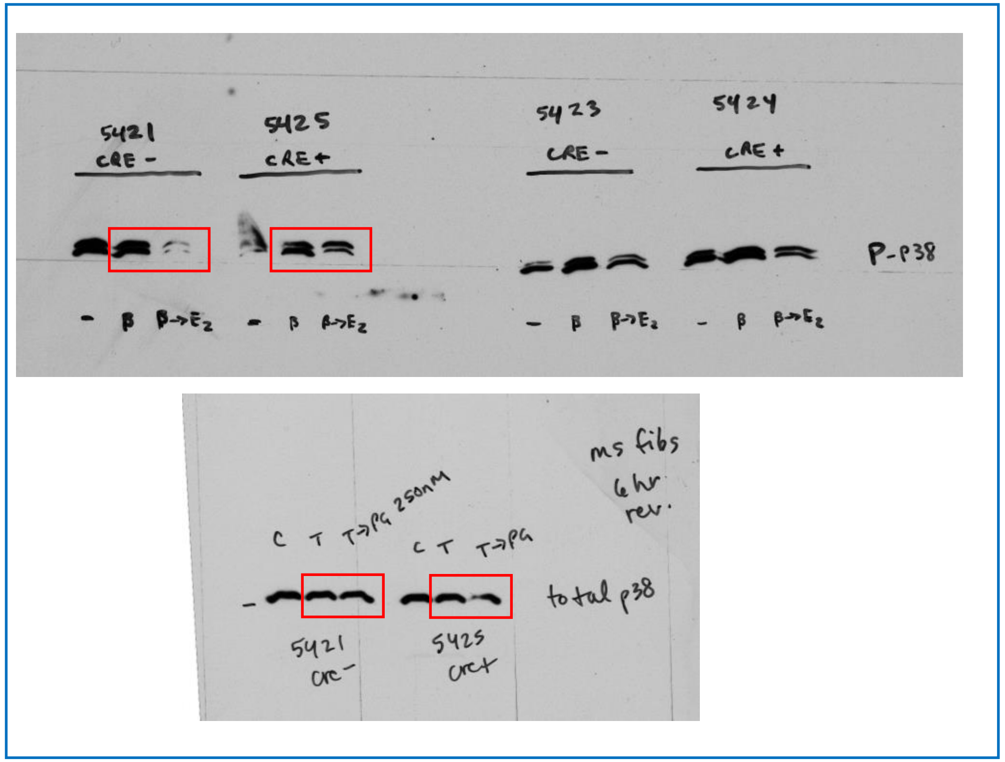

Full unedited blots for Figure 7C:

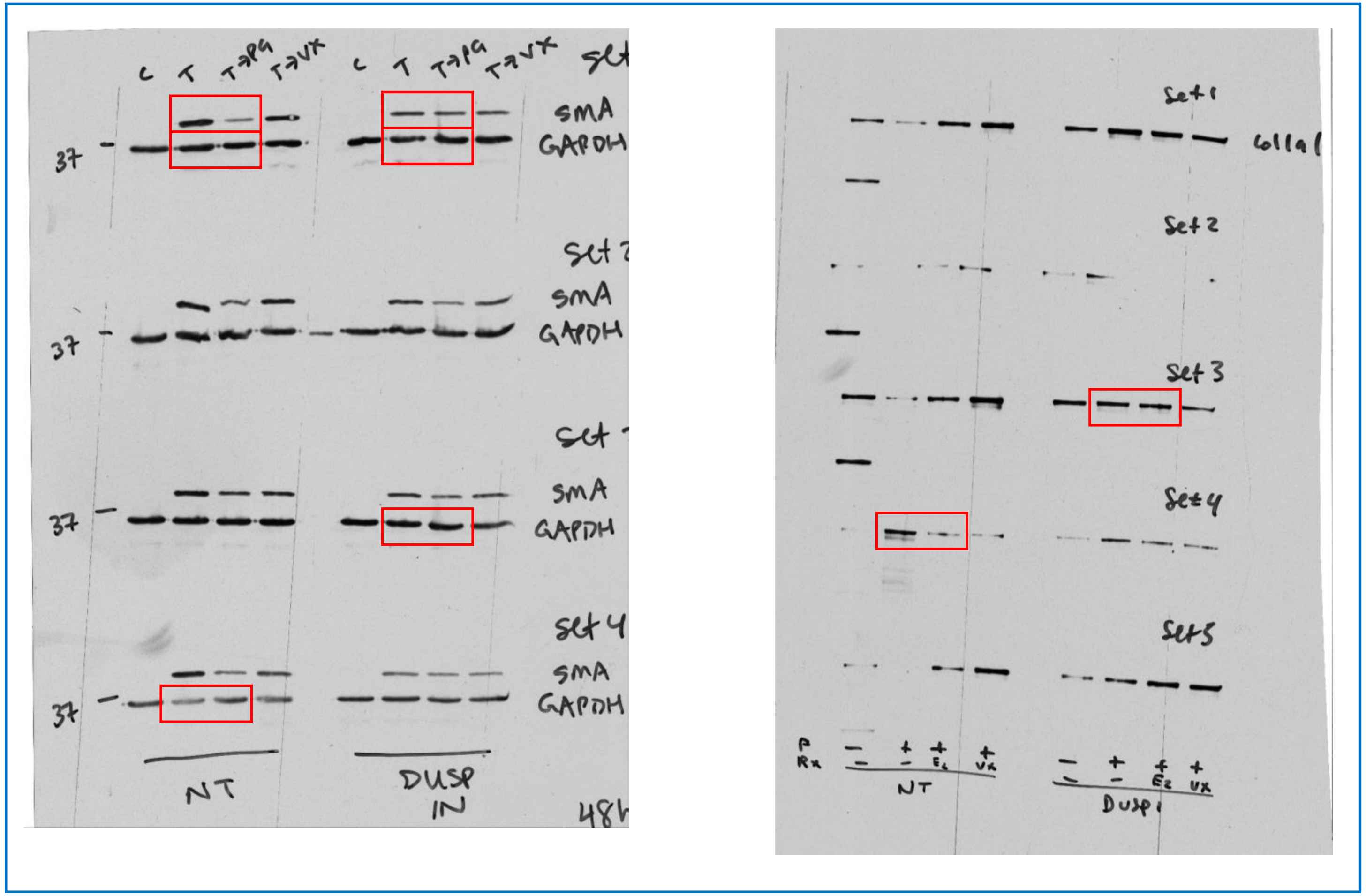

The membranes probed for Col1a1 (RIGHT) were cut from the membranes probed for SMA and GAPDH (LEFT) and developed separately.

Full unedited blots for Figure 8B: Prevention – Top

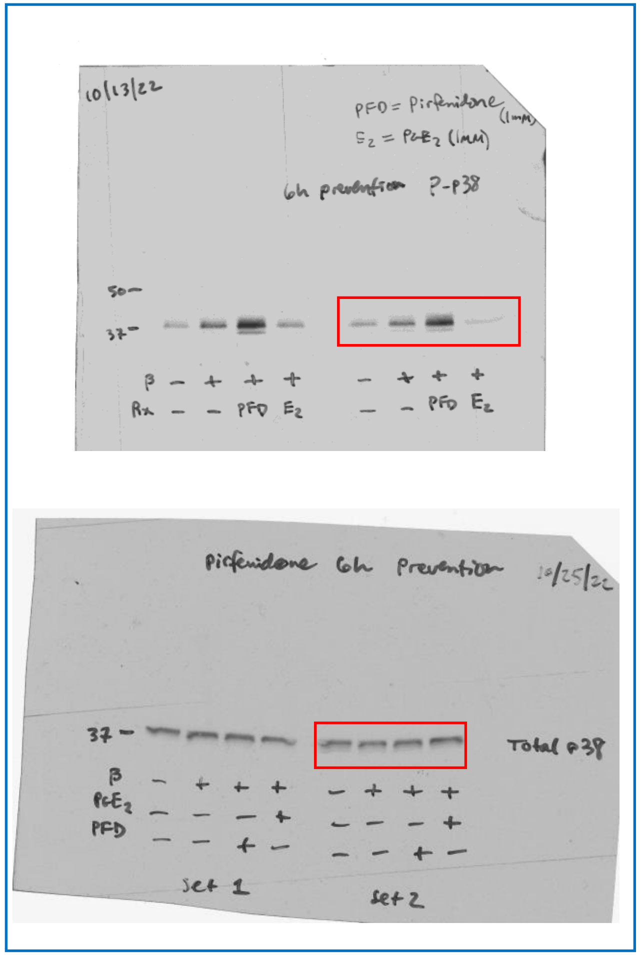

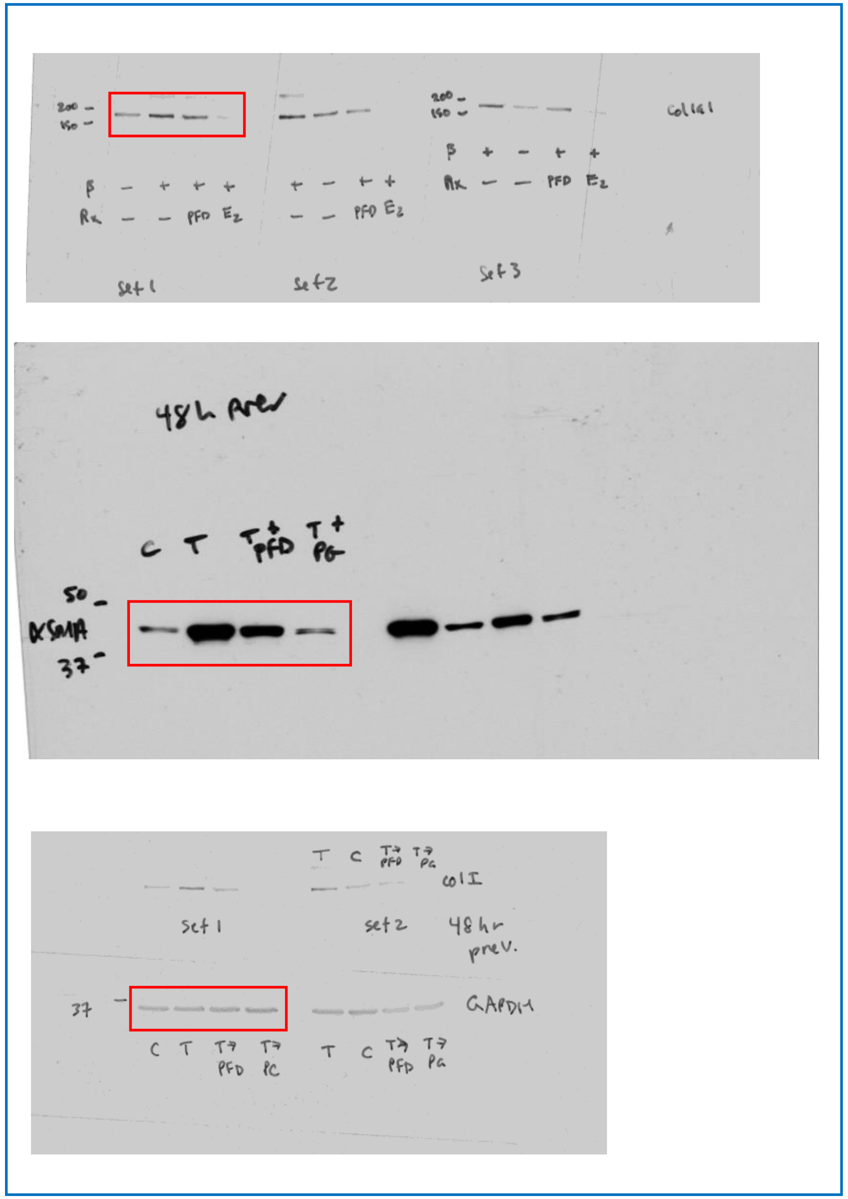

LEFT: The membrane was probed with p-p38 primary antibody followed by anti-rabbit HRP secondary antibody and developed. The membrane was then stripped and probed with total p38 primary followed by anti-rabbit HRP secondary and developed.

Collagen was probed first followed by GAPDH on the same membrane. Finally, SMA was probed last on the same membrane.

Full unedited blots for Figure 8B: Reversal – Bottom

Compressed vertically in final Figure to fit into cropped image

The bands were horizontally flipped in the final figure to maintain the following order: control, TGFb, TGFb+PFD, TGFb+PGE$_2$

Reversed order (right to left on gel)

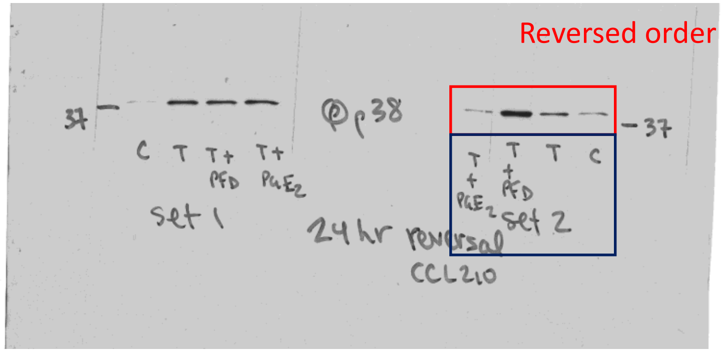

Reversed order (right to left on gel)

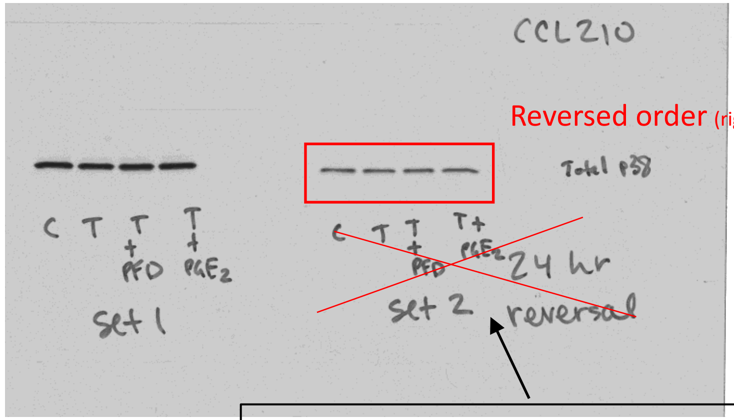

Mislabeled. The correct order (left to right) is:
TGFb+PGE2, TGFb+PFD, TGFb, Control

LEFT: The membrane was probed with p-p38 primary antibody followed by anti-rabbit HRP secondary antibody and developed. The membrane was then stripped and probed with total p38 primary followed by anti-rabbit HRP secondary and developed.

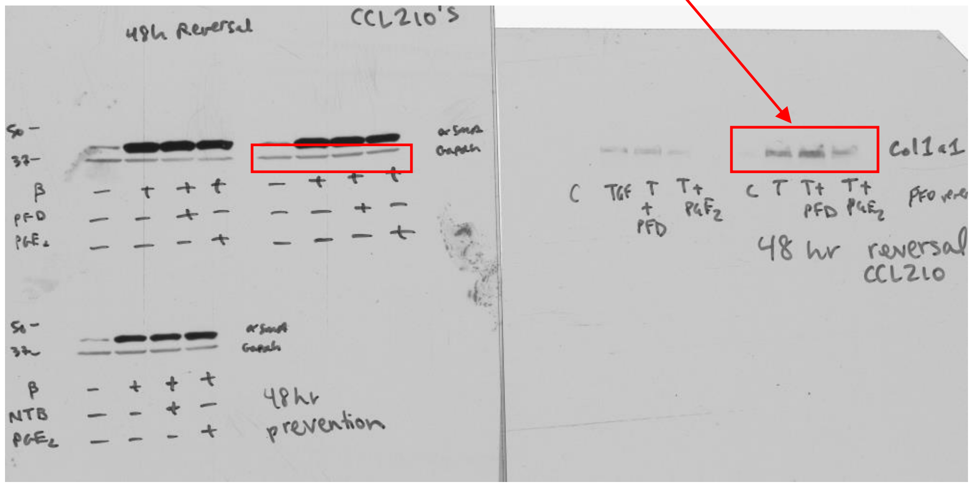

GAPDH (TOP) was run in the same lanes on the same gel as the Col1a1 bands (TOP RIGHT). The membrane was cut and developed separately.

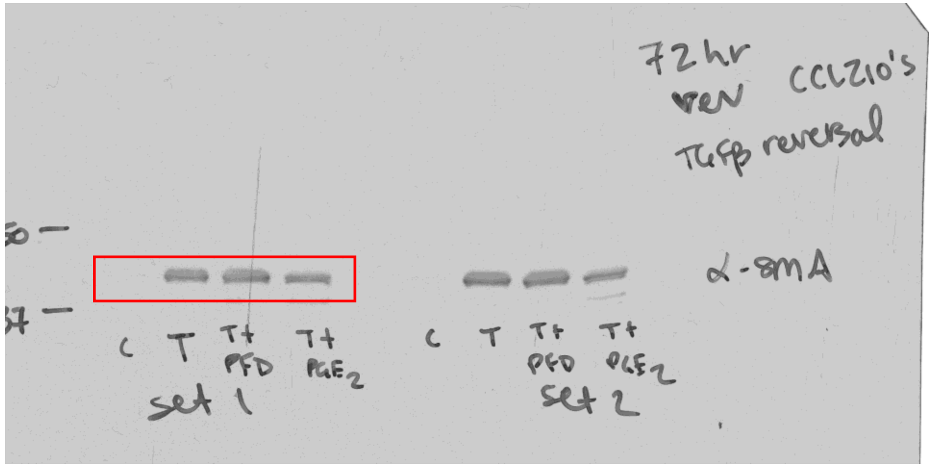

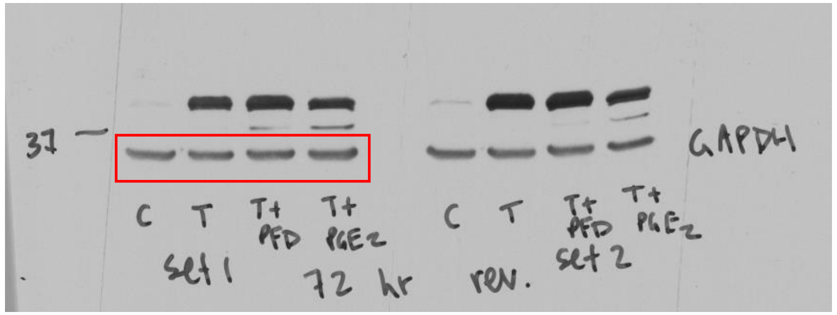

Full unedited blots for Figure 8C: Prevention – Top

LEFT**:** The membrane was probed with p-p38 primary antibody followed by anti-rabbit HRP secondary antibody and developed. The membrane was then stripped and probed with total p38 primary followed by anti-rabbit HRP secondary and developed.

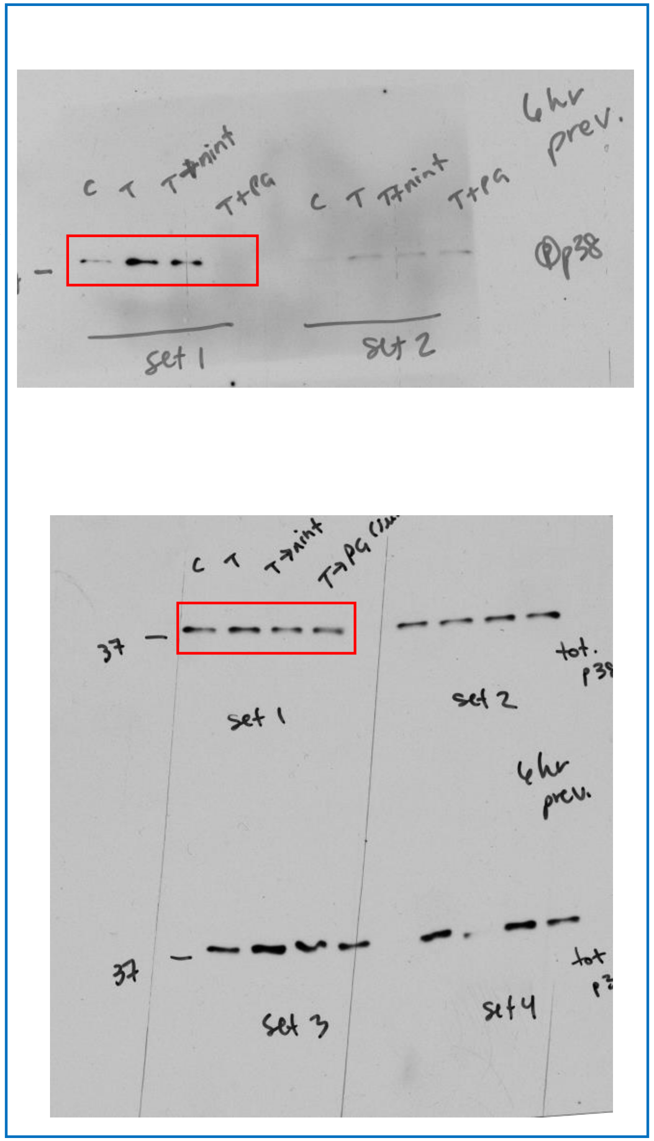

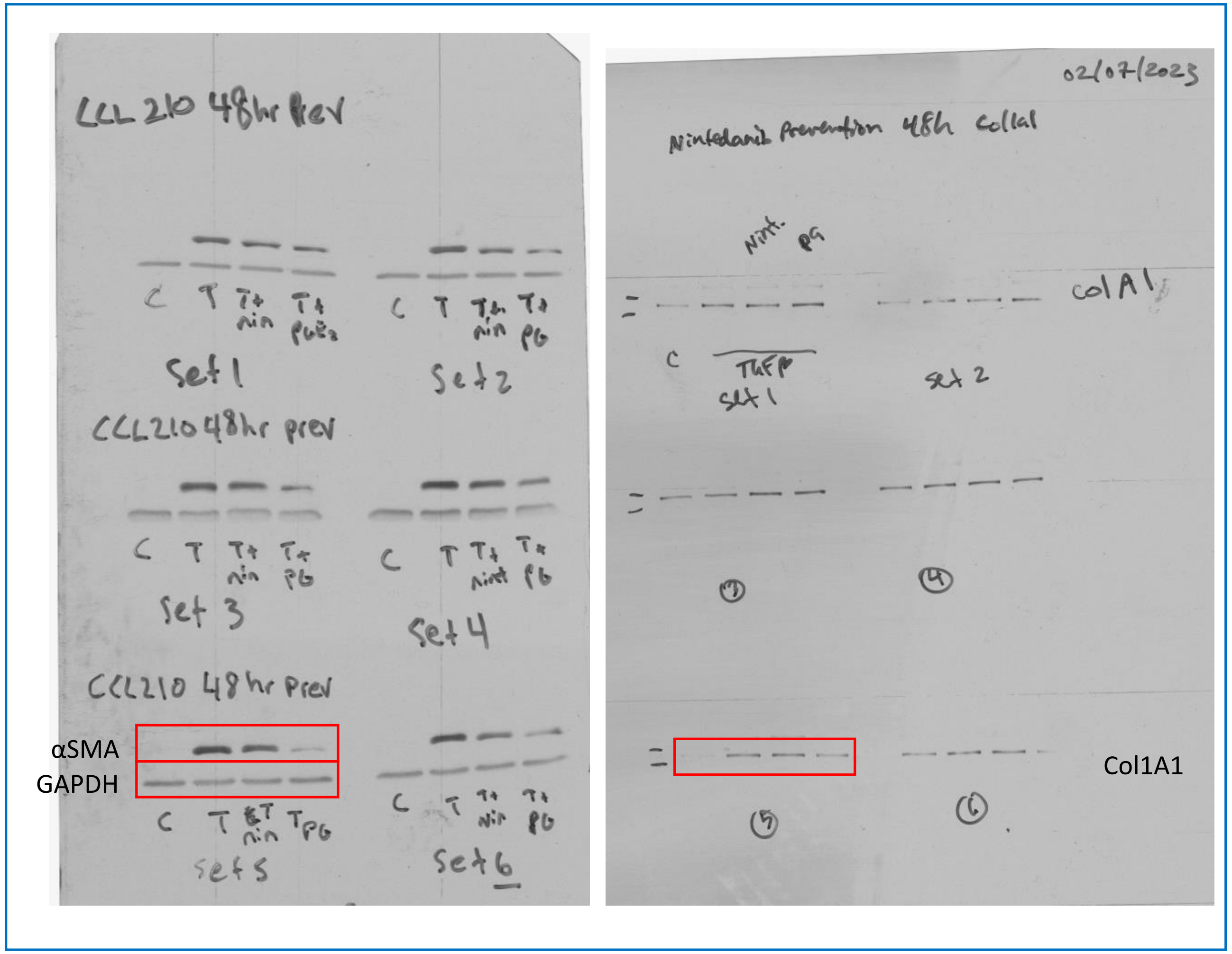

GAPDH (LEFT) was run in the same lanes on the same gel as the Col1a1 bands (RIGHT). The membrane was cut and developed separately.

Full unedited blots for Figure 8C: Reversal – Bottom

The membrane was probed with p-p38 primary antibody followed by anti-rabbit HRP secondary antibody and developed. The membrane was then stripped and probed with total p38 primary followed by anti-rabbit HRP secondary and developed.

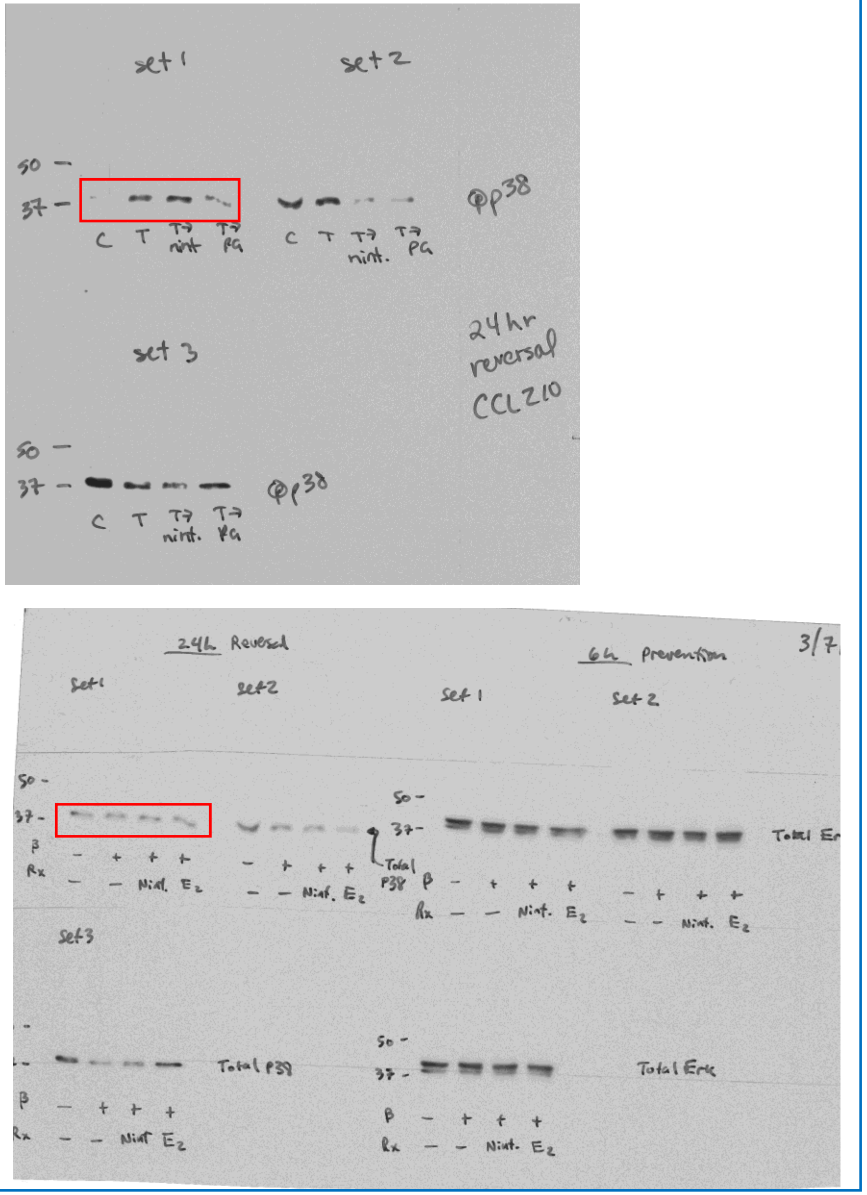

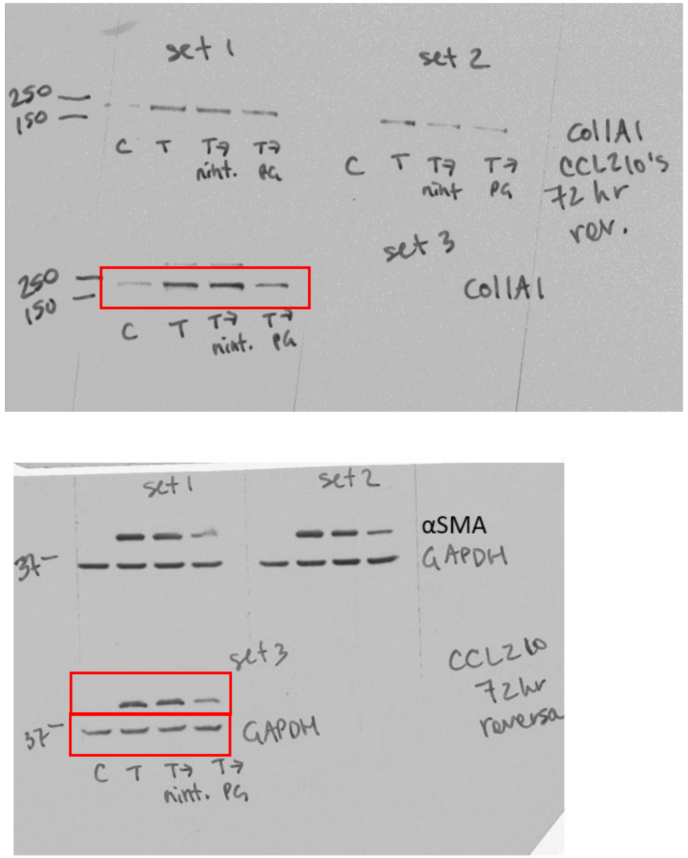

GAPDH (BOTTOM) was run in the same lanes on the same gel as the Col1a1 bands (TOP). The membrane was cut and developed separately.

Full unedited blots for Supplemental Figure 1F:

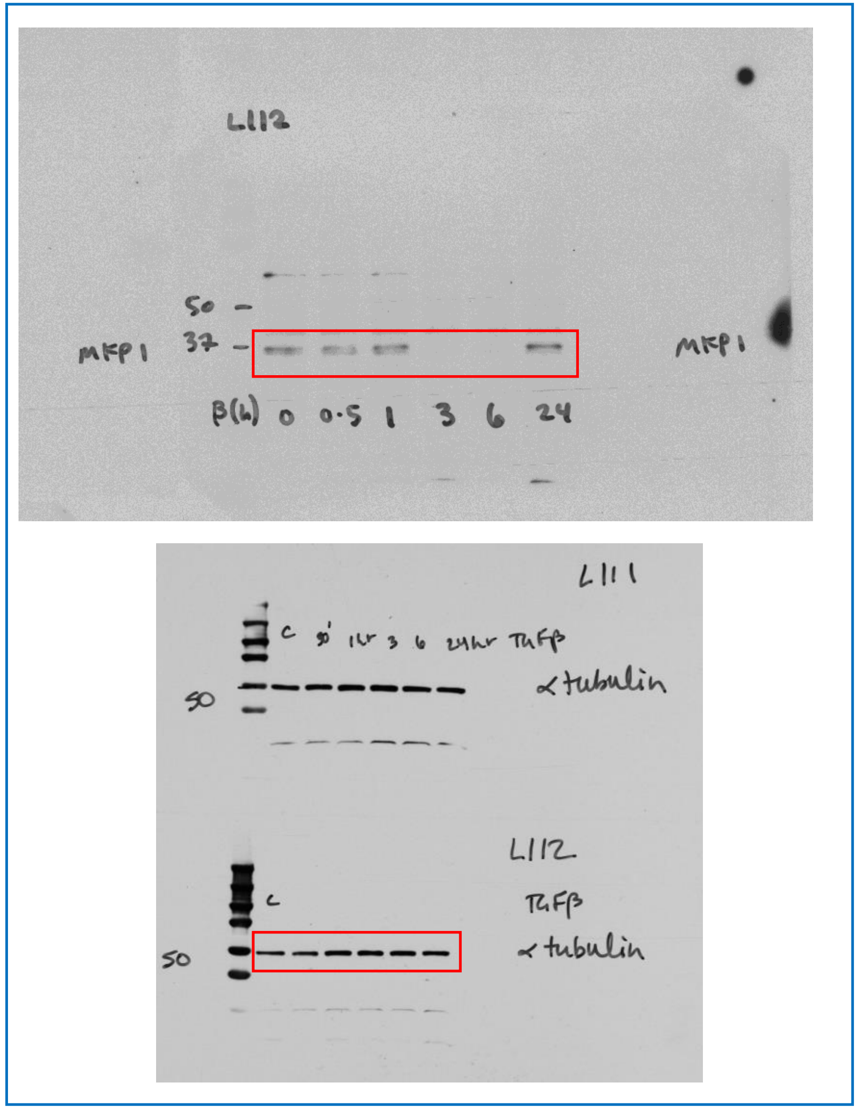

Full unedited blots for Supplemental Figure 5A:

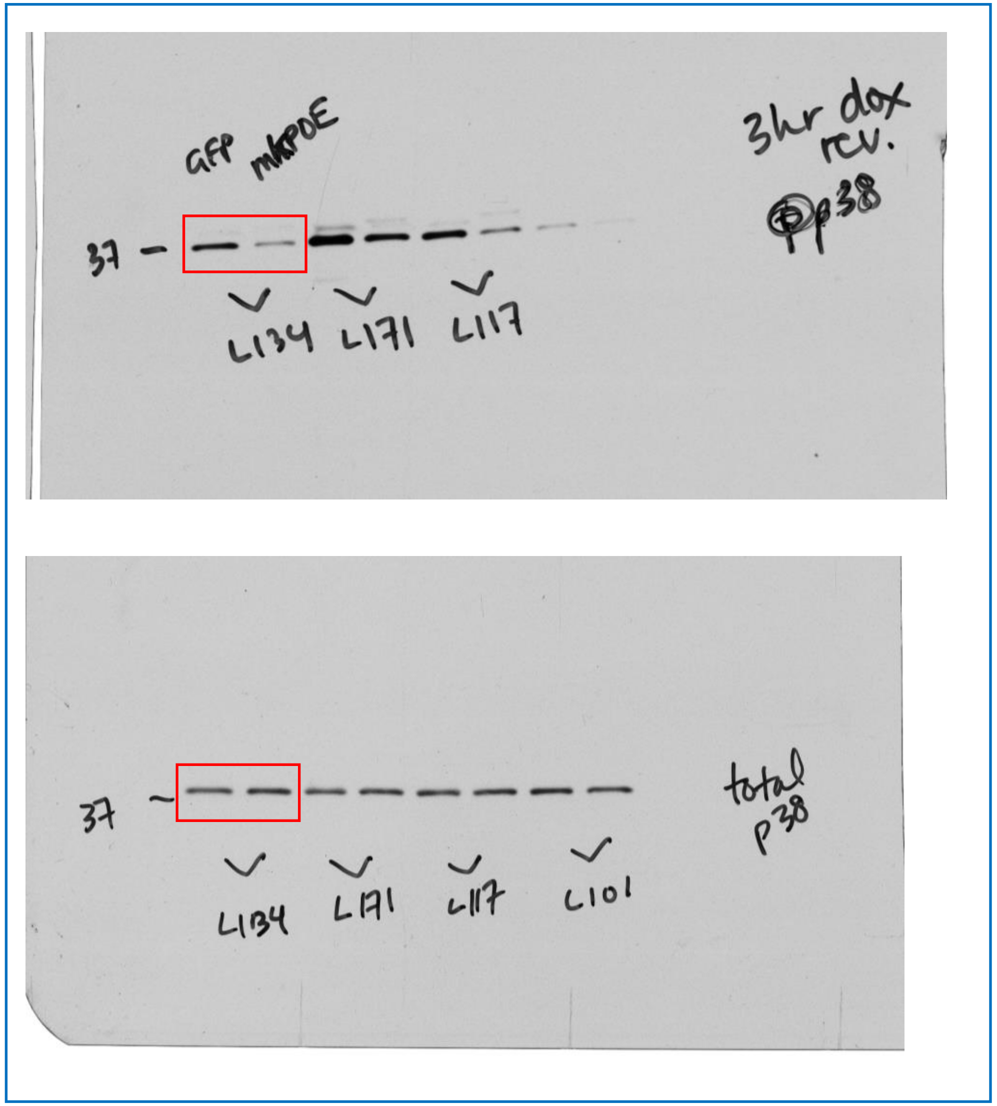

The membrane was probed with p-p38 primary antibody followed by anti-rabbit HRP secondary antibody and developed. The membrane was then stripped and probed with total p38 primary followed by anti-rabbit HRP secondary and developed.

Full unedited blots for Supplemental Figure 6B: total p38 and alpha-tubulin

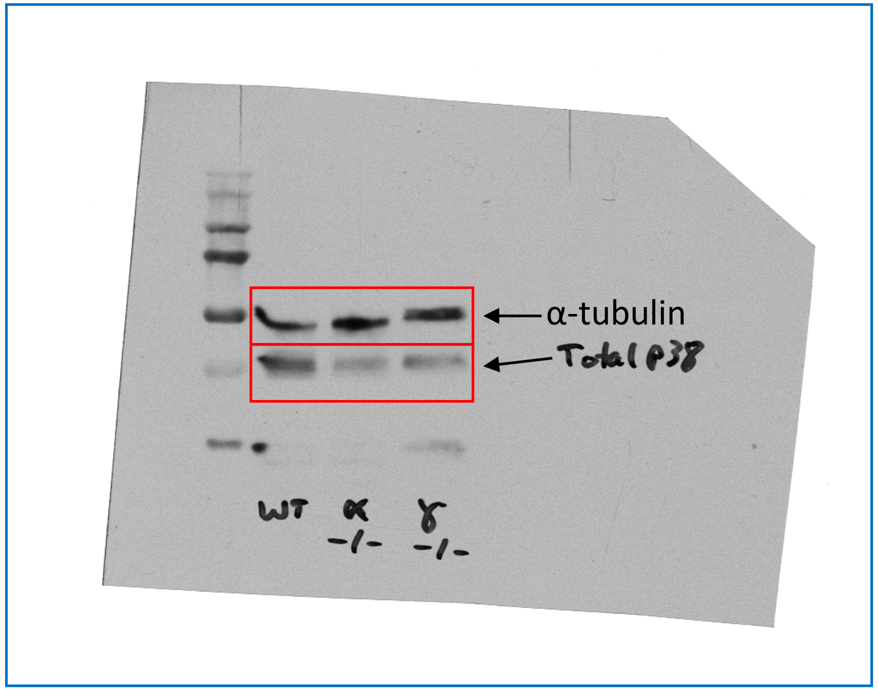

Full unedited blots for Supplemental Figure 6B: p38 alpha and p38 gamma

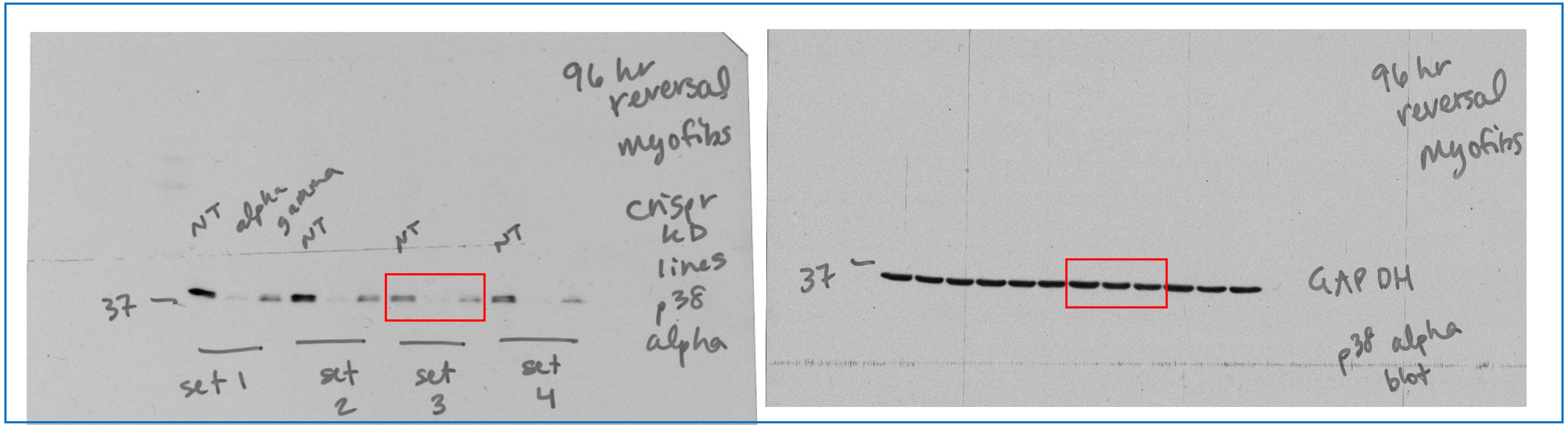

TOP: The membrane was probed with p38-alpha primary antibody followed by anti-rabbit HRP secondary antibody and developed. The membrane was then stripped and probed with GAPDH primary HRP antibody and developed.

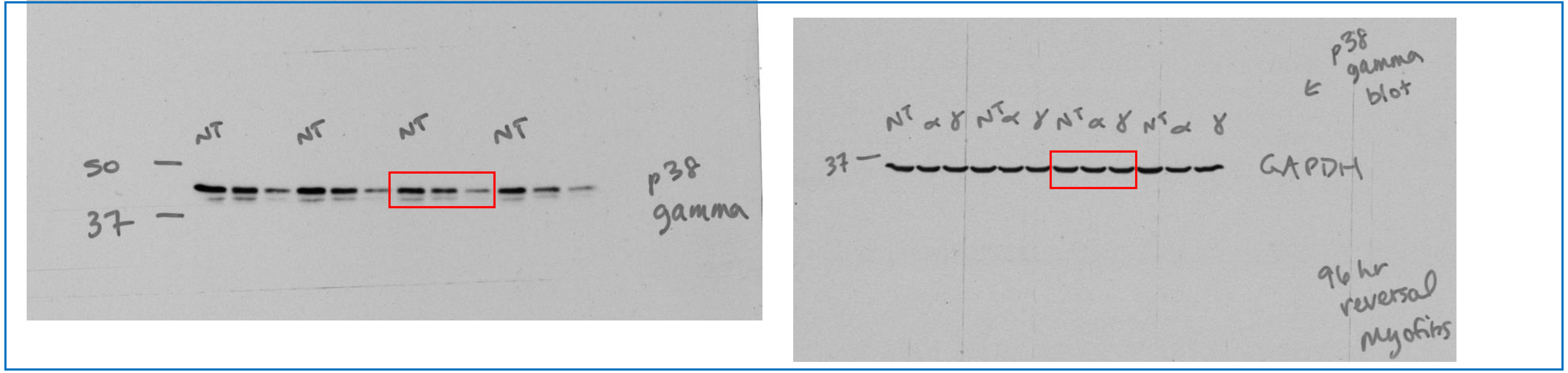

BOTTOM: The membrane was probed with p38-gamma primary antibody followed by anti-rabbit HRP secondary antibody and developed. The membrane was then stripped and probed with GAPDH primary HRP antibody and developed.

Full unedited blots for Supplemental Figure 6D:

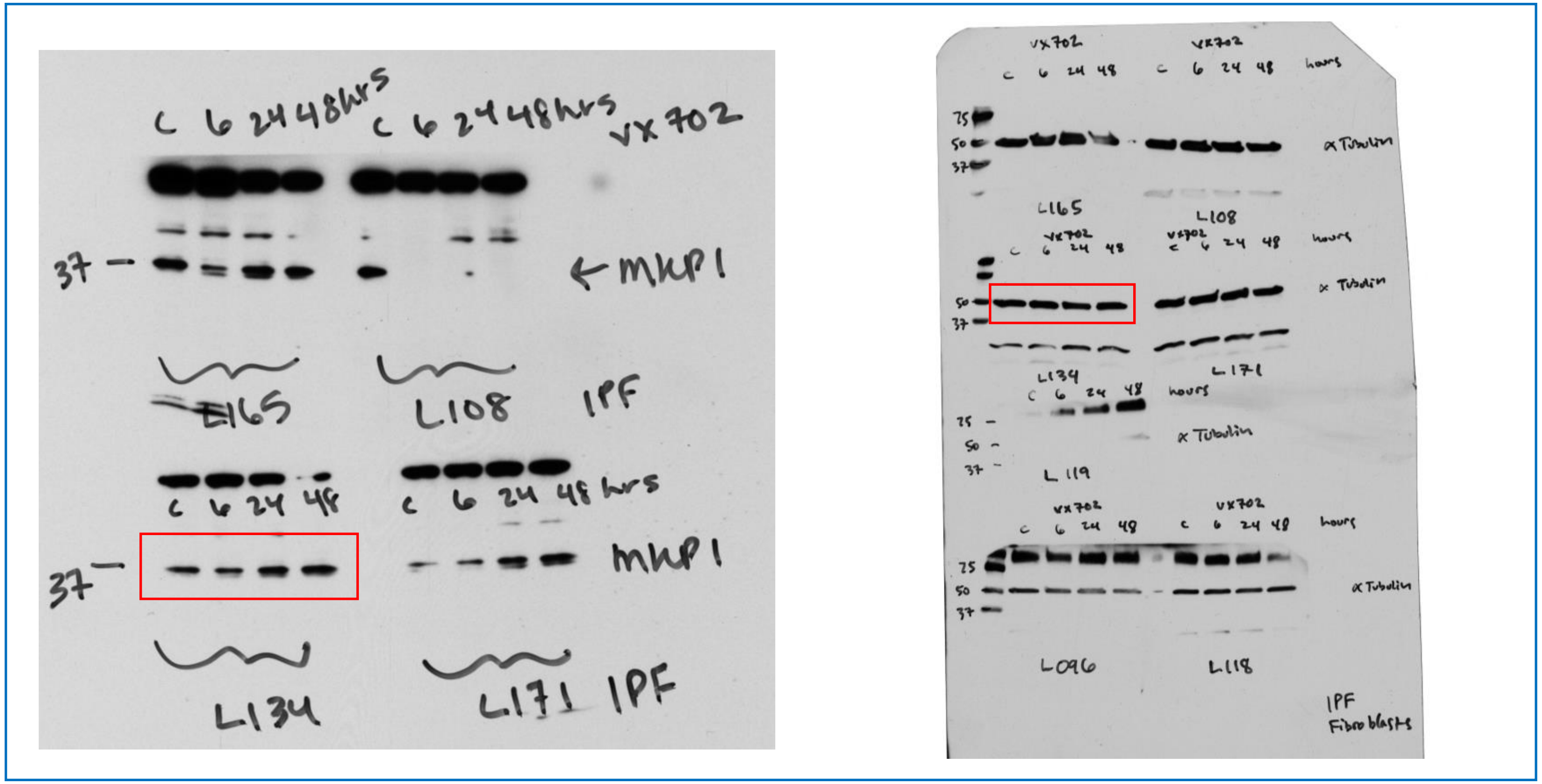

Full unedited blots for Supplemental Figure 6E:

GAPDH (BOTTOM) was run in the same lanes on the same gel as the Col1a1 bands (TOP). The membrane was cut and developed separately.

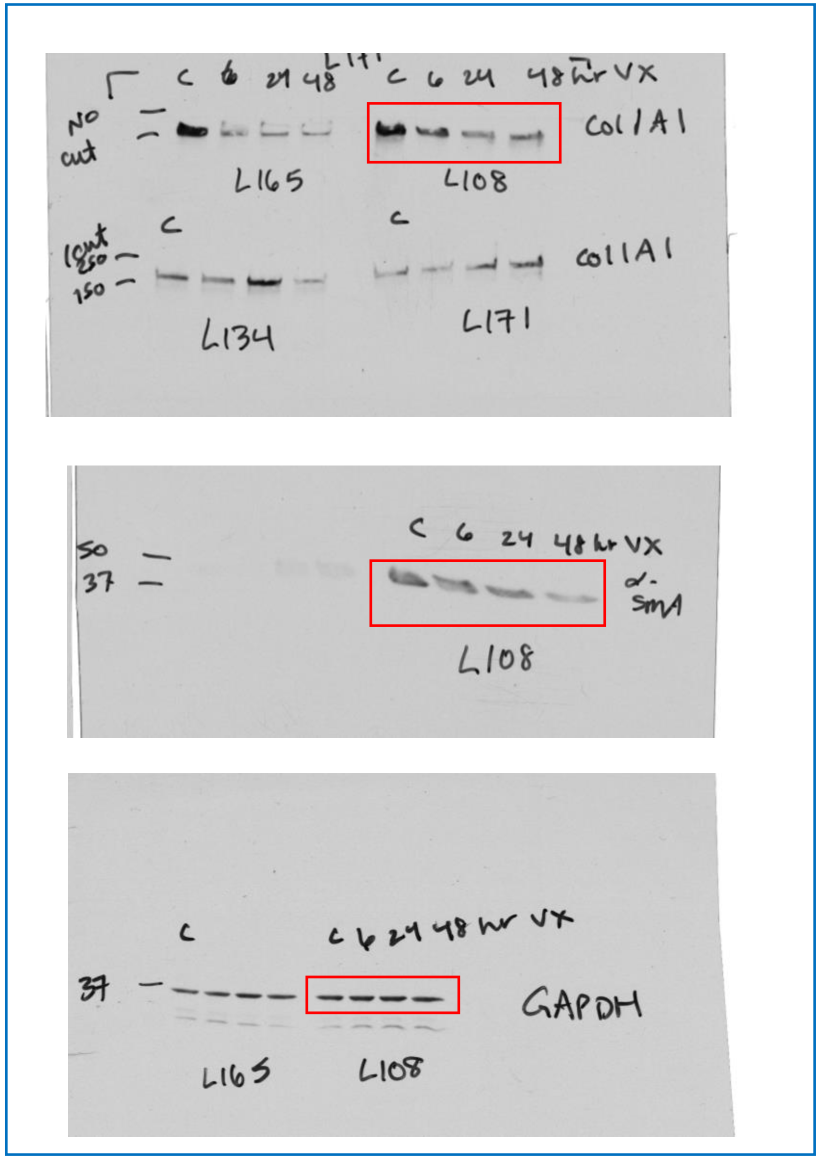

Compressed vertically in final Figure to fit into cropped image

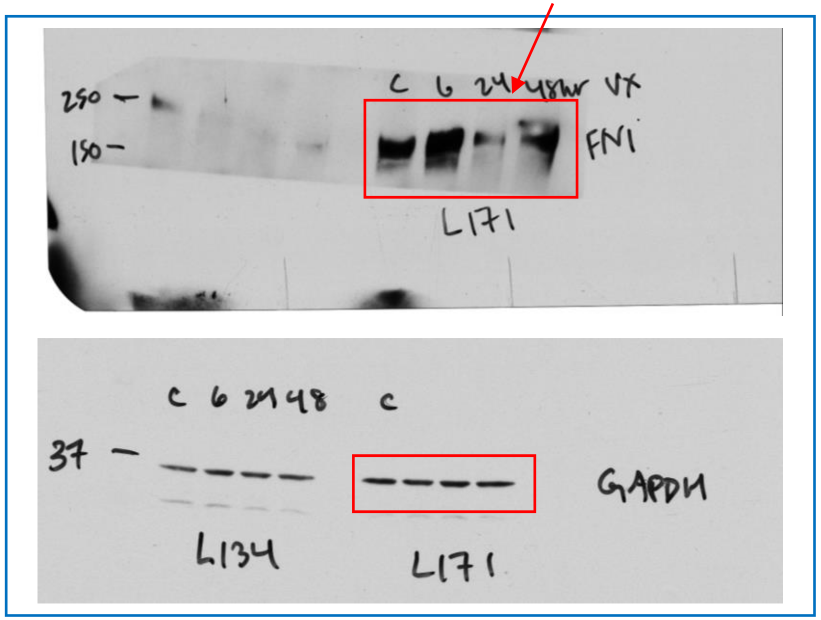

GAPDH (BOTTOM) was run in the same lanes on the same gel as the FN1 bands (TOP). The membrane was cut and developed separately.

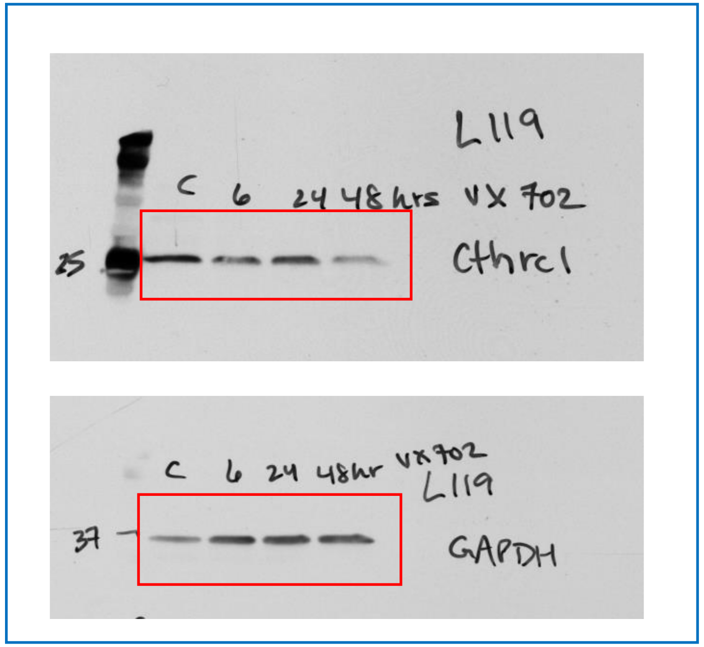

Full unedited blots for Supplemental Figure 8A:

The membrane was probed with p-p38 primary antibody followed by anti-rabbit HRP secondary antibody and developed. The membrane was then stripped and probed with total p38 primary followed by anti-rabbit HRP secondary and developed.

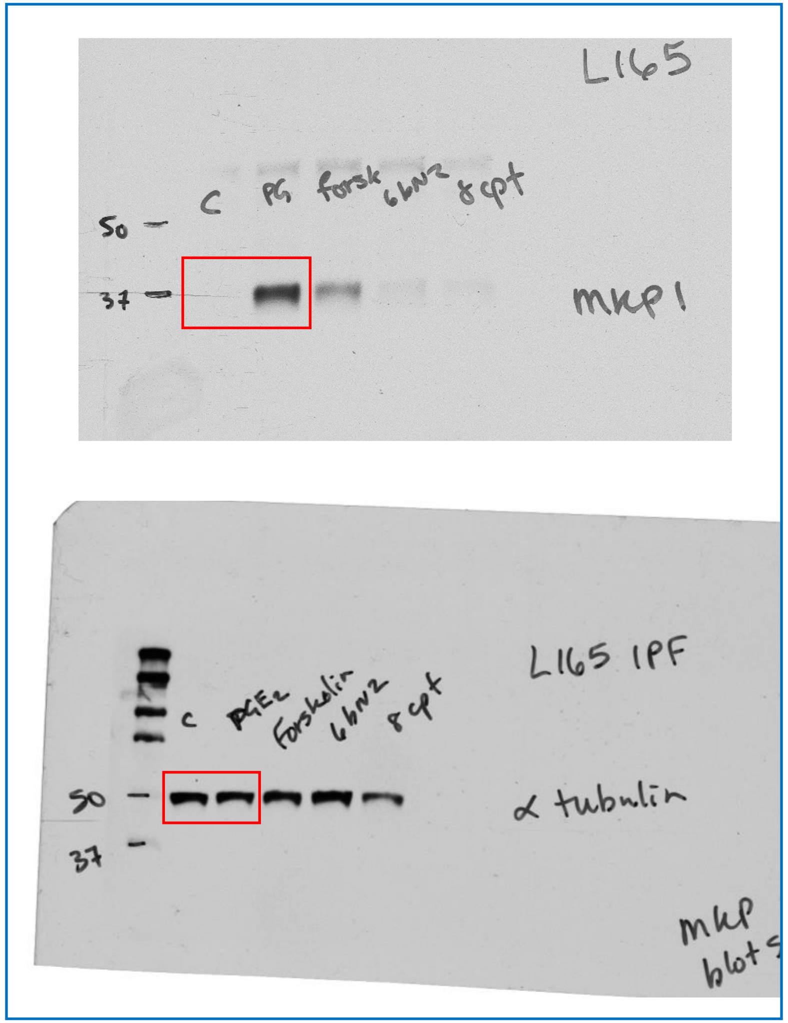

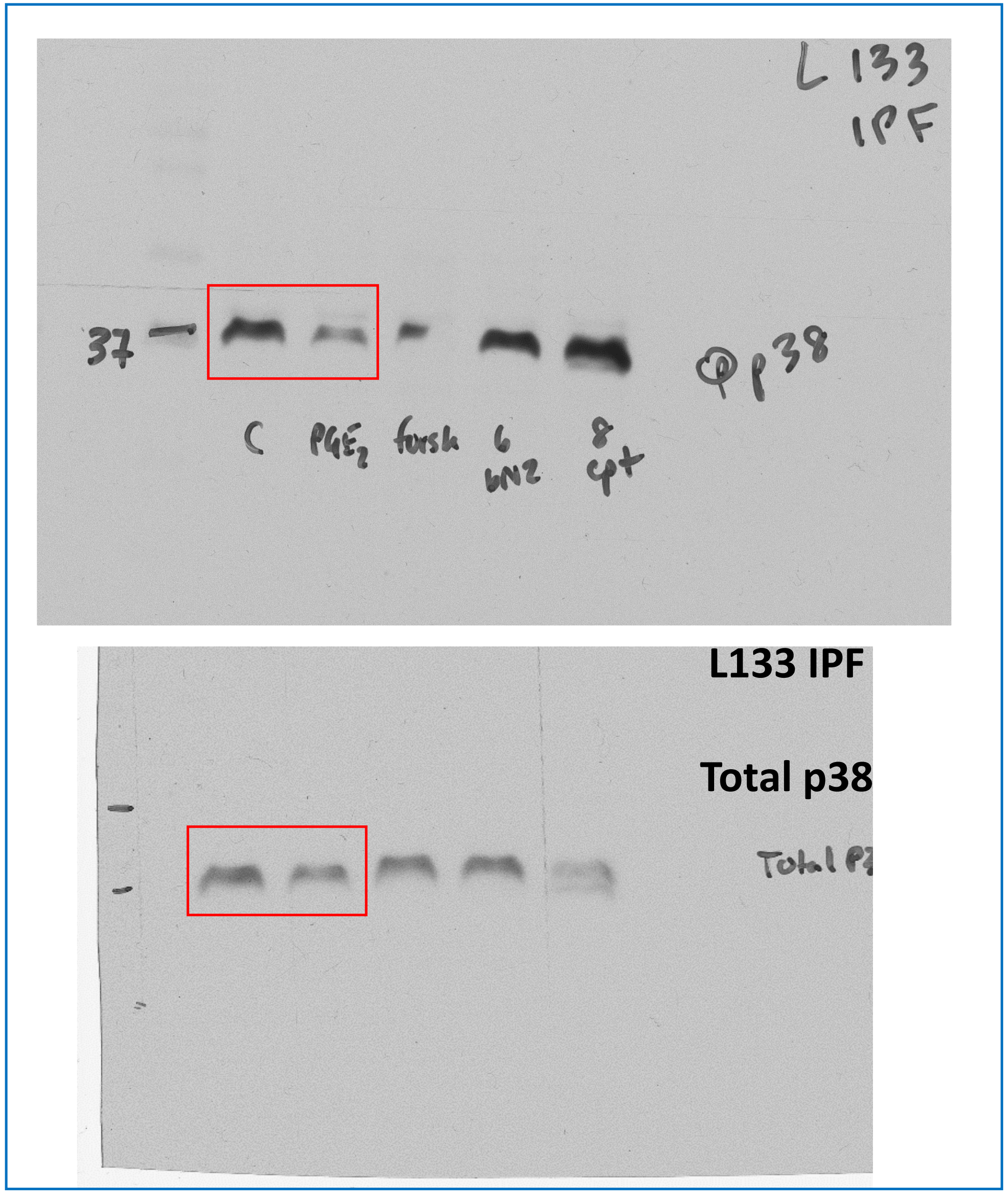

Full unedited blots for Supplemental Figure 9:

S9A

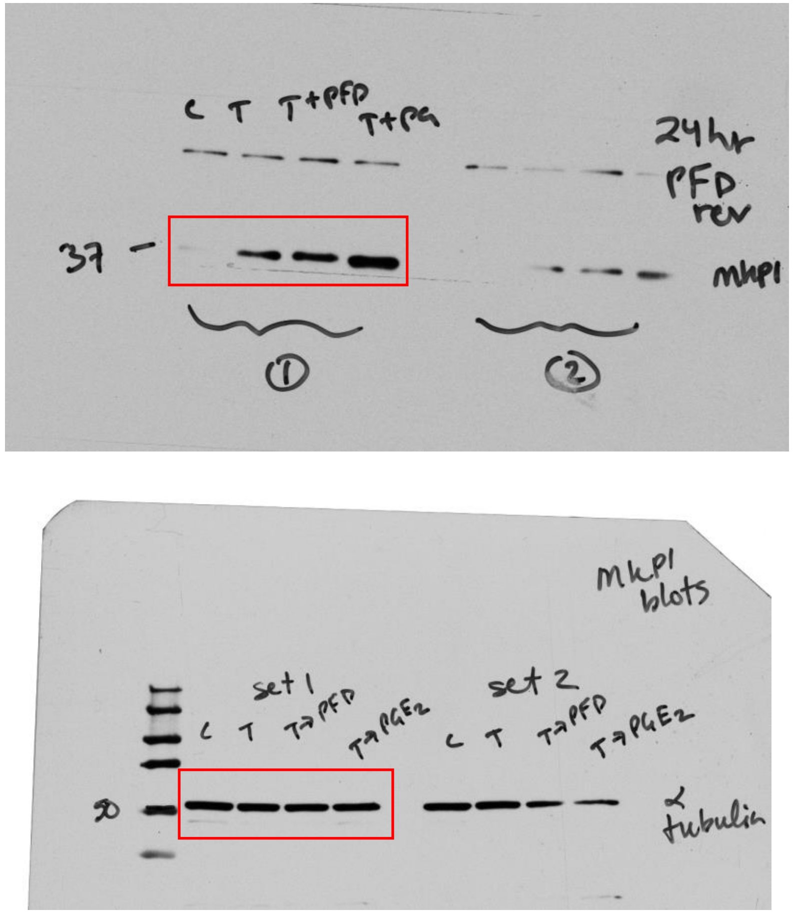

S9B

The membrane was probed with p-ERK primary antibody followed by anti-rabbit HRP secondary antibody and developed. The membrane was then stripped and probed with total ERK primary followed by anti-rabbit HRP secondary and developed.

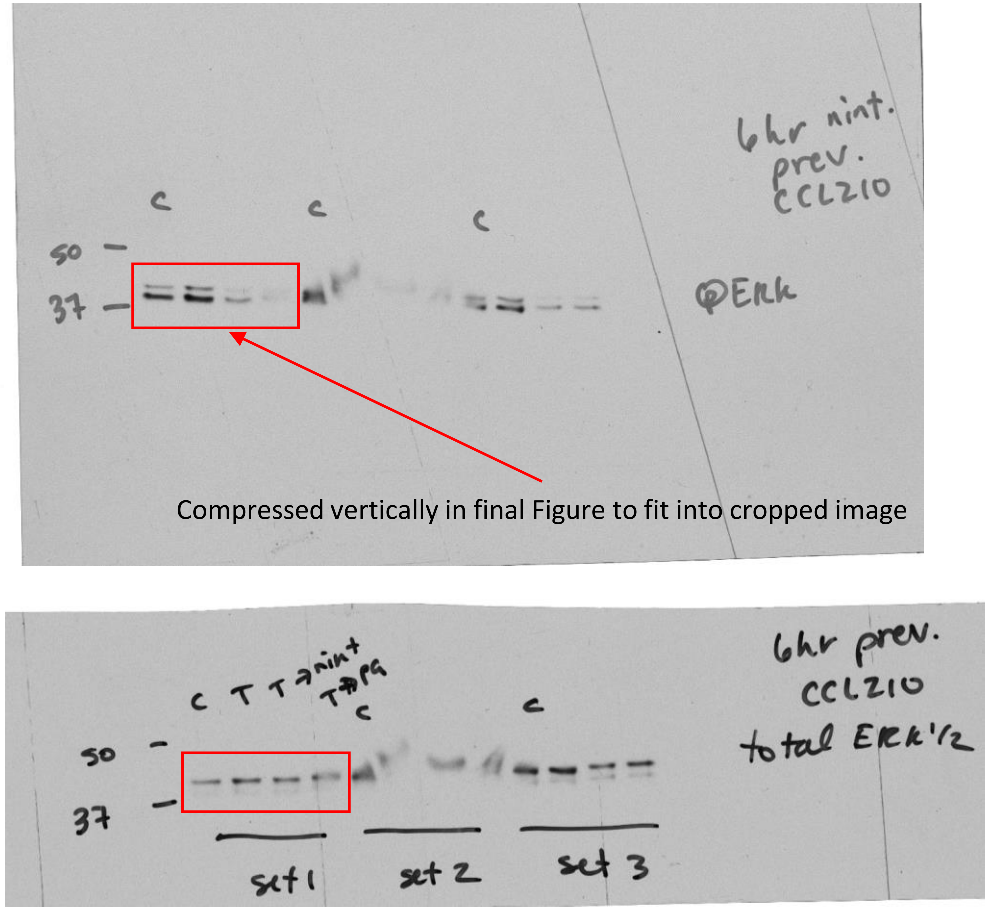

Supplement: Unedited blot and gel images [file jci-134-172826-s119.pdf]
